# ECSIT Is a Critical Factor for Controlling Intestinal Homeostasis and Tumorigenesis through Regulating the Translation of YAP Protein

**DOI:** 10.1002/advs.202205180

**Published:** 2023-07-06

**Authors:** Yuying Jiang, Chunmei Ma, Yingchao Hu, Yongbing Yang, Chanyuan Ma, Chunyan Wu, Lu Liu, Shuang Wen, Paul N. Moynagh, Bingwei Wang, Shuo Yang

**Affiliations:** ^1^ Department of Immunology State Key Laboratory of Reproductive Medicine and Offspring Health Jiangsu Key Lab of Cancer Biomarkers Prevention and Treatment Collaborative Innovation Center for Personalized Cancer Medicine Gusu School The Affiliated Wuxi People's Hospital of Nanjing Medical University Wuxi People's Hospital Wuxi Medical Center Nanjing Medical University Nanjing 211166 China; ^2^ Kathleen Lonsdale Institute for Human Health Research Department of Biology National University of Ireland Maynooth Maynooth W23 F2H6 Ireland; ^3^ Wellcome‐Wolfson Institute for Experimental Medicine Queen's University Belfast Belfast BT7 1NN UK; ^4^ Department of Pharmacology Nanjing University of Chinese Medicine 138 Xianlin Avenue Nanjing 210023 China

**Keywords:** evolutionarily conserved signaling intermediate in Toll pathways, intestinal differentiation and tumorigenesis, YAP

## Abstract

The intestinal epithelium is the fastest renewing tissue in mammals and its regenerative process must be tightly controlled to minimize the risk of dysfunction and tumorigenesis. The orderly expression and activation of Yes‐associated protein (YAP) are the key steps in driving intestinal regeneration and crucial for intestinal homeostasis. However, the regulatory mechanisms controlling this process remain largely unknown. Here, it is discovered that evolutionarily conserved signaling intermediate in Toll pathways (ECSIT), a multi‐functional protein, is enriched along the crypt–villus axis. Intestinal cell‐specific ablation of ECSIT results in the dysregulation of intestinal differentiation unexpectedly accompanied with enhanced YAP protein dependent on translation, thus transforming intestinal cells to early proliferative stem “‐like” cells and augmenting intestinal tumorigenesis. Loss of ECSIT leads to metabolic reprogramming in favor of amino acid–based metabolism, which results in demethylation of genes encoding the eukaryotic initiation factor 4F pathway and their increased expression that further promotes YAP translation initiation culminating in intestinal homeostasis imbalance and tumorigenesis. It is also shown that the expression of ECSIT is positively correlated with the survival of patients with colorectal cancer. Together, these results demonstrate the important role of ECSIT in regulating YAP protein translation to control intestinal homeostasis and tumorigenesis.

## Graphical abstract

The study provides a molecular framework that defines the importance of evolutionarily conserved signaling intermediate in Toll pathways (ECSIT) in regulating Yes‐associated protein (YAP) protein translation to control intestinal tumorigenesis: Loss of ECSIT leads to metabolic reprogramming in favor of amino acid‐based metabolism, which results in demethylation of genes encoding the eukaryotic initiation factor 4F pathway and further promotes YAP translation initiation culminating in intestinal homeostasis imbalance and tumorigenesis.

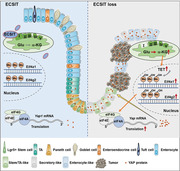

## 1 Introduction

The intestinal epithelium is a rapidly‐renewing cellular compartment. This constant regeneration along the crypt–villus axis is a hallmark of intestinal homeostasis and requires a tightly regulated balance between intestinal stem cell (ISC) proliferation and differentiation.^[^ ^1^ ^]^ The dysfunction of intestinal homeostasis drives the development of intestinal inflammation or tumorigenesis.^[^ ^2^ ^]^ While the Lgr5^+^ stem cell has been reported as the progenitor of all intestinal cell types, many studies have demonstrated that there were previously existing Lgr5^−^ cells indispensable for intestinal homeostasis and regeneration during the initiation of intestinal development.^[^ ^3^ ^]^ Moreover, recent studies suggested that the initial regenerative process in the intestine relies on transient expression and activation of the Yes‐associated protein (YAP), and this event occurs in Lgr5^−^ cells.^[^ ^3^, ^4^ ^]^ Only following YAP action, Lgr5^+^ stem cells can be induced to appear. This highlights the importance of precisely regulating YAP protein in the initiation of intestinal differentiation and development. However, the mechanism to regulate YAP protein expression in the context of intestinal homeostasis remains to be elucidated.

Evolutionarily conserved signaling intermediate in Toll pathways (ECSIT) was first described as a key protein in early embryonic development during mesoderm formation.^[^ ^5^ ^]^ Several reports indicate its innate immune roles in the activation of NF‐*κ*B signaling^[^ ^6^ ^]^ and production of mitochondrial reactive oxygen species (mROS) to enhance bactericidal activity in response to *Salmonella* infection.^[^ ^7^ ^]^ Additionally, the studies also indicate that ECSIT is an important assembly factor for mitochondrial complex I^[^ ^8^ ^]^ and plays a role in cardioprotection^[^ ^9^ ^]^ and mitophagy‐dependent mitochondrial quality control.^[^ ^10^ ^]^ Thus, ECSIT is a multi‐functional protein that has important roles in many physiological functions. However, its physiological role in the intestine remains undefined.

In this study, we found that ECSIT was enriched along the crypt–villus axis and is essential for maintaining intestinal differentiation homeostasis. Deletion of ECSIT in intestinal epithelial cells leads to dysfunctional intestinal differentiation accompanied by metabolic reprogramming to amino acid–based metabolism. This results in DNA demethylation of the genes encoding eukaryotic initiation factor 4F (eIF4F) components, which in turn, results in their increased expression and translational upregulation of the YAP protein. This metabolic reprogramming transforms intestinal cells to early proliferative stem “‐like” cell type marked by high YAP protein and downstream YAP transcriptional programs. Thus, our study is the first to provide a molecular framework for the regulation of YAP protein translation by ECSIT and identify the role of ECSIT in controlling intestinal homeostasis imbalance and tumorigenesis as a critical regulator for intestinal protection.

## 2 Results

### 2.1 Identification of ECSIT as the Enriched Factor along the Crypt–Villus Axis

**Figure 1:**
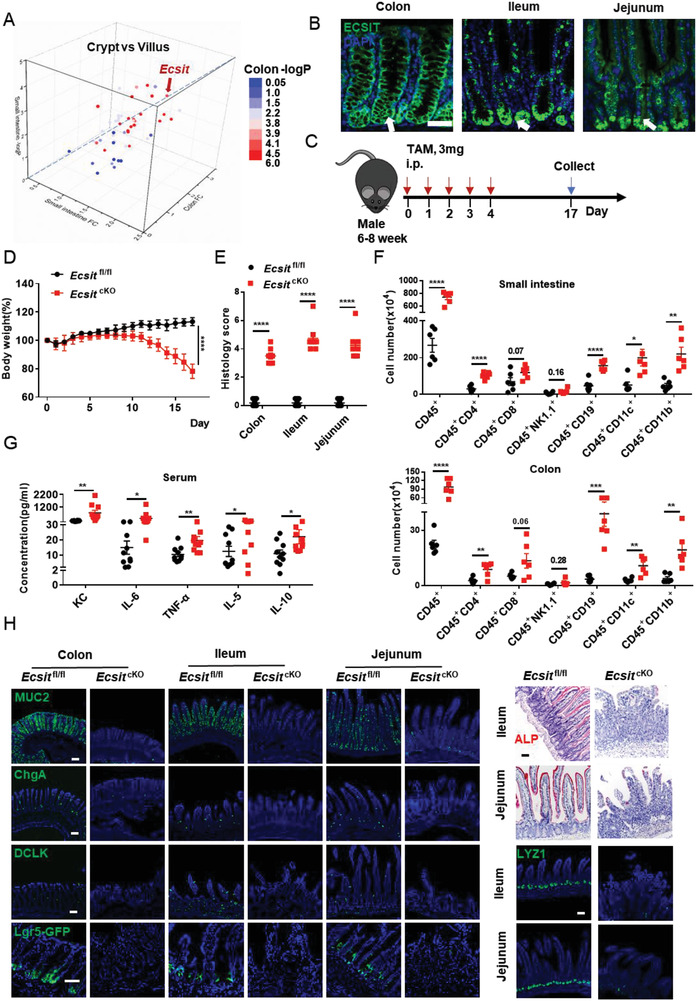
ECSIT is essential for intestinal homeostasis. A) Scatter plot showing fold change and ‐log_10_‐transformed *P* value of genes related to mitochondrial complex I assembly differentially expressed between crypt and villus in both colon and small intestine from WT mice (*n* = 3). B) Immunofluorescence staining for ECSIT expression (green, indicated by arrows) of colon, ileum, and jejunum from WT mice. Nuclei were labeled with DAPI (blue). Scale bar, 100 µm. C) Schematic of tamoxifen treatment strategy for age‐matched male *Ecsit* ^fl/fl^ and *Ecsit* ^fl/fl^ *Villin‐Cre ER* ^T2^ (hereafter *Ecsit* ^cKO^) mice, intraperitoneal (i.p.) injection of 3 mg tamoxifen for five consecutive days, the mice were euthanized 17 days after the first injection for phenotype analysis. D) Changes in body weight of tamoxifen‐treated *Ecsit* ^fl/fl^ (*n* = 8) and *Ecsit* ^cKO^ littermates. E) Histology scores of indicated mice (*n* = 8) on day 17 after the first tamoxifen injection. F) Flow cytometric analysis of the number of infiltrated immune cells in the colon and small intestine from indicated mice (*n* = 6) on day 17 after the first tamoxifen injection. G) Serum cytokines of indicated mice (*n* = 10) on day 17 after first tamoxifen injection. H) Representative images of colon, ileum, and jejunum from the indicated mice stained with the indicated antibodies or Alp (Alkaline phosphatase). For Lgr5‐GFP staining, *Villin‐Cre ER* ^T2^ and *Ecsit* ^cKO^ mice were crossed with *Lgr5‐CreER* ^T2^‐GFP mice. Scale bars, 100 µm. (D–F) Data are pooled from three independent experiments. (B,H) Data are representative of three independent experiments. Error bars show mean ± SEM. *P ≤ 0.05, **P ≤ 0.01, ***P ≤ 0.001, ****P ≤ 0.0001. Two‐way ANOVA test for (D). Two‐tailed unpaired student's *t*‐test for (E–G).

While comparing the gene expression profiles of the intestinal crypt and villus regions of mice by RNA‐seq analysis (Figure S1A, Supporting Information), interestingly we found that mitochondrial complex I (Figure S1B, Supporting Information), and particularly, mitochondrial complex I assembly (Figure S1C, Supporting Information) were the most significantly upregulated gene ontology (GO) terms in the crypt versus villus comparison for both small intestine and colon. This is consistent with previous reports that mitochondrial biogenesis is tightly distributed along the intestinal crypt–villus axis.^[^ ^11^ ^]^ Due to this enriched distribution in the crypt region, we were motivated to study the components of the complex I assembly machinery in detail and found that ECSIT, a multi‐functional protein, is the most upregulated factor in the crypt versus villus (**Figure** **1**A). Immunofluorescence (IF) analysis also showed that ECSIT was predominantly enriched at the middle and bottom regions of the crypt–villus axis (Figure 1B), suggesting its potentially important role in the regulation of intestinal differentiation homeostasis.

### 2.2 Dysregulation of Intestinal Differentiation after ECSIT Deletion in Intestinal Cells

To investigate the specific role of ECSIT in intestinal homeostasis, we generated *Ecsit* ^fl/fl^ mouse models and crossed them with *Villin‐Cre* mice to conditionally knock out *Ecsit* in the intestinal epithelium (Figure S2A–E, Supporting Information). Such conditional deletion of ECSIT resulted in lower survival rates (Figure S3A, Supporting Information), smaller size body size and weights in both genders (Figure S3B, Supporting Information), increased spleen size (Figure S3C, Supporting Information), and notably spontaneous intestinal inflammation as evidenced by colon shortening (Figure S3D, Supporting Information), increased infiltration of inflammatory cells, as well as morphological and structural disorder of colon, ileum, and jejunum (Figure S3E,F, Supporting Information). To further examine whether loss of ECSIT after development impairs intestinal homeostasis, we engineered a *Ecsit* ^floxed/^ *Vil* *l* *in‐Cre‐ER* ^T2^ mouse model (Figure S2D, Supporting Information), and injected tamoxifen (3 mg) intraperitoneally for five consecutive days to achieve conditional deletion of ECSIT in the intestinal epithelium at specific times after attaining adulthood (Figure 1C and Figure S2F, Supporting Information). The mice were euthanized 17 days after the first injection for phenotypic analysis. These *Ecsit* ^fl/fl^ *Vi* *ll* *in‐Cre‐ER* ^T2^ mice (cKO) also showed spontaneous intestinal inflammation that was associated with weight loss (Figure 1D), shortening of the colon (Figure S4A, Supporting Information), intestinal structure disorders (Figure 1E and Figure S4B, Supporting Information), increased infiltration of inflammatory cells (Figure 1F), and increased serum inflammatory cytokines (Figure 1G). Interestingly, at the same time, functional cell types including ALP^+^ enterocytes, MUC2^+^/PAS goblet cells, ChgA^+^ enteroendocrine cells, LYZ1^+^ Paneth cells, and DCLK^+^ Tuft cells were significantly reduced, and even Lgr5^+^ and OLFM4^+^ stem cells were reduced as shown by immunofluorescence (IF) and histological analysis (Figure 1H and Figure S4C–E, Supporting Information), which suggests that the loss of ECSIT impaired intestinal differentiation.

To confirm that the specificity of this phenotype was due to the selective deletion of the *Ecsit* gene, we generated *Ecsit* ^fl/fl^ *Ecsit*‐KI‐3Flag^fl/+^ *Vil* *l* *in‐Cre‐ER* ^T2^ mice (Figure S5A–D, Supporting Information) and found that the reconstitution of ECSIT expression in the intestine rescued the dysfunctional phenotype in ECSIT‐deficient mice (Figure S5E–G, Supporting Information). Additionally, *Ecsit* ^fl/fl^ *Lgr5‐CreER* ^T2^‐GFP mouse models were built and observed to have reduced number of differentiated LYZ1^+^ Paneth cells and Lgr5^+^ stem cells, 17 days after the first tamoxifen injection (Figures S2D and S4F,G, Supporting Information). We also explored the long‐term phenotype in this model, wherein we analyzed the phenotype of *Ecsit* ^fl/fl^ *Lgr5‐CreER* ^T2^‐GFP mice, 2 months after the first tamoxifen injection. However, we found that the morphology and inflammatory pathology of the intestine had not changed (Figure S4H,I, Supporting Information). We speculated that due to incomplete knockout, this model may create mosaic mice in which undifferentiated cells derived from ECSIT null ISCs may be ultimately outcompeted by WT ISCs. Collectively, these results indicate that the absence of ECSIT in the intestine leads to the dysregulation of intestinal differentiation.

### 2.3 Single‐Cell Survey of ECSIT‐Deficient Intestinal Cells Reveals an Early Proliferative Stem “‐Like” Phenotype

**Figure 2:**
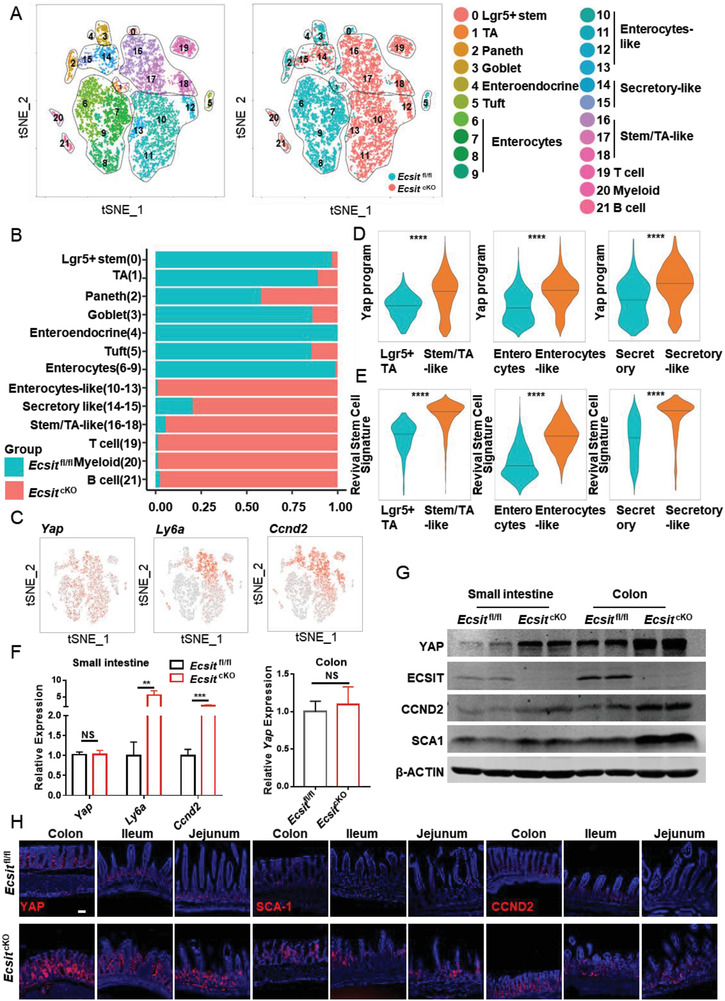
ECSIT‐deficient intestinal cells display an early proliferative stem “‐like” phenotype characterized by high YAP signature and regeneration. A) Unsupervised clustering of scRNA‐seq data in whole epithelia of small intestine from *Ecsit* ^fl/fl^ and *Ecsit* ^cKO^ mice on day 17 after first tamoxifen injection (*n* = 12431 single‐cell transcriptomes, from two independent experiments, in each experiment, cells were mixed from 3 mice). Unsupervised clusters are overlaid on the t‐SNE map and are indicated by different colors and labeled according to their identity, based on the expression of cell‐type–specific marker‐gene expression (left). Cells from *Ecsit* ^fl/fl^ (blue dots) or *Ecsit* ^cKO^ intestinal epithelium (red dots) are also plotted as indicated (right). B) Bar graph showing the frequency of *Ecsit* ^fl/fl^ (blue) or *Ecsit* ^cKO^ (red) cells of each cell type in small intestinal epithelium. C) Expression of indicated genes is plotted on a t‐SNE graph of small intestinal epithelium. D) ssGSEA analysis for YAP program signature of indicated cell type in small intestinal epithelium. E) ssGSEA analysis for revival stem cell signature of indicated cell type in small intestinal epithelium. F) qPCR analysis of indicated gene expression in small intestinal and colonic epithelium (*n* = 6). G) Immunoblotting analysis of ECSIT, YAP, CCND2, SCA1, and *β*‐ACTIN (loading control) in intestinal epithelium from indicated mice on day 17 after first tamoxifen injection. H) Representative images of colon, ileum, and jejunum from the indicated mice stained with the indicated antibodies. Scale bar, 100 µm. (G,H) Data are representative of three independent experiments. Error bars show mean ± SEM. **P ≤ 0.01, ***P ≤ 0.001, ****P ≤ 0.0001, NS, not significant. Two‐tailed unpaired student's *t*‐test.

Next, we performed single cell RNA‐seq (scRNA‐seq) analysis on small intestinal and colonic epithelia from ECSIT‐deficient mice to detect the effect of ECSIT loss on the differentiation homeostasis of intestinal cells 17 days after the first TAM injection. The unsupervised clustering approach was used to partition the cells into 22 clusters in the small intestine (**Figure** **2**A; Figures S6A–C and S7A, Supporting Information) and 18 clusters in the colon (Figures S8A–C and S9A, Supporting Information), which we visualized using t‐distributed stochastic neighborhood embedding (t‐SNE) and labeled by known markers for the cell subtypes. The analysis identified the various intestinal cell types as described in a previous report,^[^ ^12^ ^]^ including enterocytes and secretory cells (including Paneth cells [only in the small intestine], tuft cells, goblet cells, and enteroendocrine cells), TA cells and Lgr5^+^ stem cells. The data from scRNA‐Seq in *Ecsit* ^fl/fl^ mice reveal that *Ecsit* expression is high in stem/TA cells, but there is also *Ecsit* expression in enterocytes, Paneth, goblet, enteroendocrine, and tuft cells (Figures S6D and S8D, Supporting Information), which is consistent with our IF data showing the enriched level of ECSIT predominantly localized at the middle and bottom regions of the crypt–villus axis (Figure 1B).

Moreover, scRNA‐seq analysis of the intestinal epithelium showed clear cell differences between ECSIT intestinal cKO mice and control (*Ecsit* ^fl/fl^) mice (Figure 2A and Figure S9A, Supporting Information). There were markedly lower percentages of various intestinal cell types both in the small intestine (Figure 2B) and in the colon (Figure S9B, Supporting Information) of cKO mice compared with control mice. This is wholly consistent with the earlier IF analysis (Figure 1H and Figure S4E, Supporting Information). In contrast, we particularly found that the unique early proliferative stem “‐like” cell types appear in larger numbers in cKO mice with these cells including stem/TA‐like cells, enterocyte‐like cells, and secretory‐like cells. Interestingly, these “‐like” cells were characterized by enhanced expression of YAP target genes such as Ly6a, Ccnd2, Birc5, Sox9, Cdk4, and Anxa3, compared with their expression in corresponding stem/TA cells, enterocytes, and secretory cells (Figure 2C; Figures S6C,  S7A,  S8C, and S9C, Supporting Information), and showed significantly higher YAP program and revival stem cell signature (regulated by YAP)^[^ ^4a,b,d^ ^]^ (Figure 2D,E and Figure S9E, Supporting Information). YAP, a transcriptional co‐activator inhibited by Hippo signaling, is known to be critically important for early intestinal stem cell proliferation and intestinal epithelium regeneration.^[^ ^3^, ^4^, ^13^ ^]^ However, the overexpression of YAP protein will result in an inability to reacquire intestinal homeostasis upon tissue damage, causing the development of cancer.^[^ ^4^, ^7^ ^]^ Correspondingly, we revealed that both gene and protein levels of YAP targets such as SCA1 (encoded by Ly6a), SOX9, and CCND2, that are involved in the regulation of intestinal stem cell homeostasis and overexpressed in colorectal cancer (CRC),^[^ ^14^ ^]^ are also upregulated in the ECSIT‐deficient intestine (Figure 2F–H and Figure S7B,D, Supporting Information). We also performed histological analysis of cKO mouse intestinal tissues at earlier time points on 5 and 10 days after the first TAM injection and examined the expression of YAP and its targets, as well as markers of intestinal epithelium proliferation, differentiation, and death. We found that while cKO intestines showed no pathological inflammation or abnormal intestinal epithelium differentiation and death (Figure S10, Supporting Information), there was a significant increase in YAP protein expression and its targets such as CCND2, SCA1, and KI67 that has been existed in cKO intestines at early day 5 post TAM (Figures S10 and S11, Supporting Information). However, at day 10 post TAM, the inflammatory pathology has been appeared in cKO intestine with intestinal structure disorders and increased infiltration of inflammatory cells (Figure S10, Supporting Information). At the same time, there was a markedly increasing in YAP protein and its downstream targets in cKO intestine but a reduction of intestinal epithelium differentiation, as demonstrated by qPCR and IF analysis (Figures S10 and S11, Supporting Information). Thus, all these results clearly demonstrated that ECSIT lost first affected YAP pathway, before leading to abnormal intestinal cell differentiation and the development of intestinal pathology.

Moreover, the nuclear translocation of YAP and YAP‐TEAD target genes such as *Ctgf*, *Cyr61*, and *Areg* were also significantly upregulated in the cKO intestine tissue (Figure S12A,B, Supporting Information), which further suggests that loss of ECSIT results in an increase in YAP activation. Next, we analyzed the levels of YAP protein using proteomics, western blotting, and IF. These three independent approaches all showed remarkably higher YAP protein levels, YAP program, and revival stem cell signature in ECSIT deficient mice (Figure 2G,H; Figures S7C and S12C–E, Supporting Information). Notably, however, there was no change in Yap mRNA levels in ECSIT‐deficient intestine as determined by single cell RNA‐seq and qPCR analysis (Figure 2C,F; Figures S7D and S9C, Supporting Information). Therefore, ECSIT‐deficient intestinal cells display an early proliferative stem “‐like” cell types characterized by distinctive YAP‐responsive gene signature and regeneration characteristics, and this is associated with upregulation of YAP protein but not its encoding transcript.

### 2.4 Loss of ECSIT Facilitates Initiation of YAP Translation through the eIF4F Complex

**Figure 3:**
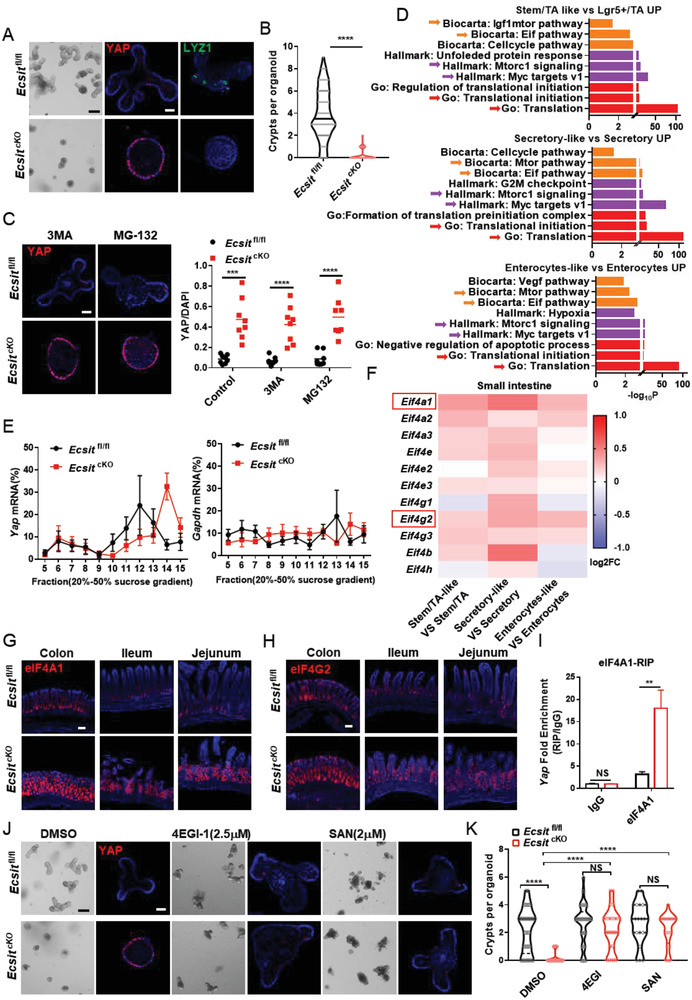
ECSIT loss facilitates YAP translation initiation through eIF4F complex. A) *Ecsit* ^fl/fl^ and *Ecsit* ^cKO^ organoids were treated with 4‐OHT for 72 h and photographed in bright‐field (BF, left), Immunofluorescent staining of YAP (red) and LYZ1 (green) in organoids (right). Nuclei were labeled with DAPI (blue). Scale bars, 100 µm (BF); 20 µm (IF). B) Quantification of crypts per organoid (*n* = 100). C) Immunofluorescent staining of YAP in organoids (left) and quantification (right, *n* = 8) after 3MA or MG‐132 treatment. Nuclei were labeled with DAPI (blue). Scale bar, 20 µm. D) Pathway enrichment analysis of indicated cell types based on scRNA‐seq data of small intestinal epithelium. E) Polysomes of indicated intestinal epithelia were extracted and subjected to 20% to 50% sucrose gradient by ultracentrifugation. 15 polysome fractions were collected from top to bottom followed by RNA extraction. YAP (left) and Gapdh (right) mRNA expressions in each fraction were determined by real‐time PCR (*n* = 5). F) Heatmap showing the indicated gene expression between cell types based on scRNA‐seq data of small intestinal epithelium. G,H) Immunofluorescence staining for eIF4A1 (G) and eIF4G2 (H) of colon, ileum, and jejunum from indicated mice. Nuclei were labeled with DAPI (blue). Scale bars, 100 µm. I) RT‐PCR analysis of Yap mRNA from RIP by eIF4A1 (*n* = 6) in intestinal epithelium. IP was performed with normal rabbit IgG as the negative control, and the data show the fold enrichment of eIF4A1 versus IgG. J) Bright‐field (BF) imaging and immunofluorescent staining of YAP from indicated treatment organoids. Nuclei were labeled with DAPI (blue). Scale bars, 100 µm (BF); 20 µm (IF). K) Quantification of crypts per organoid after 4EGI‐1 or SAN treatment (*n* = 29, 32, 33, 28, 22, and 24 in group from left to right). (A, C, G, H,J) Data are representative of three independent experiments. Error bars show mean ± SEM. **P ≤ 0.01, ***P ≤ 0.001, ****P ≤ 0.0001, NS, not significant. Two‐tailed unpaired student's *t*‐test.

Next, we cultured intestinal organoids in ENR (EGF, Noggin, and R‐Spodin‐1) medium with the aim of inducing the crypt to proceed to differentiation,^[^ ^15^ ^]^ and then explore the mechanism by which ECSIT can regulate the protein levels of YAP. Deletion of ECSIT in organoids was induced by addition of 4‐OH‐Tamoxifen (4OHT) to the culture medium. Under these culture conditions *Ecsit* ^fl/fl^ *Vil* *l* *in‐Cre‐ER* ^T2^ (cKO) organoids showed no differentiated bubble with little or no crypt formation but instead more YAP and proliferative KI67 expression (**Figure** **3**A,B and Figure S13A, Supporting Information). Moreover, nuclear translocation of YAP and levels of typical YAP target genes were significantly upregulated in the cKO organoids, suggesting that loss of ECSIT induces the over‐activation of YAP in vitro (Figure S13B,C, Supporting Information). Due to the failure of symmetry‐breaking event driven by YAP‐overexpression in cKO organoids,^[^ ^4d^ ^]^ the cKO organoids show decreased differentiation without crypt bubble formation and thus appear to be “smaller” being reminiscent of the previously described YAP‐overexpression and regenerative‐state phenotype.^[^ ^4c,d^ ^]^ Furthermore, knockdown of Yap using shYap significantly rescued the crypt formation in ECSIT‐deficient organoids (Figure S13D, Supporting Information), further indicating that YAP is responsible for the failed crypt phenotype observed in ECSIT‐deficient organoids. When organoids were treated with autophagy inhibitor (3‐MA, 5 mm) or the proteasome inhibitor (MG‐132, 5 *µ* m), the upregulation of YAP in cKO relative to control was unchanged (Figure 3C), indicating that the increased YAP protein levels in cKO intestine is not due to the impairment of protein degradation pathways.

In order to further explore the mechanism underlying augmented levels of YAP protein, we deeply analyzed the GO and pathway enrichment patterns of single‐cell transcriptome data. We noted that translation initiation, Myc targets, mTOR signaling, and Eif pathway were significantly enriched in “‐like” cells (Figure 3D; Figures S7E and S9D,E, Supporting Information), and the analysis in proteomics via GO also identified the upregulation of translation initiation signature in cKO intestine (Figure S12F,G, Supporting Information). Therefore, we speculated that the increased levels of YAP protein in cKO mice may be due to enhanced translation of YAP. Polyribosome (polysome) fractionation analysis revealed Yap mRNA translation efficiency is higher in cKO cells than in controls (Figure 3E and Figure S13E, Supporting Information). Moreover, eIF4F complex in the Eif pathway is the downstream target of mTOR signaling^[^ ^16^ ^]^ and can selectively promote the translation initiation of specific mRNAs and trigger cancer development.^[^ ^16b^ ^]^ Notably, our scRNA‐seq and IF data both showed that eIF4F members, particularly eIF4A1and eIF4G2, were significantly upregulated in cKO stem “‐like” cells compared with control cells (Figure 3F–H and Figure S9F, Supporting Information). It has been reported that eIF4A1 can specifically promote the translation of mRNAs with high GC content.^[^ ^3^, ^8^ ^]^ Interestingly, we found that the content of GC at the 5′ end of Yap mRNA is relatively high (Figure S13F, Supporting Information). RNA immunoprecipitation (RIP) analysis also confirmed greatly enriched binding of eIF4A1 binding to 5′ Yap mRNA in cKO intestine (Figure 3I).

To further examine whether eIF4F is required for the enhanced YAP protein and organoids phenotype in cKO, eIF4F inhibitors were used in intestinal organoids experiments. Treatment of cKO organoids with the eIF4A1 inhibitor sanguinarine chloride (SAN, 2.5 *µ* m),^[^ ^1^ ^]^ or the 4EGI‐inhibitor (4EGI‐1, 2.5 *µ* m) that blocks the interaction between eIF4G and eIF4E^[^ ^14b^ ^]^ reduced the levels of YAP protein and restored the budding phenotype and crypt formation (Figure 3J,K and Figure S13G, Supporting Information) associated with control organoids. Therefore, all these data are consistent with a model in which the absence of ECSIT in epithelial cells leads to enhanced levels of eIF4F that promotes YAP translation and transforms intestinal cells to YAP‐overexpression stem “‐like” cells.

### 2.5 The Reprograming to Amino Acid–Based Metabolism in ECSIT‐Deficient Intestine Activates TET Demethylases to Reduce Methylation of Genes Encoding eIF4A1 and eIF4G2

**Figure 4:**
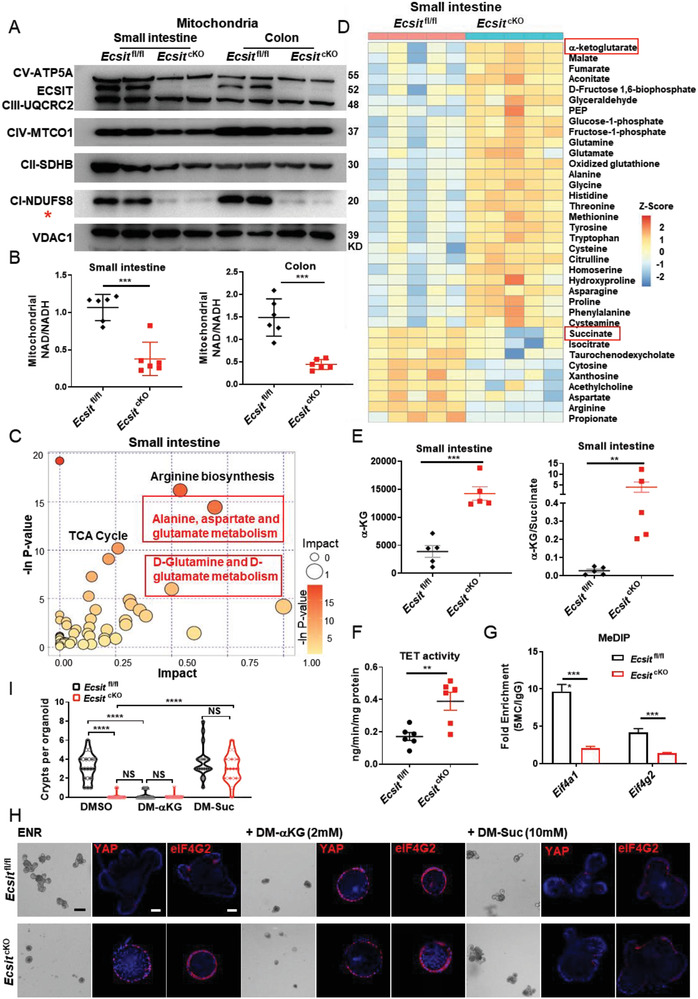
Metabolic reprogramming in ECSIT‐deficient intestine reduces DNA methylation of eIF4F. A) Immunoblotting analysis of ETC complex expression of mitochondria in indicated intestinal epithelium from *Ecsit* ^fl/fl^ and *Ecsit* ^cKO^ mice, red star indicates mitochondrial complex I. B) Mitochondrial NAD^+^/NADH ratio in indicated intestinal epithelium (*n* = 6). Levels were normalized to total protein amount. C) Pathway enrichment analysis of changed metabolites between small intestinal epithelium of *Ecsit* ^fl/fl^ and *Ecsit* ^cKO^ mice (*n* = 5). D) The heatmap displays the relative abundance of significantly changed metabolites in small intestinal epithelium of *Ecsit* ^fl/fl^ and *Ecsit* ^cKO^ mice by liquid chromatography‐tandem mass spectrometry (LC‐MS/MS)‐based metabolomics (*n* = 5 biologically independent experiments). E) LC‐MS/MS quantification of *α*‐KG (left) and the ratio of *α*‐KG/succinate ratio (right), (*n* = 5). F) TET activity of small intestinal epithelium (*n* = 6). G) MeDIP‐qPCR of Eif4a1 and Eif4g2 (*n* = 6). H) Bright‐field imaging and immunofluorescent staining of YAP and eIF4G2 of indicated organoids. Nuclei were labeled with DAPI (blue). Scale bars, 100 µm (BF); 20 µm (IF). I) Quantification of crypts per organoid after DM‐*α*KG or DM‐Suc treatment (*n* = 20 in each group). (A,H) Data are representative of three independent experiments. Error bars show mean ± SEM. **P ≤ 0.01, ***P ≤ 0.001, ****P ≤ 0.0001, NS, not significant. Two‐tailed unpaired student's *t*‐test.

Next, we further explored the possible mechanism by which ECSIT controls the levels of eIF4F expression. The proteomics data showed that GO and KEGG pathways related to mitochondrial oxidative phosphorylation and tricarboxylic acid (TCA) cycle were down‐regulated in *Ecsit* ^cKO^ intestine and instead alanine, aspartate, and glutamate metabolic pathways were enriched in cKO intestine (Figure S12F,G, Supporting Information). Moreover, ECSIT has been suggested as a factor for mitochondrial complex I assembly.^[^ ^8^ ^]^ Immunoblotting analysis revealed that the level of the complex I factor NDUFS8 was strikingly decreased in mitochondria from *Ecsit* ^cKO^ intestine accompanied with a modest change in levels of other components from complex II, III, IV, and V (**Figure** **4**A). This is consistent with the reported phenotype in the deficiency of the assembly of complex I, including ECSIT loss.^[^ ^9^, ^10^, ^17^ ^]^ In the TCA cycle, mitochondrial complex I oxidizes NADH into NAD, which tightly controls intra‐mitochondrial metabolism. In support of impaired function of complex I in *Ecsit* knockout cells, we confirmed a decreased ratio of mitochondrial NAD:NADH in *Ecsit* ^cKO^ intestine (Figure 4B).

We next performed liquid chromatography‐tandem mass spectrometry (LC‐MS/MS)‐based metabolomics on intestinal epithelium from cKO and control mice to determine the exact effect of ECSIT deficiency on the metabolomes of these cells. Principal component analysis (PCA) showed clear clustering of the metabolic profiles based on two groups in both small intestine and colon (Figure S14A,B, Supporting Information). Consistent with our enrichment analysis of proteomic data, metabolites from the alanine, aspartate, and glutamate metabolic pathways were observed to be significantly abundant in the cKO intestine (Figure 4C,D and Figure S14C,D, Supporting Information). More specially, the downstream product of glutamate metabolism, *α*‐ketoglutarate^[^ ^18^ ^]^ was consistently upregulated whereas succinate levels tended to be reduced, resulting in a higher *α*‐KG:succinate ratio in cKO intestine (Figure 4D,E and Figure S14D–F, Supporting Information). Notably, the “‐like” cells of cKO intestine also showed significantly higher expression of glutamate metabolism genes in scRNA‐seq analysis of small intestine and colon (Figure S15A–C, Supporting Information).

A high *α*‐KG/succinate ratio has been suggested to regulate chromatin modifications, including ten‐eleven translocation (TET)‐dependent DNA demethylation, which contributes to the expression of pluripotency‐associated genes to support embryonic stem cell (ESC) self‐renewal while inhibiting differentiation.^[^ ^19^ ^]^ The TET enzyme activity in intestinal tissue of cKO mice was significantly higher than that from control mice (Figure 4F). Notably, CpG island analysis also showed that eIF4A1 and eIF4G2 promoter regions are rich in large numbers of CG sites (Figure S16A, Supporting Information), suggesting that eIF4A1 and eIF4G2 expression may be tightly regulated by DNA methylation. Consistently, we found strongly decreased DNA methylation levels of genes encoding eIF4A1 and eIF4G2 in cKO intestine (Figure 4G). Thus, we propose that the increased *α*‐KG:succinate ratio in cKO intestine leads to higher TET enzyme activity, which in turn reduces the DNA methylation status of the genes encoding for eIF4A1 and eIF4G2.

Accordingly, we found that the addition of exogenous *α*‐KG (dimethyl *α*‐ketoglutarate, DM‐*α*KG) to control organoids, was sufficient to promote high levels of eIF4A1, eIF4G2, and YAP proteins and the conversion of control organoids to a non‐budding regenerative‐state phenotype that phenocopies organoids derived from *Ecsit* ^cKO^ intestine. In contrast, the addition of succinate (dimethyl succinate, DM‐Suc) to cKO organoids strongly decreased the levels of eIF4F and YAP proteins, and reverted the phenotype of organoids to one resembling bud‐forming organoids (Figure 4H,I and Figure S16B,C, Supporting Information). Therefore, all these data suggest that loss of ECSIT in the intestine leads to metabolic reprogramming in favor of amino acid metabolism. This results in a high *α*‐KG:succinate ratio that induces DNA demethylation of the genes encoding eIF4F components, thereby increasing their expression and translation of YAP culminating in transformation of intestinal cells to an early proliferative stem “‐like” phenotype.

### 2.6 ECSIT Loss Potentiates Intestinal Tumorigenesis

**Figure 5:**
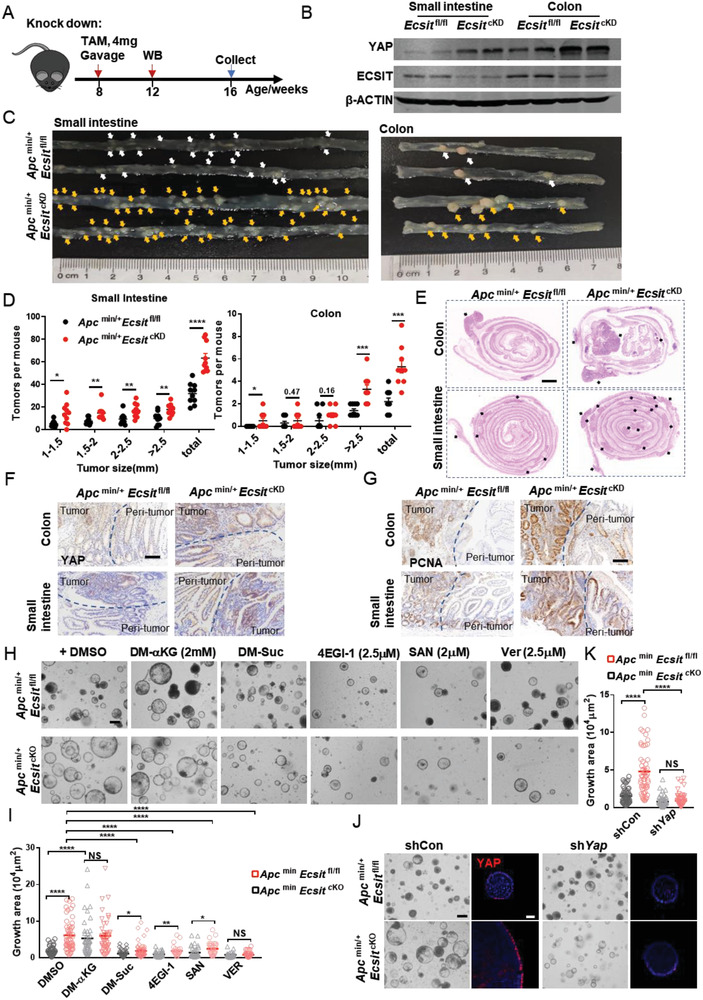
ECSIT loss potentiates intestinal tumorigenesis in *Apc* ^min/+^ mice (A) Schematic of tamoxifen treatment strategy for *Apc* ^min/+^ *Ecsit* ^fl/fl^ and *Apc* ^min/+^ *Ecsit* ^fl/fl^ *Villin‐Cre ER* ^T2^ (termed Apc^min/+^ *Ecsit* ^cKD^). Mice were gavaged with tamoxifen (4 mg) twice every alternate day and euthanized 2 months after the last administration. B) Immunoblotting analysis of ECSIT, YAP, and *β*‐ACTIN (loading control) in intestinal epithelium from indicated mice 2 weeks after the last tamoxifen administration. C–E) Macroscopic image (C), size distribution of tumors (D, *n* = 10) from indicated mice 2 months after last tamoxifen administration, and H&E images (E) of indicated intestine. Scale bar, 1000 µm. F,G) IHC staining for YAP (F) and PCNA (G) of indicated intestine. Scale bars, 100 µm. H) Bright‐field image of tumor organoids treated with indicated metabolites or inhibitors at 72 h from indicated mice. Scale bar, 100 µm. I) Quantification of growth area at 72 h (*n* = 56, 51, 45, 41, 43, 53, 40, 24, 39, 28, 28, and 36 in group from left to right). J) Bright‐field image and Immunofluorescent staining of YAP of shYap tumor organoids at 72 h from indicated mice. Scale bars, 100 µm (BF); 20 µm (IF). K) Quantification of growth area of shYap tumor organoids at 72 h from indicated mice (*n* = 60, 52, 69, and 60 in group from left to right). (B,C,E–H,J) Data are representative of three independent experiments. Error bars show mean ± SEM. *P ≤ 0.05, **P ≤ 0.01, ***P ≤ 0.001, ****P ≤ 0.0001, NS, not significant. Two‐tailed unpaired student's *t*‐test.

The above studies strongly suggest that ECSIT plays crucial roles in maintaining optimal intestinal differentiation homeostasis with the loss of ECSIT being associated with a proliferative stem “‐like” phenotype. Additionally, the occurrence of intestinal tumors is known to be closely related to the imbalance in intestinal homeostasis.^[^ ^2^ ^]^ Next, we explored the possible role of ECSIT in protecting against intestinal tumorigenesis. We initially performed immunohistochemical (IHC) analysis of ECSIT expression in intestines of *Apc* ^min/+^ mice (which are heterozygous for the gene encoding adenomatous polyposis coli [*Apc*] and develop multiple intestinal neoplasia).^[^ ^20^ ^]^ We demonstrated that ECSIT expression was significantly down‐regulated in tumor tissue compared to their unaffected counterpart peri‐tumor tissue (Figure S16D, Supporting Information). To directly explore the role of ECSIT in intestinal tumor development, we constructed *Apc* ^min/+^ *Ecsit* ^fl/fl^ *Vi* *ll* *in‐Cre‐ER* ^T2^ mice and compared with control *Apc* ^min/+^ *Ecsit* ^fl/fl^ mice. Given that deletion of the ECSIT gene in intestine induced low survival rates in mice, we employed an inducible and conditional knockdown model that allowed for an exploration of the role of ECSIT in intestinal tumor development over a long period of time. To this end we gavaged *Apc* ^min/+^ *Ecsit* ^fl/fl^ *Vi* *ll* *in‐Cre‐ER* ^T2^ mice with tamoxifen (4 mg) twice every alternate day to conditionally knock down ECSIT (termed *Apc* ^min/+^ *Ecsit* ^cKD^ in the manuscript) in the intestinal epithelium and this avoided the lethal phenotype manifested by complete ECSIT deletion (**Figure** **5**A,B). The knockdown of *Ecsit* in this model facilitated mouse survival and allowed us to observe the effects of ECSIT deficiency on tumorigenesis. In this knockout model, small intestine and colon showed the expected decrease in levels of ECSIT and enhancement in levels of YAP protein (Figure 5B). The intestinal tumors in *Ecsit* knockdown *Apc* ^min/+^ mice were larger in size and occurred more frequently compared with those in control mice (Figure 5C,D), suggesting that *Ecsit* knockdown promotes tumor formation. H&E staining further confirmed that there were significantly more polyps in the intestines of Ecsit knockdown mice (Figure 5E). Accordingly, the intestines of *Ecsit* knockdown *Apc* ^min/+^ mice exhibited the phenotype of higher regeneration and proliferative rates, as indicated by increased staining for YAP (Figure 5F) and proliferating cell nuclear antigen (PCNA) (Figure 5G).

Next, we cultured tumor organoids in medium without Noggin and R‐Spodin‐1, according to the previously reported method for tumor organoids from *Apc* ^min/+^ mice.^[^ ^21^ ^]^ In this condition, tumor organoids will not undergo differentiation and thus we could compare the expansion of tumor organoids in groups. Untreated *Apc* ^min/+^ mice were euthanized, and the tumors were separated for tumoral organoid culture. Deletion of ECSIT in organoids was then induced by the addition of 4OHT to the culture medium. We found that *Apc* ^min/+^ *Ecsit* ^cKO^ organoids were much larger than *Apc* ^min/+^ *Ecsit* ^fl/fl^ organoids and showed higher YAP expression (Figure 5H,I and Figure S16E,F, Supporting Information). Moreover, the knockdown of Yap reduced the larger *Apc* ^min/+^ *Ecsit* ^cKO^ organoids to sizes comparable to organoids from control tumors (Figure 5J,K), which further suggests that loss of ECSIT does indeed potentiate intestinal tumorigenesis via oncogenic YAP. Next, we evaluated whether the increased *α*‐KG:succinate ratio from ECSIT deficiency provides a mechanistic basis for the increased tumorigenesis observed in *Ecsit* knockdown mice. Exogenous *α*‐KG converted smaller tumor *Apc* ^min/+^ *Ecsit* ^fl/fl^ organoids to larger size and higher YAP expression like *Apc* ^min/+^ *Ecsit* ^cKO^ organoids. Conversely, succinate converted the larger *Apc* ^min/+^ *Ecsit* ^cKO^ organoids to the control tumor organoid size and YAP protein level. Additionally, YAP inhibitor (Verteporfin [Ver], 2.5 *µ* m) and eIF4F inhibitors (4EGI‐1, 2.5 *µ* m and SAN, 2 *µ* m) significantly reduced the size and YAP expression of *Apc* ^min/+^ *Ecsit* ^cKO^ tumor organoids (Figure 5H,I and Figure S16E,F, Supporting Information) illustrating for the role of the eIF4F‐YAP axis in driving the pro‐tumorigenic phenotype resulting from ECSIT deficiency.

**Figure 6:**
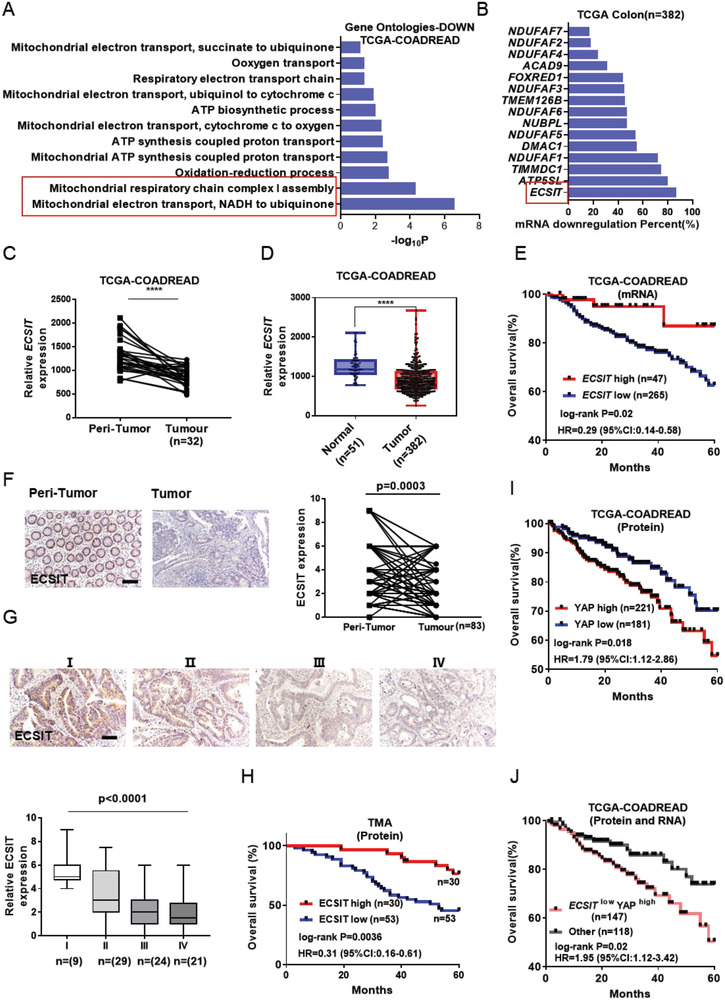
Low expression of ECSIT is associated with tumorigenesis and poor outcome. A) GO enrichment analysis of down‐regulated gene between tumor tissues and paired normal tissues. Histogram showing the enriched terms related to mitochondria. TCGA database (COADREAD: colon adenocarcinoma (COAD) and rectum adenocarcinoma (READ)) was downloaded from http://firebrowse.org/. B) Down‐regulation frequency of mitochondrial complex I assembly factors in tumor tissues. C) Expression of ECSIT between tumor tissue and paired normal tissue (*n* = 32) based on TCGA database. D) Expression of ECSIT between tumor tissue (*n* = 382) and control tissue (*n* = 51) based on TCGA database. E) Kaplan–Meier plot of overall survival of patients based on ECSIT gene expression levels using TCGA data. F) Representative IHC staining (right) and quantification (left) of ECSIT expression between tumor and peri‐tumor from human CRC tissue microarray (TMA). Scale bar, 100 µm. G) IHC staining of human CRC sample microarray (top) and boxed plot of ECSIT expression assessed by blinded IHC analyses of TMA at different clinical stages (bottom). Scale bar, 100 µm. H) Kaplan–Meier plot of overall survival of patients based on ECSIT protein expression levels using IHC data of TMA. I) Kaplan–Meier plot of overall survival of patients based on YAP protein expression levels using TCGA data. J) Kaplan–Meier plot of overall survival of patients based on ECSIT mRNA and YAP protein expression levels using TCGA data. The patients were grouped by the expression of ECSIT mRNA and YAP protein. The data of patients with low ECSIT mRNA and high YAP protein level (ECSIT^low^ YAP^high^) were analyzed to explore the correlation of ECSIT^low^ YAP^high^ expression with prognosis. ****P ≤ 0.0001. Two‐tailed unpaired student's *t*‐test for (D). Two‐tailed paired student's *t*‐test for (C) and (F). One‐way ANOVA test for (G). Log‐rank test for (E), (H), (I) and (J).

Next, we explored the importance of ECSIT in the control of intestinal carcinogenesis in a human context. To this end, we examined the gene expression profiles of CRC clinical samples from TCGA database (COADREAD: colon adenocarcinoma (COAD) and rectum adenocarcinoma (READ)) by GO enrichment analysis. Interestingly, ECSIT was the most significantly down‐regulated factor among the most enriched down‐regulated mitochondrial GO in CRC samples compared with paracancerous tissues (**Figure** **6**A,B). ECSIT was significantly down‐regulated in tumor versus corresponding peri‐tumor tissue (Figure 6C), and in tumor versus control tissue (Figure 6D). The expression of ECSIT was positively correlated with overall survival in COADREAD patients (Figure 6E). IHC of human CRC sample microarrays also indicated that the levels of ECSIT protein were significantly decreased in tumor tissue (Figure 6F), inversely linked to the stages of cancer progression (Figure 6G) and positively associated with survival rate of patients (Figure 6H). Notably, the expression of YAP at the protein level was negatively correlated with the survival of COADREAD patients (Figure 6I), and the prognosis of patients was negatively correlated with ECSIT^low^ YAP^high^ expression (Figure 6J), further supporting our model that ECSIT is a regulator of intestinal tumorigenesis by controlling the protein expression of YAP. Moreover, TCGA data showed low methylation levels and high gene expression of eIF4A1 in tumor tissue compared to control or paired tissues (Figure S16G–J, Supporting Information). Additionally, the expression of ECSIT was negatively related to YAP program genes but not significantly correlated with known CRC markers such as KRAS and TP53 by correlation analysis with TCGA clinical data (Figure S17A,B, Supporting Information), indicating that ECSIT may have the potential to be used as an independent prognostic biomarker for CRC. Collectively, these data suggest a critical role for ECSIT as a regulator of intestinal tumorigenesis.

## 3 Discussion

As a multi‐functional protein, ECSIT plays several physiological roles, including an indispensable role in early embryonic development,^[^ ^5^ ^]^ as a positive regulator of the NF‐*κ*B pathway and inflammation,^[^ ^6^ ^]^ as an enhancer of mROS production and bactericidal activity,^[^ ^7^ ^]^ and as an important factor in complex I assembly for cardioprotection.^[^ ^9^ ^]^ Beyond these findings, in this study we identified a new role for ECSIT: it is a critical regulator for intestinal homeostasis and provides protection against tumorigenesis. Therefore, this finding expands our knowledge about ECSIT.

The intestinal epithelium contains the most diverse cell types of epithelial tissue in the body, whose development and homeostasis must be precisely regulated. However, the crucial factors involved in this process remain unclear. In this study, we characterized how ECSIT controls intestinal differentiation homeostasis and tumorigenesis through the regulation of YAP protein translation. YAP has been recognized as an oncogene in a variety of tumors for its role in regulating stemness and proliferation.^[^ ^5^ ^]^ Nevertheless, the role of YAP in the initiation and progression of CRC has been debated,^[^ ^4^, ^6^, ^7^, ^22^ ^]^ due to which its clinical relevance as a therapeutic target remains inconclusive. Here we indicate the involvement of YAP as an oncogenic protein in CRC as evidenced by the reduced size of tumor organoids after YAP inhibition (Figure 5H–K and Figure S16E,F, Supporting Information) consistent with the function of YAP in driving the pro‐tumorigenic phenotype in CRC. We propose that the loss of ECSIT leads to metabolic reprogramming in favor of amino acid metabolism accompanied by a high proportion of *α*KG to succinate, which results in demethylation of genes encoding the eIF4F pathway and an increase in their expression, which further promotes initiation of YAP translation. This metabolic reprogramming transforms intestinal cells to early proliferative stem “‐like” cell type marked by high YAP protein, thus culminating in intestinal homeostasis imbalance and tumorigenesis (Figure S17D, Supporting Information).

While the mechanism underlying a shift to glutamate metabolism in response to the loss of ECSIT is unknown, it likely represents a metabolic compensatory mechanism when TCA oxidative phosphorylation is impaired. Interestingly, intestinal stem cell proliferation is especially dependent on glutamine,^[^ ^23^ ^]^ but excessive glutamine metabolism has been documented as being crucial for tumorigenesis, including CRC.^[^ ^24^ ^]^ Our data in conjunction with these previous studies, reiterate that glutamine metabolism may represent a valuable pharmacological target to exploit in CRC therapy. Accumulation of *α*‐KG due to enhanced glutamine metabolism in *Ecsit* ^cKO^ intestine is likely to be beneficial for cancer cells by promoting proliferation through mammalian target of rapamycin1 signaling.^[^ ^25^ ^]^ Moreover, a high *α*‐KG:succinate ratio has been suggested to promote the activity of DNA demethylases and regulate chromatin modification in an unusual “open” structure, which is associated with hyper‐transcription and supports ESC self‐renewal.^[^ ^19^ ^]^ Consistently, in our studies we revealed that a high level of *α*‐KG:succinate activates TET demethylases to reduce DNA methylation of eIF4A1 and eIF4G2, which in turn upregulates the eIF4F pathway to promote initiation of YAP translation. Moreover, YAP is critical in inducing stem cell proliferation during the regeneration phase of intestinal injury,^[^ ^3^, ^4^, ^26^ ^]^ and YAP induces the expression of many progenitor‐specific genes.^[^ ^27^ ^]^ YAP expression and activation must be tightly controlled and transient, otherwise it will result in an inability to reacquire homeostasis upon tissue damage, thereby resulting in the development of cancer.^[^ ^22^, ^28^ ^]^ Here we reveal a critical mechanism underlying intestinal tumorigenesis that is driven by ECSIT loss to enhance the levels of translation of YAP protein in an eIF4F‐mediated manner. Thus, our results suggest that targeting eIF4F complex could be an optional strategy in the treatment of colon cancer. Furthermore, our study demonstrated that glutamate metabolism enhanced YAP protein expression, and intriguingly other reports show that activation of YAP signaling can conversely promote glutamate metabolism via several different mechanisms.^[^ ^29^ ^]^ It is very likely that an increase in eIF4A1–YAP axis and glutamate metabolism forms a positive feedback loop system.

ECSIT is required for stabilization of complex I assembly^[^ ^17b^ ^]^ and its absence results in impaired complex I assembly, accumulation of intermediates, and subsequently mitochondrial dysfunction.^[^ ^8^, ^9^, ^10^ ^]^ Moreover, ECSIT deficiency leads to decreased levels of many mitochondrial subunits,^[^ ^9^ ^]^ and this mirrors the phenotype of NDUFA13 deficient cells.^[^ ^30^ ^]^ Notably, the mutation ratio of the *Ecsit* gene allele is not high in patients with CRC (Figure S17C, Supporting Information), and this may reflect that loss‐of‐function mutation of *Ecsit* is incompatible with life. However, a previous study has indicated that ECSIT decreases with age,^[^ ^31^ ^]^ consistent with that, the aging process is heavily implicated in intestinal dysfunction.^[^ ^32^ ^]^ Additionally, our findings may have pathophysiological relevance to the Warburg effect in which cancer cells are reprogrammed to undertake glycolysis even in the presence of oxygen. Some studies suggest that the Warburg effect may result from mutations in complex I resulting in intensification of the Warburg effect.^[^ ^3^, ^33^ ^]^ Thus, the loss of ECSIT may be an important contributor to the Warburg effect in tumor cells.

We also performed combined analysis of our proteomic and transcriptomic data and found 450 molecules unchanged at the transcriptional level but upregulated at the protein level in ECSIT deficient intestine. We compared these 450 molecules with the 284 previously reported genes that rely on eIF4A for translation.^[^ ^34^ ^]^ There were only eight overlapping molecules, including YAP, PTER, PTPN11, ALKBH3, RCBTB1, CUL1, UBE2H, and WNK1 (Figure S18A, Supporting Information). These findings suggest that the abnormal protein level of many molecules caused by ECSIT loss does not depend on eIF4A‐mediated translation, but other translational mechanisms. However, notably, YAP is one of the eight overlapping molecules, further suggesting the enhanced YAP protein levels in ECSIT‐deficient intestine are related to eIF4A‐mediated translational regulation. Thus, this analysis highlights an important role for the eIF4F–YAP axis in driving the abnormal phenotype resulting from ECSIT deficiency. Additionally, we found 285 molecules upregulated at both transcriptional and protein levels in ECSIT‐deficient intestine, and we analyzed these 285 molecules for overlap with 1860 reported genes down‐regulated after Tet‐TKD (triple Knockdown of all three Tet enzymes).^[^ ^35^ ^]^ There were only 72 overlapping molecules, including eIF4A1, eIF4G2, and eIF4G3 (Figure S18B, Supporting Information), suggesting that the expression of major components of the eIF4F complex was regulated by TET demethylase after acquiring ECSIT deficiency.

## 4 Conclusion

In summary, our study addresses the important scientific question of how YAP protein expression is regulated early in intestinal differentiation and describes the critical physiological role of the multi‐functional protein ECSIT in intestinal homeostasis. In addition, the study provides a molecular framework that defines the importance of ECSIT in regulating YAP protein translation to control intestinal tumorigenesis.

## 5 Experimental Section

### Mouse Breeding and Genotyping

All mice belonged to the species *Mus musculus* (C57BL/6). *Ecsit* floxed conditional knockout mice were generated by CRISPR/Cas9 based approach. Briefly, two sgRNAs were designed by CRISPR design tool (http://crispr.mit.edu) to target either a region upstream or downstream of the exon 4, and then were screened for on‐target activity using a Universal CRISPR Activity Assay (Biocytogen Inc, Beijing, China). To minimize random integrations, a circular donor vector was employed. The gene targeting vector containing 5′ homologous arm, target fragment (exon4), 3′ homologous arm was used as a template to repair the DSBs generated by *Cas9*/sgRNA. The two loxp sites were precisely inserted in both sides of target fragment of the *Ecsit* gene. T7 promoter sequence was added to the *Cas9* or sgRNA template by PCR amplification in vitro. *Cas9* mRNA, targeting vector and sgRNAs were co‐injected into the cytoplasm of one‐cell stage fertilized C57BL/6 eggs. The injected zygotes were transferred into oviducts of Kunming pseudopregnant females to generate F0 mice. F0 mice with expected genotype confirmed by tail genomic DNA PCR and sequencing were mated with C57BL/6 mice to establish germline‐transmitted F1 heterozygous mice. F1 heterozygous mice were genotyped by tail genomic PCR, southern blotting and DNA sequencing. The *Ecsit* knockin floxed mice were generated with CRISPR/Cas‐mediated genome engineering by Cyagen Biosciences (Guangzhou, China) Inc. In brief, the gRNA to mouse ROSA26 gene, the donor vector containing “CAG promoter‐loxP‐3*SV40 pA‐loxPKozak‐Mouse *Ecsit* CDS‐3xFLAG‐rBG pA” cassette, and *Cas9* mRNA were co‐injected into fertilized mouse eggs to generate targeted conditional knockin offspring. F0 founder animals were identified by PCR followed by sequence analysis, which were bred to wildtype mice to test germline transmission and F1 animal generation. *Ecsit*‐floxed mice were crossed with *Villin‐Cre* mice (*Villin‐Cre*; the Jackson laboratory) to generate IEC‐conditional *Ecsit* knockout mice (*Ecsit* ^fl/fl^ *Villin‐Cre*). *Ecsit*‐floxed mice were also crossed with *Villin‐Cre ER* ^T2^ (kindly provided by Dr. Sylvie Robine and Dr. Yeguang Chen) or *Lgr5‐Cre ER* ^T2^ (kindly provided by Dr. Jun Qin), and then i.p injected with 3 mg tamoxifen (T5648; Sigma‐Aldrich) dissolved in 300 µL corn oil (C8267; Sigma‐Aldrich) for five consecutive days to induce the expression of Cre recombinase to achieve conditional deletion of *Ecsit* in the intestinal epithelium (*Ecsit* ^fl/fl^ *Villin‐Cre ER* ^T2^, termed *Ecsit* ^cKO^) or in Lgr5^+^ stem cell (*Ecsit* ^fl/fl^ *Lgr5‐CreER* ^T2^‐GFP), respectively at a specific time (6–8 weeks). The mice were euthanized 17 days after the first injection for phenotype analysis. The *Ecsit* knockin floxed mice were crossed with *Ecsit* ^fl/fl^ *Villin‐Cre ER* ^T2^ to rescue the expression of *Ecsit* in ECSIT deficient mice. For spontaneous bowel cancer model, *Ecsit* ^fl/fl^ *Vil* *l* *in‐Cre‐ER* ^T2^ *Apc* ^min/+^ mice (*Apc* ^min/+^ mice were kindly provided by Dr. Jun Qin) was constructed, and the *Ecsit* ^fl/fl^ *Apc* ^min/+^ mice were regarded as control. Mice were gavaged with tamoxifen (4 mg) twice every alternate day to conditionally knock down ECSIT in intestinal epithelium and the mice were euthanized 2 months after the last administration. Mice were housed in conventional cages in an animal room at constant temperature (19–23 °C) and humidity (55 ± 10%) under a 12 h light–dark cycle and were allowed access to standard diet and water ad libitum. All animal experiments were conducted in accordance with the procedure approved by the Ethical Review Committee for Laboratory Animal Welfare of Nanjing Medical University (Approval number: IACUC‐1806012‐1).

### Isolation of Whole Epithelium

The method for isolation of whole epithelium cell for single cell RNA‐seq was based on a previously published method.^[^ ^12^ ^]^ Briefly, the mouse small intestines or colons were flushed with ice‐cold PBS, cut longitudinally into roughly 2 mm‐long pieces, rinsed with ice‐cold PBS, and incubated in 20 mm EDTA at 4 °C for 90 min with continuous shaking. The fraction was washed twice with PBS, and centrifuged at 300 × *g* for 3 min. For single cell RNA‐seq, cells were dissociated with 1× Trypsin‐EDTA solution (Sigma) for 10 min at 37 °C. The single‐cell suspension was then passed through a 70 µm filter. After washing with PBS containing 0.04% BSA, the cell pellets were re‐suspended in PBS containing 0.04% BSA and re‐filtered through a 35 µm cell strainer. Dissociated single cells were then stained with AO/PI for viability assessment using Countstar Fluorescence Cell Analyzer. The single‐cell suspension was further enriched using a MACS dead cell removal kit (Miltenyi Biotec).

### Single‐Cell RNA Sequencing

The scRNA‐Seq libraries were generated using the 10X Genomics Chromium Controller Instrument and Chromium Single Cell 3′ V3 Reagent Kits (10X Genomics, Pleasanton, CA). Briefly, cells were concentrated to 1000 cells per µL and ≈8000 cells were loaded into each channel to generate single‐cell gel bead‐in‐emulsions (GEMs), which results in expected mRNA barcoding of 6500 single‐cells for each sample. After the RT step, GEMs were broken and barcoded‐cDNA was purified and amplified. The amplified barcoded cDNA was fragmented, A‐tailed, ligated with adaptors and index PCR amplified. The final libraries were quantified using the Qubit High Sensitivity DNA assay (Thermo Fisher Scientific) and the size distribution of the libraries were determined using a High Sensitivity DNA chip on a Bioanalyzer 2200 (Agilent). All libraries were sequenced using an Illumina sequencer (Illumina, San Diego, CA) on a 150 bp paired‐end run.

### Analysis of scRNA‐Seq Data

scRNA‐seq data analysis was performed by NovelBio Bio‐Pharm Technology Co., Ltd using NovelBrain Cloud Analysis Platform (www.novelbrain.com). Fastp was applied with default parameters filtering the adaptor sequence and removed the low‐quality reads to attain clean data. Then the feature‐barcode matrices were obtained by aligning reads to the mouse genome (mm10 Ensemble: version 92) using CellRanger v3.1.0. The down sample analysis was applied among samples sequenced according to the mapped barcoded reads per cell of each sample and finally achieved the aggregated matrix. Cells that contained over 800 expressed genes and mitochondria UMI rate below 40% passed the cell quality filtering, and mitochondrial genes were removed in the expression table. Seurat package (version: 3.1, https://satijalab.org/seurat/) was used for cell normalization and regression based on the expression table according to the UMI counts of each sample and percent of mitochondria rate to obtain the scaled data. PCA was constructed based on the scaled data with top 2000 high variable genes and top the ten principals were used for tSNE construction. Utilizing graph‐based cluster method, the unsupervised cell cluster result was acquired based on the PCA top ten principals and the marker genes were calculated by FindAllMarkers function with wilcox rank sum test algorithm under the following criteria: 1) ln fold change > 0.25; 2) *P* value < 0.05; 3) min.pct > 0.1. In order to identify the cell type detailed, the clusters of same cell type were selected for re‐tSNE analysis, graph‐based clustering, and marker analysis. The Gene ontology analysis with GO database (download from NCBI, Uniprot, and AmiGO) and the pathway analysis with KEGG, Biocarta, and hallmark database were applied for functional annotation. Significance *P‐*value was defined by the Fisher's exact test. The ssGSEA function of GSVA package was applied based on several selected gene sets to obtain the score of each group, then violin plot was used to demonstrate the functional difference between groups; the gene signature used can be found in Table S1, Supporting Information. Raw data files have been uploaded to Gene Expression Omnibus public database (GSE198550).

### Intestinal Organoid Cultures and Imaging

The isolation of intestinal crypts and the culture of organoids was based on previously published methods.^[^ ^15^ ^]^ In brief, the section of the initial part of the intestine was opened lengthwise, cleaned with cold PBS. After removal of villi by scraping with a cold glass slide, the intestine was sliced into small fragments roughly 2 mm in length. The tissue was then incubated in 2.5 mm EDTA/PBS at 4 °C for 30 min with shaking. Crypts were detached from the tissue through vigorous shaking and filtered through a 70 µm filter. Crypts were embedded onto Matrigel (Corning) at 50 µL per well in 24‐well plates. The primary culture medium was advanced Dulbecco's modified Eagle medium/F12 (Gibco) containing GlutaMAX (2 mm, Solarbio), HEPES (10 mm, Solarbio), NAC (1 mm, bryotime), penicillin–streptomycin (100 U mL^−1^ Gibco) and B27 (1:50, Gibco). The ENR medium was supplemented with EGF (50 ng mL^−1^, Peprotech), Noggin (100 ng mL^−1^, Peprotech), and R‐spondin‐1 (500 ng mL^−1^, Peprotech). For *Apc* ^Min/+^ primary mouse tumor organoids, the medium was primary culture medium supplemented with mouse EGF alone based on the previously reported method.^[^ ^21^ ^]^ Knockout induction in the crypt cultures was achieved by incubation with 300 nm 4OHT (Sigma‐Aldrich) after passage for 72 h. Organoids were treated with 2.5 *µ* m 4EGI‐1 (MCE, HY‐19831), 2 *µ* m sanguinarine chloride (MCE, HY‐N0052A), 2.5 *µ* m Verteporfin (CSNpharm, CSN12195), 2 mm DM‐*α*KG (Sigma, 349 631) or 10 mm DM‐Suc (Sigma, W239607), respectively, after passage for 72 h in indicated experiment. 3MA (5 mm) and MG132 (5*µ* m) were added into the medium 4 h before fixation for immunofluorescent staining. For knockdown of YAP, shYap expressing pLKO.1 vector was introduced into ECSIT‐deficient organoids and ECSIT‐deficient *Apc* ^min/+^ tumor organoids by lentiviral infection. Briefly, lentiviral particles were produced by co‐transfection with pLKO‐shYap (5′‐TGAGAACAATGACAACCAATA‐3′) with the packaging plasmid (psPAX2 and pMD2.G) using PolyJet reagent according to the manufacturer's instructions. The culture medium was replaced 12 h after transfection, and the viral supernatant was collected 48 h later and concentrated by overnight centrifugation at 20 000 × *g* at 4 °C. Crypts from normal organoids or shattered fragments from tumor organoids were infected with viral supernatant, embedded in Matrigel 8 h post infection and then cultured in organoid culture medium supplemented with 10 µm Y‐27632 (MCE, HY‐10071) and treated with 4OHT to delete ECSIT. Fixed sample preparation and imaging was based on the previously described method.^[^ ^4d^ ^]^ The plate was centrifuged at 3000 rpm for 10 min in a pre‐cooled centrifuge at 10 °C prior to fixation. Organoids were fixed in 4% PFA in PBS for 45 min at room temperature, permeabilized with 0.5% Triton X‐100 for 1 h (for YAP immunofluorescent staining of intestinal organoid) or unpermeabilized (for other staining), blocked with 5% goat serum and 0.1% Triton X‐100 in PBS for 1 h. Organoids were incubated with primary antibodies diluted in blocking buffer at 4 °C overnight, primary antibodies were anti‐LYZ1 (Abcam, 1:250), rabbit anti‐YAP (CST, 1:400), rabbit anti‐KI67 (Abcam, 1:400), rabbit anti‐eIF4A1 (Bioworld, 1:500), and rabbit anti‐eIF4G2 (CST, 1:500), and then were incubated with the secondary antibodies for 2 h at room temperature. Cell nuclei were stained with 20 µg mL^−1^ DAPI (4′,6‐diamidino‐2‐phenylindole, Invitrogen) in PBS for 10 min. Images were captured using Zeiss LSM 800 confocal microscope and processed using ImageJ 1.53c and AdobePhotoshopCS6 software. For immunofluorescent quantification, the indicated fluorescence intensity of organoids was normalized to the intensity of DAPI.

### RNA‐Seq

RNA isolation, library construction, and sequencing were performed on a MGI2000 (Beijing Genomic Institution, BGI, China). Clean reads were mapped to the mouse genome (GRCm38.p6) by HISAT2. For gene expression analysis, the matched reads were calculated and then normalized to FPKM. Fold changes were calculated for all possible comparisons and a 1.2‐fold cutoff was used to select genes with expression changes. GO pathway enrichment analysis was performed by applying the online tool of DAVID Bioinformatics Resources 6.8 (http://david.ncifcrf.gov), using significantly differentially expressed genes (fold change >1.2, *p* value < 0.05) as target genes. GSEA analysis was performed using software (GSEA v3.0). The sequencing data have been deposited in the NCBI sequence read archive (SRA) database under the NCBI Bioproject PRJNA795031.

### Isolation of Lamina Propria Cells and Flow Cytometry

Small intestine and colon tissues were incubated at 37 °C in PBS containing 2% FBS and 5 mm EDTA for 25 min. The remaining tissue was cut into small pieces and digested in PBS containing 2% FBS, Collagenase IV (0.5 mg mL^−1^; Thermo), and DNase I (10 U mL^−1^; Sigma‐Aldrich) and then incubated at 37 °C for 45 min. Single cell suspensions were stained with anti‐CD45‐Alexa Flour 700 (eBioscience, 30‐F11, 56‐0451‐82, 1:400), anti‐CD4‐APC‐Cy7 (Biolegend, GK1.5, 100 414, 1:400), anti‐CD8a‐ PE (eBioscience, 53–6.7, 12‐0081‐83, 1:400), anti‐NK1.1‐PE‐Cy7 (eBioscience, PK136, 25‐5941‐82, 1:400), anti‐CD19‐APC (eBioscience, 1D3, 17‐0193‐80, 1:400), anti‐CD11b‐FITC (Biolegend, 101 206, M1/70, 1:400), anti‐CD11c‐PE (eBioscience, N418, 12‐0114‐82, 1:400), and FVD eFlour 506 (eBioscience,1:1000) for FACS analysis (Thermo). All flow cytometry analyses were performed on an Attune NxT Flow Cytometer (Thermo Fisher Scientific), and data were analyzed using FlowJo 10 software.

### Histology, Immunofluorescence, and Immunohistochemistry

For histology, the samples were washed, fixed in 4% buffered formaldehyde, and embedded in paraffin. Tissue sections were stained with H&E. Images were acquired with a Nikon 50i inverted microscope. For immunofluorescence (IF) and IHC, freshly isolated tissue was fixed in 4% paraformaldehyde, dehydrated and mounted in paraffin using standard protocols. Material for frozen sections was processed and embedded in OCT (Tissue‐Tek; Sakura Finetek) according to standard protocols. Standard immunohistochemical protocols were performed using the following antibodies: rabbit anti‐ECSIT (Nuvus, 1:400), rabbit anti‐YAP (CST, 1:400), rabbit anti‐MUC2 (Abcam, 1:100), rabbit anti‐ChgA (Nuvus, 1:300), rabbit anti‐DCLK (Abcam, 1:800), rabbit anti‐LYZ1 (Abcam, 1:250), rabbit anti‐SCA1‐Alexa Fluor 647 (Biolegend, 1:400), rabbit anti‐CCND2 (SAB, 1:200), rabbit anti‐KI67 (Abcam, 1:250), rabbit anti‐eIF4A1 (Bioworld, 1:100), rabbit anti‐eIF4G2 (CST, 1:400), mouse anti‐PCNA (Santa Cruz, 1:300), rabbit anti‐OLFM4 (CST, 1:400), rabbit anti‐SOX9 (Sigma, 1:300), and rabbit anti‐GFP tag (Proteintech, 1:150). Secondary antibodies were anti‐rabbit goat antibodies conjugated with Alexa (A488 or A555) from ThermoFisher Scientific. For Tunel staining, Fluorescein (FITC) Tunel Cell Apoptosis Detection Kit (Servicebio) was used. For IHC staining, secondary antibodies were horseradish peroxidase polymer‐conjugated goat anti‐mouse or goat anti‐rabbit (Thermo). Diaminobenzidine chromogen (Boster) was used for detection and haematoxylin was used as a counterstain. Images for HE and IHC were acquired on Nikon DS‐Ri2 using NIS‐Elements F 4.60 software. Images for Immunofluorescence were acquired on Zeiss LSM 800 confocal microscope using ZEN software.

### Tissue Microarrays and IHC Staining

A tissue microarray (TMA) containing 83 paired CRC tissues (Cat No. COC1602) was purchased from the Shanghai Superbiotek Pharmaceutical Technology (Shanghai, China). Antibodies against ECSIT were used for immunohistochemistry staining. Data analysis was performed blindly.

### RNA Extraction and Real Time Quantitative PCR

Total RNA was extracted using TRIzol reagent (Life) and subjected to complementary DNA synthesis. Reverse transcription products of different samples were amplified by StepOnePlus (Applied Biosystems) using AceQ qPCR SYBR Green Master Mix (Vazyme) according to the manufacturer's instructions. Data were analyzed according to the Δ*C*_t_ method. All results were normalized to Hprt quantified in parallel amplification reactions. All primers were purchased from Sangon Biotech. Primers used were Hprt‐F: 5′ GTCCCAGCGTCGTGATTAGC 3′; Hprt‐R: 5′ TGATGGCCTCCCATCTCCT 3′; Yap‐F: 5′ TGAGATCCCTGATGATGTACCAC 3′; Yap‐R: 5′ TGTTGTTGTCTGATCGTTGTGAT 3′; Ly6a‐F: 5′ GAAAGAGCTCAGGGACTGGAGTGTT 3′; Ly6a‐R: 5′ TTAGGAGGGCAGATGGGTAAGCAA 3′; Ccnd2‐F: 5′ GTCCCGACTCCTAAGACCCAT 3′; Ccnd2‐R: 5′ CAGGCTTTGAGACAATCCACAT 3′; Ctgf‐F: 5′ GGGCCTCTTCTGCGATTTC 3′; Ctgf‐R: 5′ ATCCAGGCAAGTGCATTGGTA 3′; Areg‐F: 5′ GCAGATACATCGAGAACCTGGAG 3′; Areg‐R: 5′ CCTTGTCATCCTCGCTGTGAGT 3′; Cyr61‐F: 5′ CTGCGCTAAACAACTCAACGA 3′; and Cyr61‐R: 5′ GCAGATCCCTTTCAGAGCGG 3′.

### Immunoblotting

Homogenates of epithelium were lysed in RIPA solution (Yeasen) supplemented with a protease inhibitor cocktail (Sigma‐Aldrich) at 4 °C for 1 h. The protein from epithelial mitochondria was extracted using the Mitochondrial Isolation Kit (Yeasen) according to the manufacturer's instructions. Nuclear proteins from epithelial cells were extracted using the Nuclear and Cytoplasmic Protein Extraction Kit (Sangon Biotech) according to the manufacturer's instructions. Samples were clarified, denatured with SDS buffer, and boiled for 10 min. Proteins were separated by SDS–polyacrylamide gel electrophoresis and transferred onto nitrocellulose membranes. The membranes were immunoblotted with primary antibodies: rabbit anti‐ECSIT (Novus, 1:1000), rabbit anti‐YAP (CST, 1:1000), rabbitanti‐SCA1 (SAB, 1:1000), rabbit anti‐Histone H3 (Proteintech, 1:2000), rabbit anti‐CCND2 (SAB, 1:1000), rabbit anti‐ATP5A (Bioworld, 1:1000), rabbit anti‐UQCRC2 (Bioworld, 1:1000), rabbit anti‐SDHB (Bioworld, 1:1000), rabbit anti‐MTCO1 (Abcam, 1:1000), or mouse anti‐NDUFS8 (Santa Cruz, 1:1000) and proteins detected with appropriate secondary anti‐rabbit or anti‐mouse antibody conjugated to fluorescence. Immunoreactivity was visualized using the Odyssey Imaging System (LI‐COR Biosciences).

### Metabolomics

Whole epithelium from colon and small intestine were freeze dried for 12 h and weighed. The freeze‐dried tissues were thawed out on the ice, extracted with 800 µL of 80% methanol, vortexed for 1 min. Then the samples were ultrasonicated for 30 min at 4 °C, and let stand for 1 h at −40 °C, vortexed for 30 s, and let stand for 0.5 h at 4 °C, centrifuged at 12 000 × *g* and 4 °C for 15 min. All the supernatants were taken in the centrifuge tube and concentrated to remove organic reagents and water, and then reconstituted in 100 µL 80% methanol, vortex 1 min, centrifuged at 12 000 × *g* for 15 min and the supernatant was transferred to vial for LC‐MS/MS‐based widely targeted metabolomics in the Shanghai Sensichip Infotech Co. (Shanghai, China). The final data were imported into SIMCA‐P software for multivariate statistical analysis. PCA was carried out to explore the differences of the metabolites between the groups. Metabolite abundance between samples can be found in Table S2, Supporting Information. Differential metabolite data were used for pathway enrichment analysis on the MetaboAnalyst 3.0 (http://www.metaboanalyst.ca). *P*‐values <0.05 were considered statistically significant.

### Proteomics

Whole epithelium from colon and small intestine were incubated in lysis buffer (1% DOC, 10 mm TCEP, 40 mm CAA, and 100 mm Tris‐HCl pH 8.8). After heating at 95 °C for 5 min, they were placed on ice for ultrasonic disintegration (3 s on, 3 s off, 30% energy). The mixture was centrifuged at 16 000 × *g* for 10 min at 4 °C and the supernatant was saved as a whole tissue extract. The protein concentration was quantified by Thermo Nanodrop One (Thermo Fisher, USA). Protein (100 µg) was cleaved at the C‐terminus of Arg or Lys residues overnight at 37 °C with 1:50 trypsin (Promega, USA). The next day, the digestion was stopped by adding formic acid at a final concentration of 1%. LC‐MS/MS analysis was performed on an Orbitrap Fusion Tribrid mass spectrometer (Thermo Fisher Scientific) coupled on‐line to a nanoflow LC system (EASY‐nLC 1200, Thermo Fisher Scientific) at the National Center for Protein Sciences (Beijing, China). GO and KEGG pathway enrichment analysis was performed by applying the online tool of DAVID Bioinformatics Resources 6.8 (http://david.ncifcrf.gov). GSEA analysis was performed using software (GSEA v3.0). The mass spectrometry proteomics data had been deposited to the ProteomeXchange Consortium (http://proteomecentral.proteomexchange.org) via the iProX partner repository with the dataset identifier PXD033272.

### TET Activity Assay

Intestinal epithelium nuclear DNA were extracted, and the 5mC Hydroxylase TET Activity/Inhibition Assay Kit (Colorimetric) (Epigentek) was used to assay TET enzymatic activity according to the manufacturer's instructions.

### Sucrose Gradient Polysome Fractionation

Intestinal epithelium lysates were prepared in a buffer containing 100 mm KCl, 0.1% Triton X‐100, 50 mm HEPES, 2 mm MgCl_2_, 10% glycerol, 1 mm DTT, 20 U mL^−1^ Protector RNase Inhibitor (Vazyme), and 1× EDTA free protease inhibitor cocktail (Roche), and kept on ice for 15 min before centrifugation at 10 000 × *g* for 10 min. The supernatant was carefully loaded on 20 to 50% w/v linear density sucrose gradient (Gradient Master, Biocomp, Fredericton, NB, Canada) and centrifuged at 38 000 rpm, for 2.5 h (Beckman Coulter Optima L‐100XP Ultracentrifuge, Brea, CA, USA). The polysome fractions were collected using a piston gradient fractionator (Biocomp). In addition, the total RNA in each tube was isolated and the RNA expression of *Yap* in each fraction was determined by real‐time qPCR. Primers used were Yap‐F: 5′ TTGGAGGCGCTCTTCAATG 3′, Yap‐R: 5′ TCCTGCCATGTTGTTGTCTGA 3′, Gapdh‐F: 5′ GAAGGTCGGTGTGAACGGATTTG 3′, and Gapdh‐R: 5′ ATTTGATGTTAGTGGGGTCTCGCTC 3′.

### Methylated DNA Immunoprecipitation

Methylated DNA immunoprecipitation (MeDIP) analysis was performed according to the manufacturer's instructions (Bersinbio). Total genomic DNA was sonicated to yield DNA fragments between 300 and 700 bp in length. One microgram of fragmented DNA was heat denatured to produce single‐stranded DNA. Immunoprecipitation was performed overnight at 4 °C using 5 µg anti‐5mC antibody (Abcam), with 5 µg of normal rabbit IgG as the negative control. Real‐time qPCR was performed to evaluate the DNA methylation of target gene. Primers used were Eif4a1‐F: 5′ GGCTGTGCTTTATCTCAGGTCTTC 3′, Eif4a1‐R: 5′ TCATATTTACTGCCATCTGCTCCC 3′, Eif4g2‐F: 5′ CAGCCTCATGCTTGCATAGAAGAACC 3′, and Eif4g2‐R: 5′ AAAGCTCCCAGACGCTAAACCG 3′.

### RIP

RIP analysis was performed according to the manufacturer's instructions (Bersinbio). Immunoprecipitation was performed for overnight at 4 °C using eIF4A1 (Bioworld, 1:50) with 5 µg of normal rabbit IgG as the negative control. The RNA was subjected to cDNA synthesis and RT‐qPCR was performed.

### Statistical Analysis

All experiments were performed with at least three replicates. Statistical analysis was performed using GraphPad Prism 9.0 or Origin Pro 8. Flow cytometry data were analyzed by FlowJo V10. All data were presented as the mean ± SEM except for where indicated otherwise. Statistical differences were calculated using two‐tailed Student's *t*‐test between two groups. For a single comparison between two groups, paired Student's *t*‐tests were used. Nonparametric *t*‐test was chosen if the sample size was too small and did not fit the Gaussian distribution. For patient survival analysis, the log‐rank test was used to determine the statistical significance. Correlation analyses were performed using the Pearson correlation method. In all cases, significance was defined as *p* < 0.05, * means *p* < 0.05, ** means *p* < 0.01, *** means *p* < 0.001, **** means *p* < 0.0001. More information about statistical details including sample size (*n*) for each statistical analysis is indicated in the figure legends.

## Conflict of Interest

The authors declare no conflict of interest.

## Author Contributions

Y.J., C.M.M., Y.H., Y.Y., C.Y.M., C.W., L.L., and S.W. designed and performed the experiments, analyzed the data and prepared the figures; B.W. provided the key technique mentoring, research reagents, and mice; B.W. and P.N.M. contributed to the experimental design and edited the manuscript; S.Y. supervised the project; Y.J., C.M.M., P.N.M., and S.Y. prepared the manuscript.

## Supporting information

Supporting Information

Supplemental Table 1

Supplemental Table 2

## Data Availability

Data are available on reasonable request. All data relevant to the study are included in the article or uploaded as Supporting Information. Single cell RNA‐Seq Raw data files have been uploaded to Gene Expression Omnibus public database (GSE198550). The bulk RNA sequencing data have been deposited in the NCBI SRA database under the NCBI Bioproject PRJNA795031. The mass spectrometry proteomics data have been deposited to the ProteomeXchange Consortium (http://proteomecentral.proteomexchange.org) via the iProX partner repository with the dataset identifier PXD033272.
