# Supplementary material for: ECSIT Is a Critical Factor for Controlling Intestinal Homeostasis and Tumorigenesis through Regulating the Translation of YAP Protein
Source: Adv Sci (Weinh). 2023 Jul 6;10(25):2205180. doi: 10.1002/advs.202205180 (PMC10477885; doi:10.1002/advs.202205180)
Supplement: Supplementary file 1 — Supporting Information [file ADVS-10-2205180-s001.pdf]

ADVANCED SCIENCE
Open Access

Supporting Information

for *Adv. Sci.*, DOI 10.1002/advs.202205180

ECSIT Is a Critical Factor for Controlling Intestinal Homeostasis and Tumorigenesis through Regulating the Translation of YAP Protein

Yuying Jiang, Chunmei Ma, Yingchao Hu, Yongbing Yang, Chanyuan Ma, Chunyan Wu, Lu Liu, Shuang Wen, Paul N. Moynagh*, Bingwei Wang* and Shuo Yang**

ECSIT is a Critical Factor for Controlling Intestinal Homeostasis and Tumorigenesis through Regulating the Translation of YAP Protein

Consolidated Supporting Information

Yuying Jiang[1#], Chunmei Ma[1#], Yingchao Hu[1#], Yongbing Yang[1#], Chanyuan Ma[1], Chunyan Wu[1], Lu Liu[1], Shuang Wen[1], Paul N Moynagh[3,4]*, Bingwei Wang[2]* and Shuo Yang[1,5]**

* Correspondence: shuoyang01@njmu.edu.cn (S.Y. Lead contact)

bingweiwang@njucm.edu.cn (B.W.W.)

Paul.Moynagh@mu.ie (P.N.M.)

macm@njmu.edu.cn (C.M.M.)

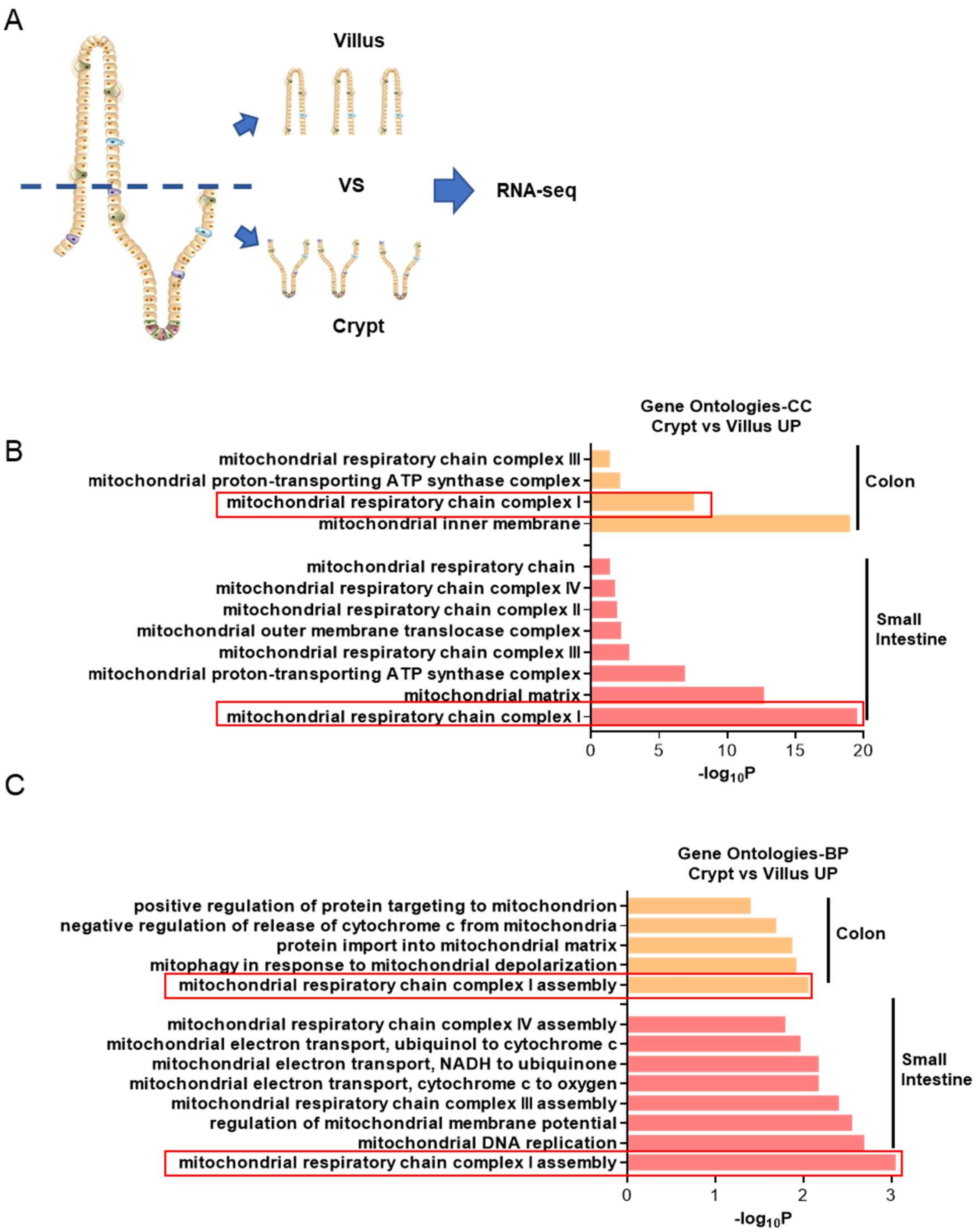
A
Villus
VS
RNA-seq
Crypt
B
Gene Ontologies-CC
Crypt vs Villus UP
mitochondrial respiratory chain complex III
mitochondrial proton-transporting ATP synthase complex
mitochondrial respiratory chain complex I
mitochondrial inner membrane
Colon
mitochondrial respiratory chain
mitochondrial respiratory chain complex IV
mitochondrial respiratory chain complex II
mitochondrial outer membrane translocase complex
mitochondrial respiratory chain complex III
mitochondrial proton-transporting ATP synthase complex
mitochondrial matrix
mitochondrial respiratory chain complex I
Small Intestine
0
5
10
15
20
$-\log_{10}P$
C
Gene Ontologies-BP
Crypt vs Villus UP
positive regulation of protein targeting to mitochondrion
negative regulation of release of cytochrome c from mitochondria
protein import into mitochondrial matrix
mitophagy in response to mitochondrial depolarization
mitochondrial respiratory chain complex I assembly
Colon
mitochondrial respiratory chain complex IV assembly
mitochondrial electron transport, ubiquinol to cytochrome c
mitochondrial electron transport, NADH to ubiquinone
mitochondrial electron transport, cytochrome c to oxygen
mitochondrial respiratory chain complex III assembly
regulation of mitochondrial membrane potential
mitochondrial DNA replication
mitochondrial respiratory chain complex I assembly
Small Intestine
0
1
2
3
$-\log_{10}P$

Figure S1. Mitochondrial complex I assembly is an enriched signature of crypt versus villus

(A) Schematic of RNA-seq strategy.

(B) GO-CC (Cellular Component) enrichment analysis of upregulated gene in intestinal crypt compared to villus.

(C) GO-BP (Biological Process) enrichment analysis of upregulated gene in intestinal crypt compared to villus.

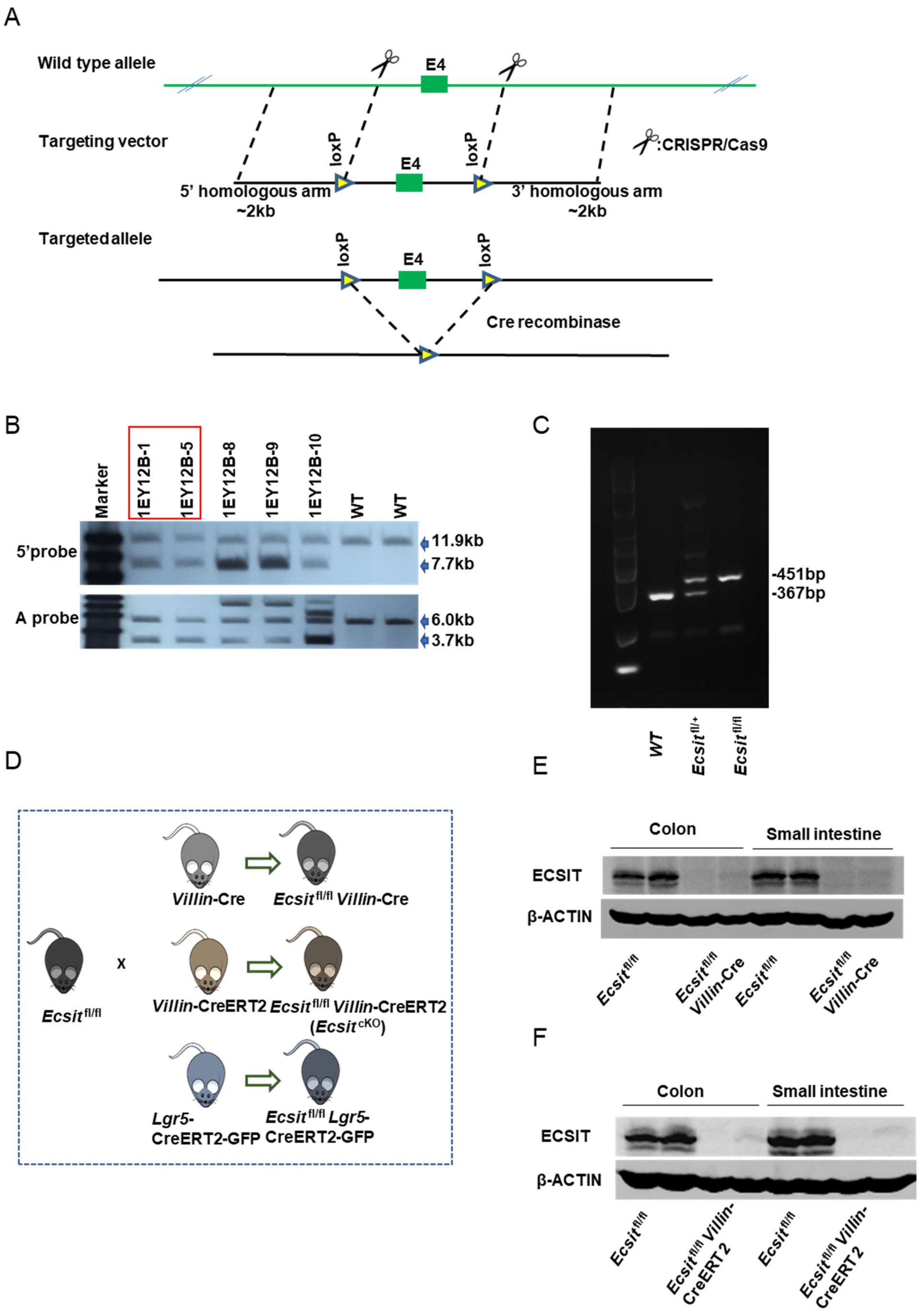

A
Wild type allele
E4
Targeting vector
:CRISPR/Cas9
loxP
E4
loxP
5' homologous arm
~2kb
3' homologous arm
~2kb
Targeted allele
loxP
E4
loxP
Cre recombinase
B
Marker
1EY12B-1
1EY12B-5
1EY12B-8
1EY12B-9
1EY12B-10
WT
WT
5'probe
11.9kb
7.7kb
A probe
6.0kb
3.7kb
C
-451bp
-367bp
WT
Ecsit fl/+
Ecsit fl/fl
D
Villin-Cre
Ecsit fl/fl Villin-Cre
Ecsit fl/fl
x
Villin-CreERT2
Ecsit fl/fl Villin-CreERT2
(Ecsit cKO)
Lgr5-CreERT2-GFP
Ecsit fl/fl Lgr5-CreERT2-GFP
E
Colon
Small intestine
ECSIT
β-ACTIN
Ecsit fl/fl
Ecsit fl/fl Villin-Cre
Ecsit fl/fl
Ecsit fl/fl Villin-Cre
F
Colon
Small intestine
ECSIT
β-ACTIN
Ecsit fl/fl
Ecsit fl/fl Villin-CreERT2
Ecsit fl/fl
Ecsit fl/fl Villin-CreERT2

Figure S2. Generation of mice with conditional intestinal deletion of ECSIT

(A) Targeting vector design for generation of a mouse strain with *Ecsit* exon 4 flanked by loxp sites.

(B) Southern blot analysis of F1 mice, 1EY12B-1 and 1EY12B-5 were chosen.

(C) Representative genotyping image for analysis of *Ecsit* loxp site.

(D) Schematic of mice hybridization strategy.

(E) Immunoblotting analysis of ECSIT and β-ACTIN (loading control) in intestinal epithelium from indicated 2-month-old mice.

(F) Immunoblotting analysis of ECSIT and β-ACTIN (loading control) in intestinal epithelium from indicated mice on day17 after first tamoxifen injection.

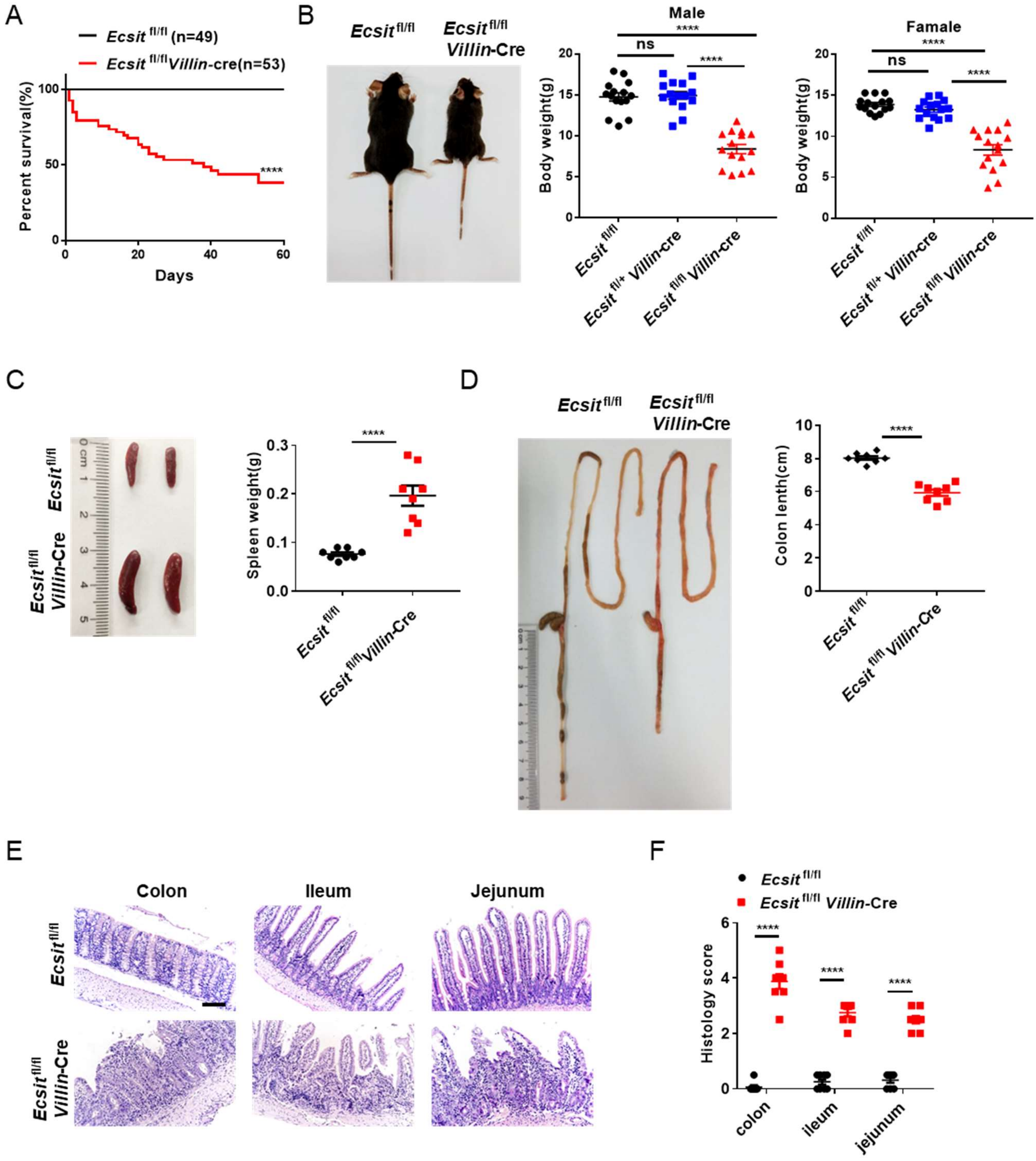
A
Ecsit fl/fl (n=49)
Ecsit fl/fl Villin-cre(n=53)
Percent survival(%)
Days
****
B
Ecsit fl/fl
Ecsit fl/fl Villin-Cre
Male
Famale
ns
****
Body weight(g)
Ecsit fl/fl
Ecsit fl/+ Villin-cre
Ecsit fl/fl Villin-cre
C
Ecsit fl/fl
Ecsit fl/fl Villin-Cre
Spleen weight(g)
****
D
Ecsit fl/fl
Ecsit fl/fl Villin-Cre
Colon lenth(cm)
****
E
Colon
Ileum
Jejunum
Ecsit fl/fl
Ecsit fl/fl Villin-Cre
F
Ecsit fl/fl
Ecsit fl/fl Villin-Cre
Histology score
****
colon
ileum
jejunum

Figure S3. Ablation of ECSIT in intestine manifests spontaneous intestinal inflammation phenotype

(A) Kaplan-Meier plot of overall survival of indicated mice. Log-rank test was used for statistical analysis.

(B) Macroscopic image of indicted 4-week-old mice (left), body weight of indicated 4-week-old male (middle, n=15) and female (right, n=15) mice.

(C) Macroscopic image (left) and weight (right) of spleen (n=8) from 8-week-old mice.

(D) Macroscopic image of intestine (left) and colonic length (right, n=8) from 8-week-old mice.

(E) H&E staining of indicated intestine of 8-week-old mice. Scale bar, 100 μm.

(F) Histology scores of indicated mice in (E), (n=8).

Data are pooled from three independent experiments (B-D and F). Two-tailed unpaired student's *t*-test. Error bars show mean ± SEM. ****$P \leq 0.0001$, NS, not significant.

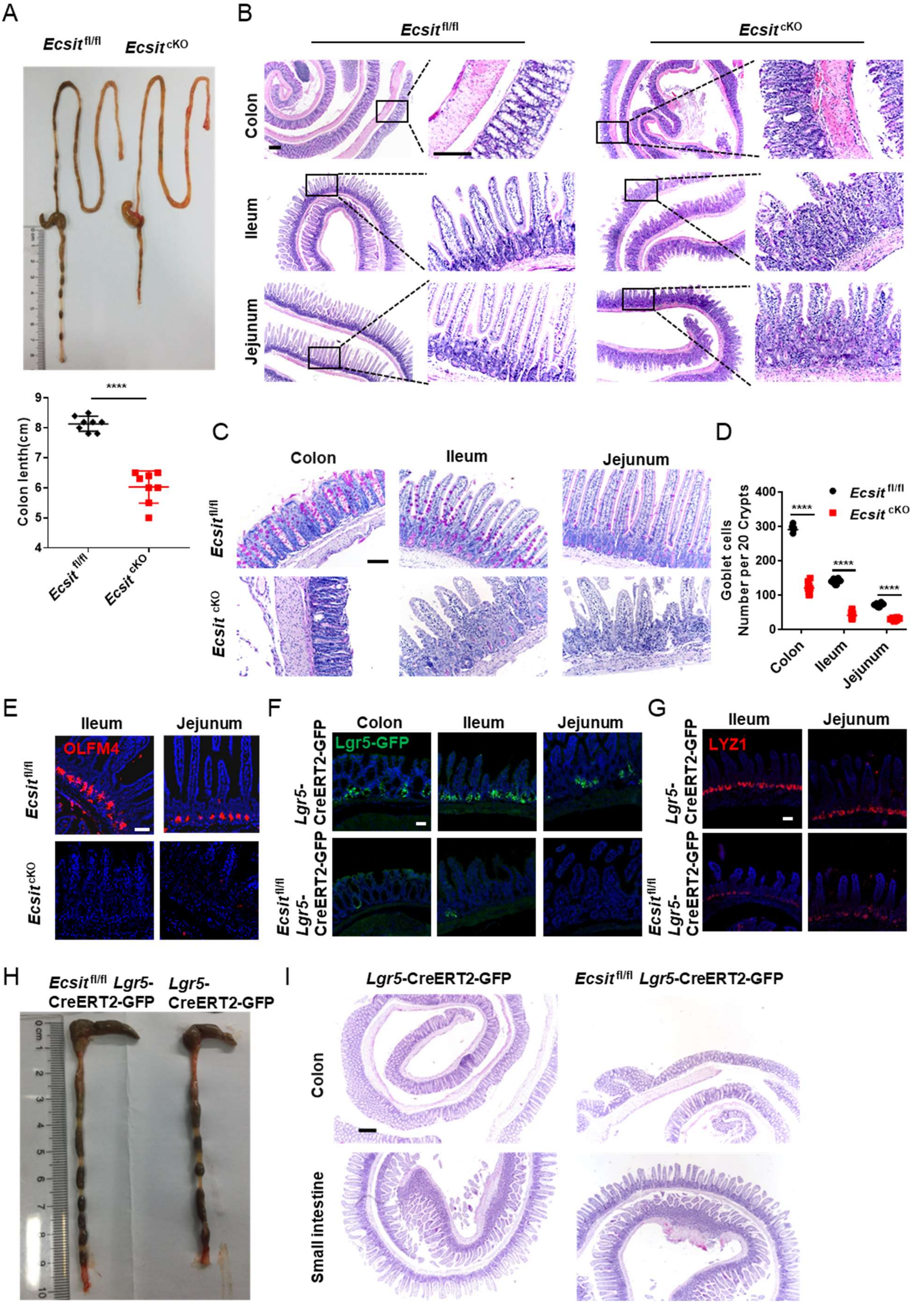

A
Ecsit fl/fl
Ecsit cKO
****
Colon lenth(cm)
B
Colon
Ileum
Jejunum
C
D
Goblet cells Number per 20 Crypts
E
OLFM4
F
Lgr5-CreERT2-GFP
Lgr5-GFP
Ecsit fl/fl Lgr5-CreERT2-GFP
G
LYZ1
H
I
Small intestine

Figure S4. Lack of ECSIT in intestine leads to the dysregulation of intestinal differentiation

(A) Macroscopic image of intestine (left) and colonic length (right, n=8) on day 17 after first tamoxifen injection.

(B) H&E staining of indicated intestine on day 17 after first tamoxifen injection. Scale bars, 100 μm.

(C) PAS staining for goblet cell of intestine on day 17 after first tamoxifen injection. Scale bars, 100 μm.

(D) Quantification of goblet cells in (C) (n=8).

(E) Immunofluorescence staining for OLFM4 of ileum and jejunum from indicated mice. Nuclei were labeled with DAPI (blue). Scale bar, 100 μm.

(F) Fluorescence image of *Lgr5*-CreERT2-GFP and *Ecsit* $^{fl/fl}$ *Lgr5*-CreERT2-GFP mice. GFP positive cells represent the $Lgr5^+$ stem cells. Nuclei were labeled with DAPI (blue). Scale bar, 100 μm.

(G) Immunofluorescence staining for LYZ1 of ileum and jejunum respectively from indicated mice. Nuclei were labeled with DAPI (blue). Scale bar, 100 μm.

(H) Macroscopic image of colon on 2 months after first tamoxifen injection.

(I) H&E staining of indicated intestine on 2 months after first tamoxifen injection. Scale bar, 300 μm.

Data are representative of three independent experiments (B, C, E-G and I). Error bars show mean ± SEM. ****$P \leq 0.0001$. Two-tailed unpaired student's *t*-test.

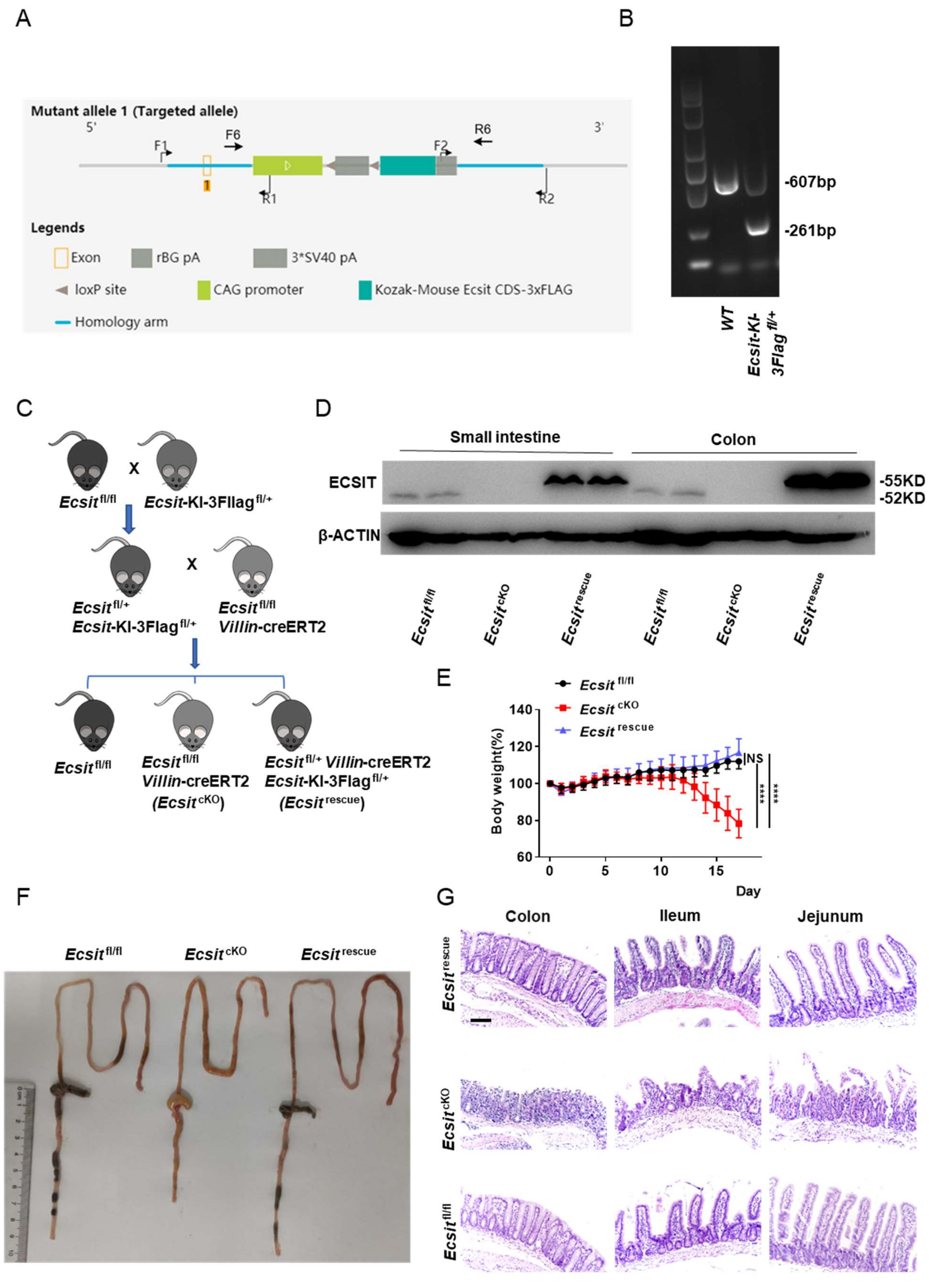

A
Mutant allele 1 (Targeted allele)
5'
3'
F1
F6
F2
R6
R1
R2
1
Legends
Exon
rBG pA
3*SV40 pA
loxP site
CAG promoter
Kozak-Mouse Ecsit CDS-3xFLAG
Homology arm
B
-607bp
-261bp
WT
Ecsit-KI-3Flag fl/+
C
Ecsit fl/fl
X
Ecsit-KI-3Fllag fl/+
Ecsit fl/+
Ecsit-KI-3Flag fl/+
Ecsit fl/fl
Villin-creERT2
Ecsit fl/+ Villin-creERT2
Ecsit-KI-3Flag fl/+
(Ecsit rescue)
(Ecsit cKO)
D
Small intestine
Colon
ECSIT
β-ACTIN
-55KD
-52KD
Ecsit fl/fl
Ecsit cKO
Ecsit rescue
E
Ecsit fl/fl
Ecsit cKO
Ecsit rescue
Body weight(%)
140
120
100
80
60
0
5
10
15
Day
NS
****
F
G
Colon
Ileum
Jejunum

Figure S5. Rescue of ECSIT in intestine reverses the dysfunctional intestine phenotype in ECSIT-deficient mice

(A) Targeting vector design for generation of a mouse strain with *Ecsit-KI*-3Flag $^{fl/+}$ by loxp sites.

(B) Representative genotyping image for analysis of *Ecsit-KI*-3Flag loxp site.

(C) Schematic of mice hybridization strategy to generate *Ecsit* ^{rescue} mice.

(D) Immunoblotting analysis of ECSIT and β-ACTIN (loading control) in intestinal epithelium from indicated mice on day17 after first tamoxifen injection.

(E) Changes in body weight of tamoxifen-treated *Ecsit* $^{fl/fl}$, *Ecsit* ^{cKO} and *Ecsit* ^{rescue} littermates (n=8).

(F) Macroscopic image of intestine on day 17 after first tamoxifen injection.

(G) H&E staining of indicated intestine in (F). Scale bar, 100 μm.

Data are pooled from three independent experiments (E). Data are representative of three independent experiments (F and G). Error bars show mean ± SEM. ****$P \leq 0.0001$, NS, not significant. Two-way ANOVA test for E.

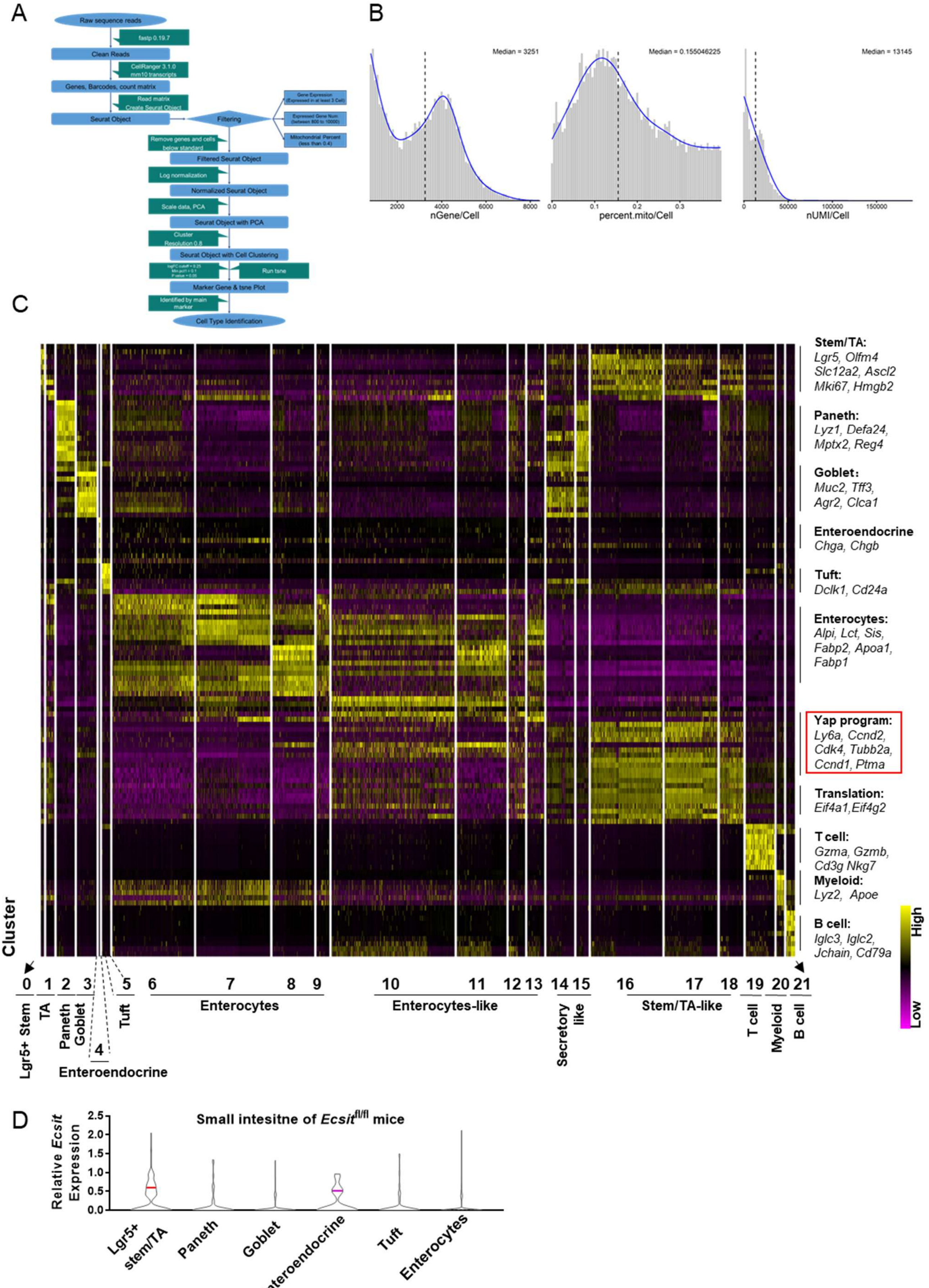

A
Raw sequence reads
fastp 0.19.7
Clean Reads
CellRanger 3.1.0 mm10 transcripts
Genes, Barcodes, count matrix
Read matrix Create Seurat Object
Seurat Object
Filtering
Remove genes and cells below standard
Filtered Seurat Object
Log normalization
Normalized Seurat Object
Scale data, PCA
Seurat Object with PCA
Cluster Resolution 0.8
Seurat Object with Cell Clustering
Run tsne
Marker Gene & tsne Plot
Identified by main marker
Cell Type Identification
B
Median = 3251
nGene/Cell
Median = 0.155046225
percent.mito/Cell
Median = 13145
nUMI/Cell
C
Stem/TA:
Lgr5, Olfm4
Slc12a2, Ascl2
Mki67, Hmgb2
Paneth:
Lyz1, Defa24,
Mptx2, Reg4
Goblet:
Muc2, Tff3,
Agr2, Clca1
Enteroendocrine
Chga, Chgb
Tuft:
Dclk1, Cd24a
Enterocytes:
Alpi, Lct, Sis,
Fabp2, Apoa1,
Fabp1
Yap program:
Ly6a, Ccnd2,
Cdk4, Tubb2a,
Ccnd1, Ptma
Translation:
Eif4a1,Eif4g2
T cell:
Gzma, Gzmb,
Cd3g Nkg7
Myeloid:
Lyz2, Apoe
B cell:
Iglc3, Iglc2,
Jchain, Cd79a
High
Low
Cluster
0 1 2 3 5 6 7 8 9 10 11 12 13 14 15 16 17 18 19 20 21
4
Lgr5+ Stem
TA
Paneth
Goblet
Tuft
Enteroendocrine
Enterocytes
Enterocytes-like
Secretory like
Stem/TA-like
T cell
Myeloid
B cell
D
Small intesitne of Ecsit fl/fl mice
Relative Ecsit Expression
2.5
2.0
1.5
1.0
0.5
0.0
Lgr5+ stem/TA
Paneth
Goblet
Enteroendocrine
Tuft
Enterocytes

Figure S6. Single cell RNA-seq analysis of small intestine

(A) Summary of bioinformatics workflow.

(B) Quality metrics for scRNA-seq data of small intestine. Shown are distributions of the number of genes per cell (up), the percent of mitochondria per cell (middle) and the number of UMI per cell (bottom).

(C) Heatmap showing expression of indicated marker genes that identify distinct cell-types in small intestine.

(D) Violin plot showing the expression of *Ecsit* in the indicated cell type in small intestine from *Ecsit* $^{fl/fl}$ mice.

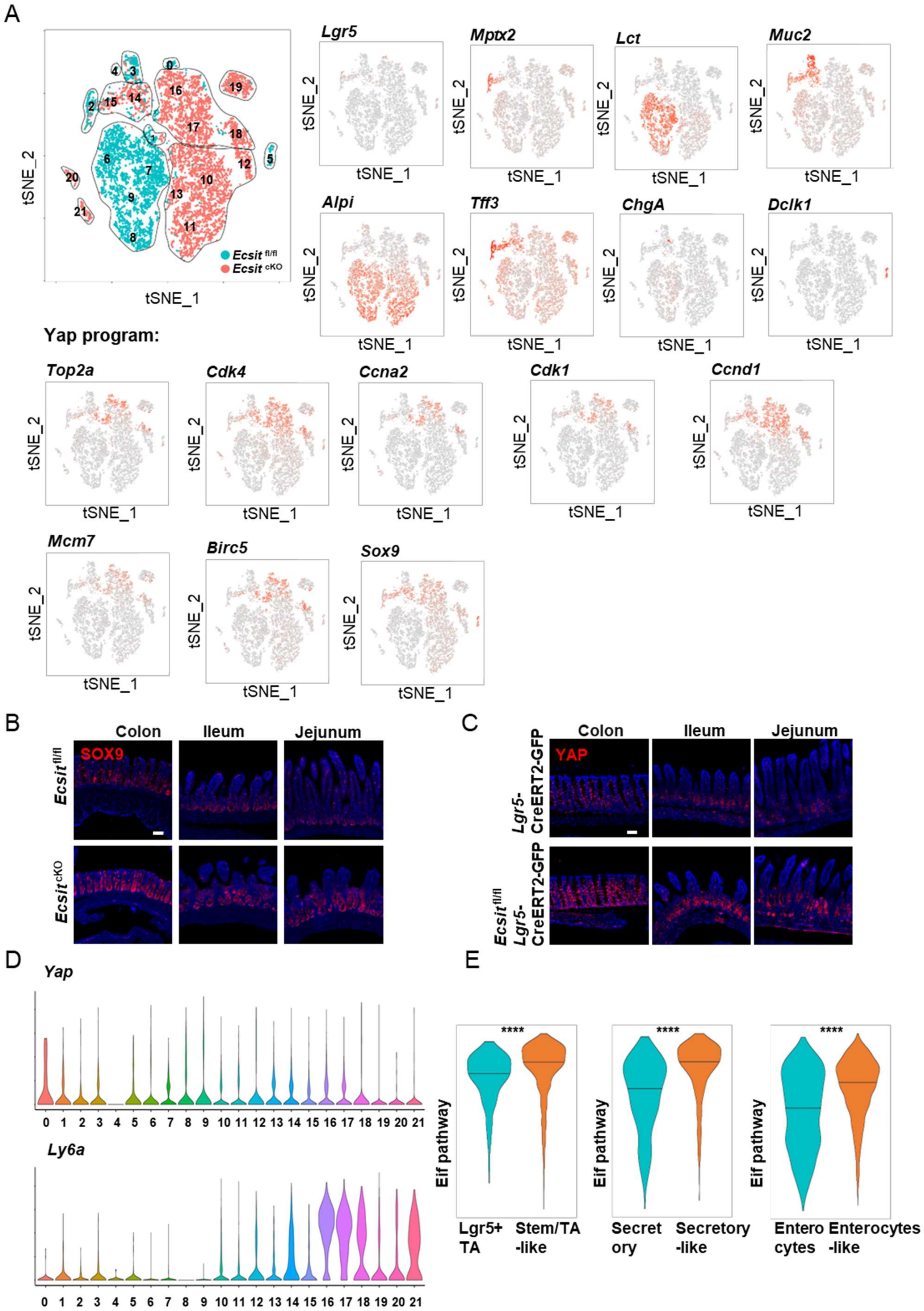
A
Ecsit fl/fl
Ecsit cKO
tSNE_1
tSNE_2
Lgr5
Mptx2
Lct
Muc2
Alpi
Tff3
ChgA
Dclk1
Yap program:
Top2a
Cdk4
Ccna2
Cdk1
Ccnd1
Mcm7
Birc5
Sox9
B
Colon
Ileum
Jejunum
SOX9
Ecsit fl/fl
Ecsit cKO
C
YAP
Lgr5-CreERT2-GFP
Ecsit fl/fl Lgr5-CreERT2-GFP
D
Yap
Ly6a
E
Eif pathway
Lgr5+ TA
Stem/TA -like
Secretory
Secretory -like
Enterocytes
Enterocytes -like
****

Figure S7. ECSIT deficiency enhances YAP translation and signature in small intestine epithelium

(A) Expression of indicated genes is plotted on a t-SNE graph of small intestinal epithelium.

(B-C) Immunofluorescence staining for SOX9 (B) and YAP (C) of colon, ileum and jejunum respectively from indicated mice. Nuclei were labeled with DAPI (blue). Scale bars, 100 μm.

(D) Violin plot showing expression of indicated genes in each cluster of small intestinal epithelium.

(E) ssGSEA analysis of Eif pathway signature of indicated cell type in small intestinal epithelium. ****$P \leq 0.0001$. Two-tailed unpaired student's *t*-test.

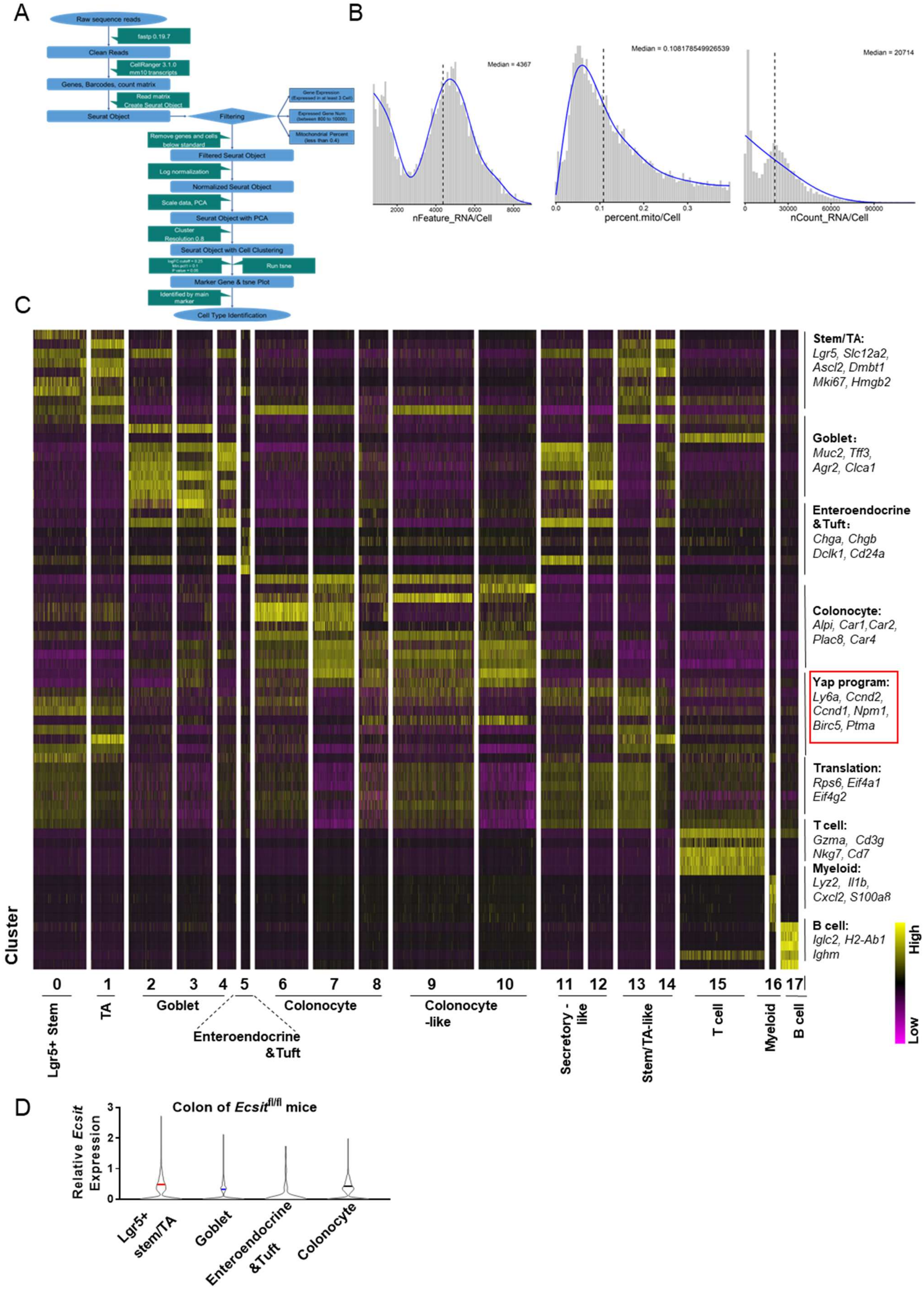
A
Raw sequence reads
fastp 0.19.7
Clean Reads
CellRanger 3.1.0
mm10 transcripts
Genes, Barcodes, count matrix
Read matrix
Create Seurat Object
Seurat Object
Filtering
Gene Expression (Expressed in at least 3 Cell)
Expressed Gene Num (between 800 to 10000)
Mitochondrial Percent (less than 0.4)
Remove genes and cells below standard
Filtered Seurat Object
Log normalization
Normalized Seurat Object
Scale data, PCA
Seurat Object with PCA
Cluster Resolution 0.8
Seurat Object with Cell Clustering
Run tsne
Marker Gene & tsne Plot
Identified by main marker
Cell Type Identification
B
Median = 4367
nFeature_RNA/Cell
Median = 0.108178549926539
percent.mito/Cell
Median = 20714
nCount_RNA/Cell
C
Stem/TA:
Lgr5, Slc12a2, Ascl2, Dmbt1 Mki67, Hmgb2
Goblet:
Muc2, Tff3, Agr2, Clca1
Enteroendocrine &Tuft:
Chga, Chgb Dclk1, Cd24a
Colonocyte:
Alpi, Car1,Car2, Plac8, Car4
Yap program:
Ly6a, Ccnd2, Ccnd1, Npm1, Birc5, Ptma
Translation:
Rps6, Eif4a1 Eif4g2
T cell:
Gzma, Cd3g Nkg7, Cd7
Myeloid:
Lyz2, Il1b, Cxcl2, S100a8
B cell:
Iglc2, H2-Ab1 Ighm
High
Low
Cluster
0 1 2 3 4 5 6 7 8 9 10 11 12 13 14 15 16 17
Lgr5+ Stem
TA
Goblet
Enteroendocrine &Tuft
Colonocyte
Colonocyte -like
Secretory - like
Stem/TA-like
T cell
Myeloid
B cell
D
Colon of Ecsit fl/fl mice
Relative Ecsit Expression
Lgr5+ stem/TA
Goblet
Enteroendocrine &Tuft
Colonocyte

Figure S8. Single cell RNA-seq analysis of colon

(A) Summary of bioinformatics workflow.

(B) Quality metrics for scRNA-seq data of colon. Shown are distributions of the number of genes per cell (left), the percent of mitochondria per cell (middle) and the number of UMI per cell (right).

(C) Heatmap showing expression of indicated marker genes that identify distinct cell-types in colon.

(D) Violin plot showing the expression of *Ecsit* in the indicated cell type in colon from *Ecsit* $^{fl/fl}$ mice.

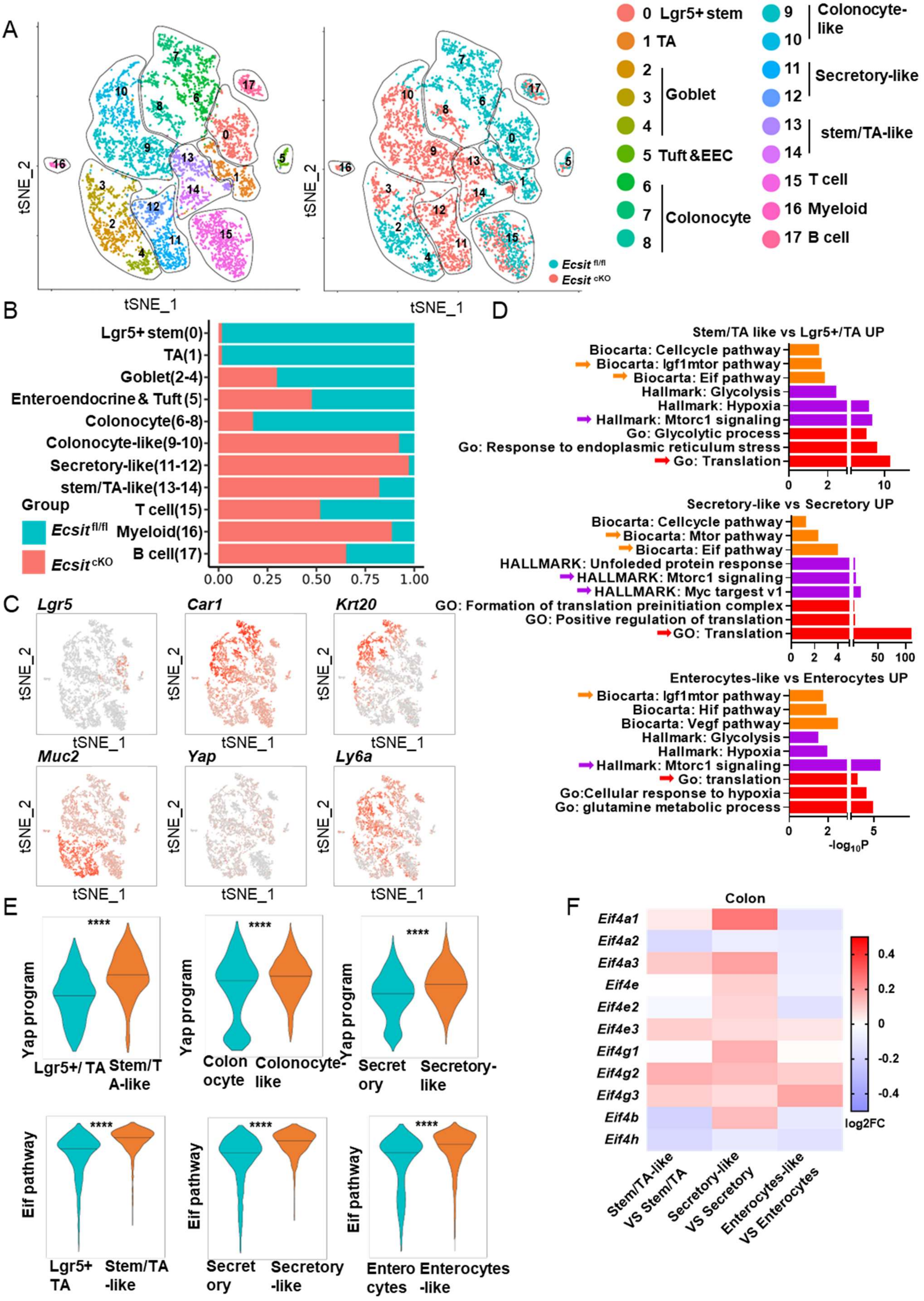
A
B
C
D
E
F
0 Lgr5+ stem
1 TA
2 3 4 Goblet
5 Tuft &EEC
6 7 8 Colonocyte
9 10 Colonocyte-like
11 12 Secretory-like
13 14 stem/TA-like
15 T cell
16 Myeloid
17 B cell
Ecsit fl/fl
Ecsit cKO
tSNE_1
tSNE_2
Lgr5+ stem(0)
TA(1)
Goblet(2-4)
Enteroendocrine & Tuft (5)
Colonocyte(6-8)
Colonocyte-like(9-10)
Secretory-like(11-12)
stem/TA-like(13-14)
T cell(15)
Myeloid(16)
B cell(17)
Group
Lgr5
Car1
Krt20
Muc2
Yap
Ly6a
Stem/TA like vs Lgr5+/TA UP
Biocarta: Cellcycle pathway
Biocarta: Igf1mtor pathway
Biocarta: Eif pathway
Hallmark: Glycolysis
Hallmark: Hypoxia
Hallmark: Mtorc1 signaling
Go: Glycolytic process
Go: Response to endoplasmic reticulum stress
Go: Translation
Secretory-like vs Secretory UP
Biocarta: Cellcycle pathway
Biocarta: Mtor pathway
Biocarta: Eif pathway
HALLMARK: Unfoleded protein response
HALLMARK: Mtorc1 signaling
HALLMARK: Myc targest v1
GO: Formation of translation preinitiation complex
GO: Positive regulation of translation
GO: Translation
Enterocytes-like vs Enterocytes UP
Biocarta: Igf1mtor pathway
Biocarta: Hif pathway
Biocarta: Vegf pathway
Hallmark: Glycolysis
Hallmark: Hypoxia
Hallmark: Mtorc1 signaling
Go: translation
Go:Cellular response to hypoxia
Go: glutamine metabolic process
-log10P
Yap program
Lgr5+/ TA
Stem/TA-like
Colonocyte
Colonocyte-like
Secretory
Secretory-like
Eif pathway
Lgr5+ TA
Stem/TA -like
Secretory
Secretory -like
Enterocytes
Enterocytes -like
****
Colon
Eif4a1
Eif4a2
Eif4a3
Eif4e
Eif4e2
Eif4e3
Eif4g1
Eif4g2
Eif4g3
Eif4b
Eif4h
log2FC
Stem/TA-like VS Stem/TA
Secretory-like VS Secretory
Enterocytes-like VS Enterocytes

Figure S9. ECSIT deficiency facilitates Translation and Eif pathway and high YAP program in colonic epithelium

(A) Unsupervised clustering of scRNA-seq data from whole epithelia of colon on day 17 after first tamoxifen injection. (n=7,845 single-cell transcriptomes, cells were mixed from 3 mice). Unsupervised clusters are overlaid on the t-SNE map and are indicated by different colors and labeled according to their identity based on the expression of cell-type-specific marker-gene expression (left). Cells from *Ecsit* $^{fl/fl}$ (blue dots) or *Ecsit* ^{cKO} intestinal epithelium (red dots) are also plotted as indicated (right).

(B) Bar graph showing frequency of *Ecsit* $^{fl/fl}$ (blue) or *Ecsit* ^{cKO} (red) cells of each cell type in colonic epithelium.

(C) Expression of indicated genes is plotted on a t-SNE graph of colonic epithelium.

(D) Pathway enrichment analysis of indicated cell types based on scRNA-seq data of colonic epithelium.

(E) ssGSEA analysis of YAP program (up) and Eif pathway (bottom) signature of indicated cell type in colonic epithelium.

(F) Heatmap showing the indicated gene expression between cell types based on scRNA-seq data of colonic epithelium.

****$P \leq 0.0001$. Two-tailed unpaired student's *t*-test.

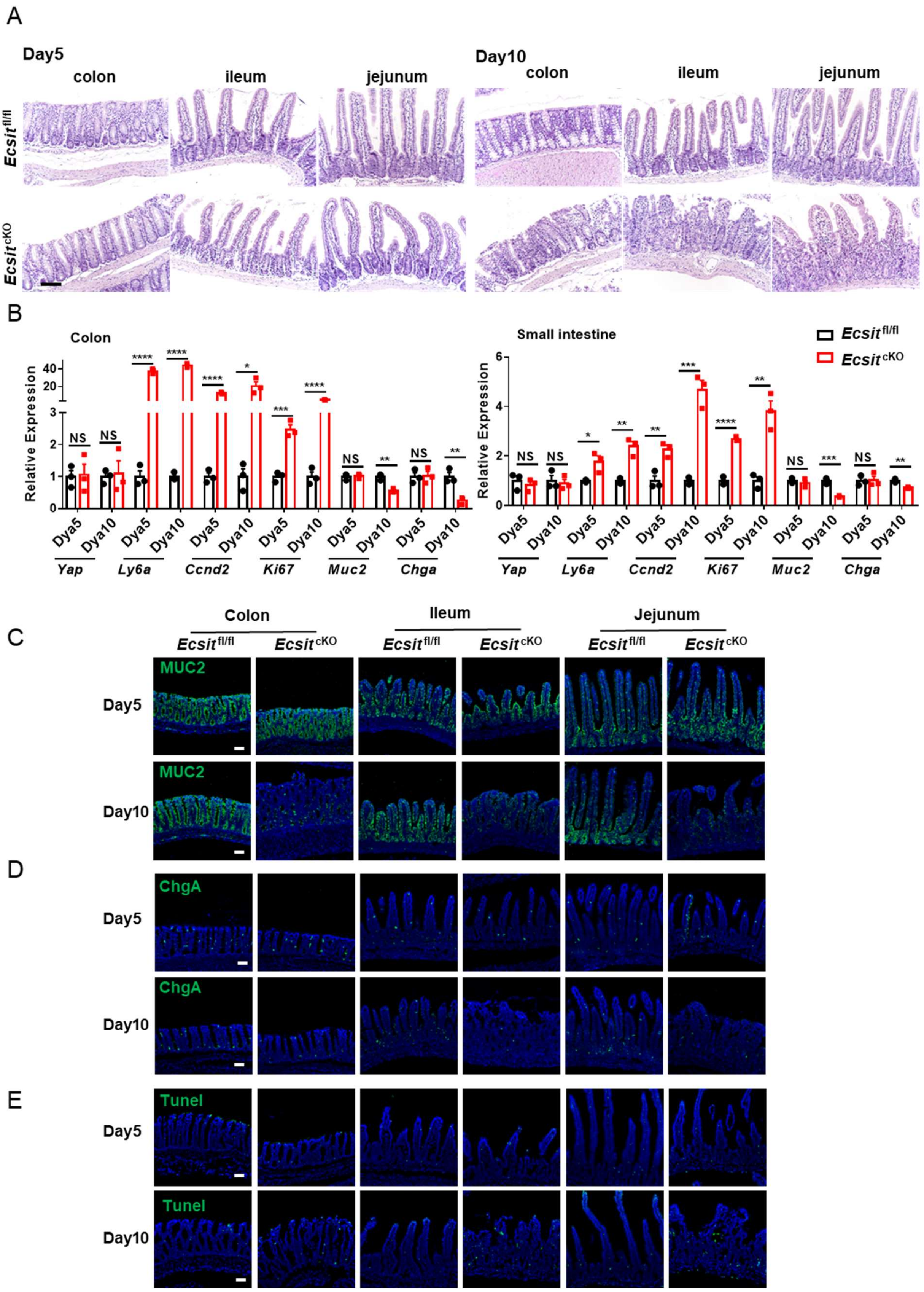
A
Day5
colon
ileum
jejunum
Day10
colon
ileum
jejunum
Ecsit fl/fl
Ecsit cKO
B
Colon
Small intestine
Ecsit fl/fl
Ecsit cKO
Relative Expression
NS
*
**
***
****
Dya5
Dya10
Yap
Ly6a
Ccnd2
Ki67
Muc2
Chga
C
Colon
Ileum
Jejunum
Ecsit fl/fl
Ecsit cKO
MUC2
Day5
Day10
D
ChgA
Day5
Day10
E
Tunel
Day5
Day10

Figure S10. Intestinal pathology, intestinal epithelium differentiation and death on day 5 and 10 after the first TAM injection

(A) H&E staining of indicated intestine of 8-week-old mice on day5 and day10 after first tamoxifen injection. Scale bars, 100 μm.

(B) qPCR analysis of indicated gene expression in small intestinal and colonic epithelium (n=3).

(C-D) Representative images of colon, ileum and jejunum from the indicated mice stained with the indicated antibodies. Scale bars, 100 μm.

(E) Representative images of colon, ileum and jejunum from the indicated mice stained by Terminal deoxynucleotidyl transferase dUTP nick end labeling (Tunel). Scale bars, 100 μm.

Data are representative of three independent experiments (A, C-E). Error bars show mean ± SEM. *$P \leq 0.05$, **$P \leq 0.01$, ***$P \leq 0.001$, ****$P \leq 0.0001$, NS, not significant. Two-tailed unpaired student's *t*-test.

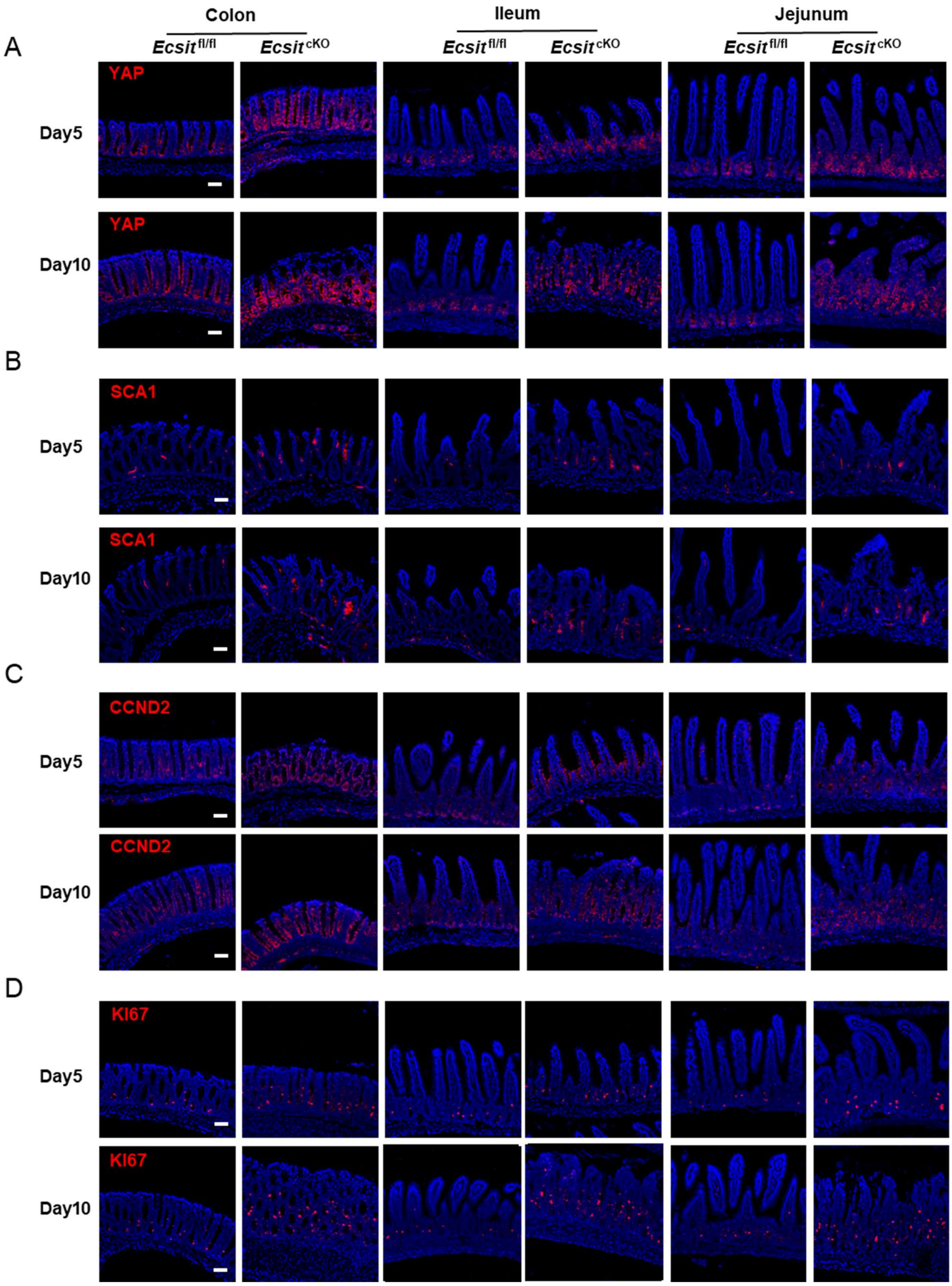
Colon
Ileum
Jejunum
A
Ecsit fl/fl
Ecsit cKO
YAP
Day5
Day10
B
SCA1
C
CCND2
D
KI67

Figure S11. Expression of YAP and its targets on Day 5 and 10 after the first TAM injection

(A-D) Representative images of colon, ileum and jejunum from the indicated mice stained with the indicated antibodies. Scale bars, 100 μm.

Data are representative of three independent experiments.

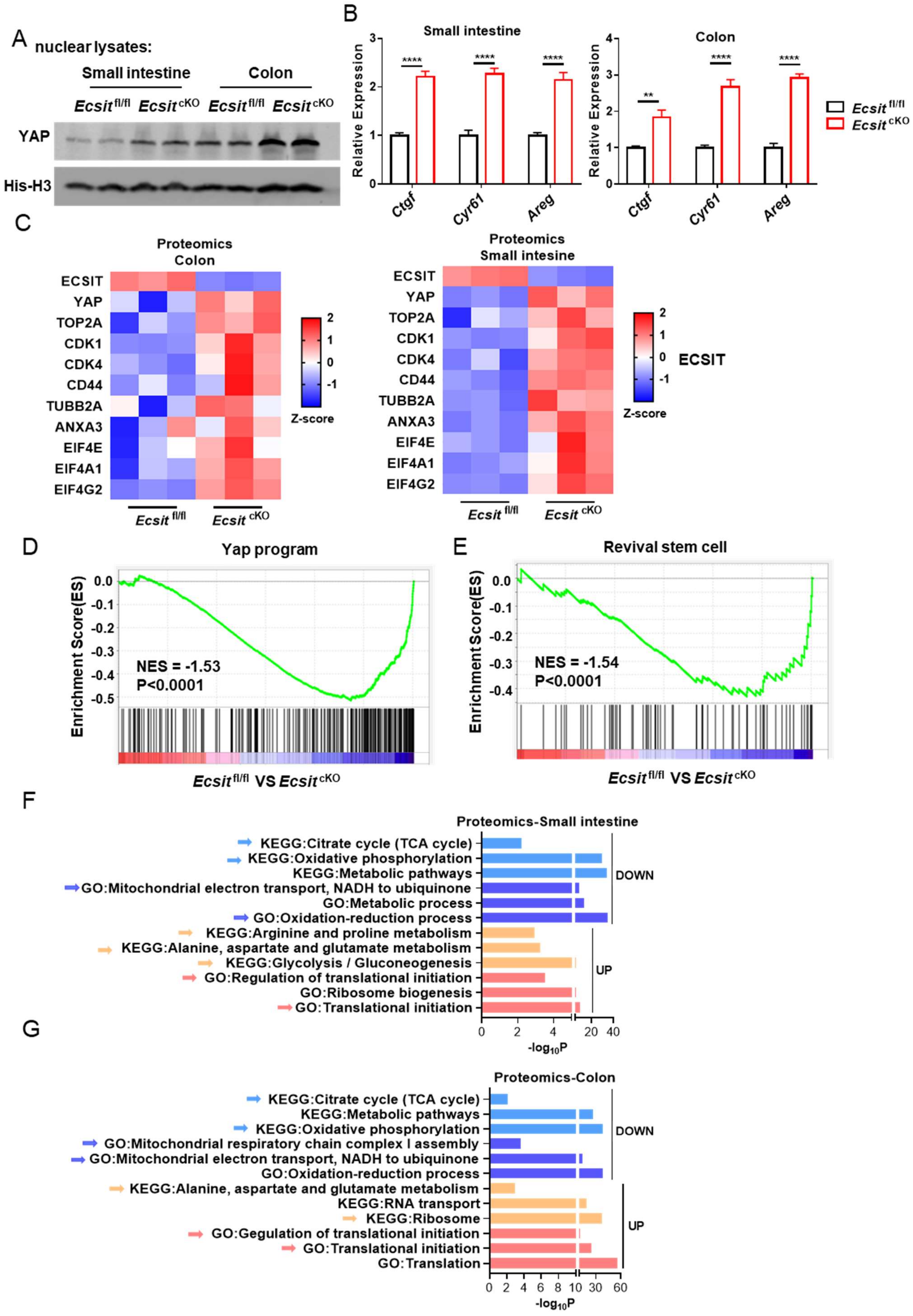
A nuclear lysates:
Small intestine
Colon
Ecsit fl/fl Ecsit cKO Ecsit fl/fl Ecsit cKO
YAP
His-H3
B
Small intestine
Colon
Relative Expression
Ctgf
Cyr61
Areg
Ecsit fl/fl
Ecsit cKO
C
Proteomics Colon
Proteomics Small intesine
ECSIT
YAP
TOP2A
CDK1
CDK4
CD44
TUBB2A
ANXA3
EIF4E
EIF4A1
EIF4G2
Z-score
ECSIT
Ecsit fl/fl
Ecsit cKO
D
Yap program
Enrichment Score(ES)
NES = -1.53
P<0.0001
Ecsit fl/fl VS Ecsit cKO
E
Revival stem cell
NES = -1.54
P<0.0001
Ecsit fl/fl VS Ecsit cKO
F
Proteomics-Small intestine
KEGG:Citrate cycle (TCA cycle)
KEGG:Oxidative phosphorylation
KEGG:Metabolic pathways
GO:Mitochondrial electron transport, NADH to ubiquinone
GO:Metabolic process
GO:Oxidation-reduction process
KEGG:Arginine and proline metabolism
KEGG:Alanine, aspartate and glutamate metabolism
KEGG:Glycolysis / Gluconeogenesis
GO:Regulation of translational initiation
GO:Ribosome biogenesis
GO:Translational initiation
DOWN
UP
-log10P
G
Proteomics-Colon
KEGG:Citrate cycle (TCA cycle)
KEGG:Metabolic pathways
KEGG:Oxidative phosphorylation
GO:Mitochondrial respiratory chain complex I assembly
GO:Mitochondrial electron transport, NADH to ubiquinone
GO:Oxidation-reduction process
KEGG:Alanine, aspartate and glutamate metabolism
KEGG:RNA transport
KEGG:Ribosome
GO:Gegulation of translational initiation
GO:Translational initiation
GO:Translation
DOWN
UP
-log10P

Figure S12. Proteomics analysis of ECSIT-deficient intestine

(A) Immunoblotting analysis of the level of YAP in nuclear lysates of intestinal epithelium from indicated mice on day17 after first tamoxifen injection. Histone H3 (His-H3) was used as a loading control.

(B) qPCR analysis of indicated gene expression in small intestinal and colonic epithelium from indicated mice on day17 after first tamoxifen injection (n=6).

(C) Heatmap showing the indicated protein expression of indicated based on proteomics data of small intestinal (left) and colonic (right) epithelium (n=3).

(D) GSEA analysis for the proteins associated with YAP program based on proteomics data.

(E) GSEA analysis for the proteins associated with revival stem cell based on proteomics data.

(F) Pathway enrichment analysis based on proteomics data of small intestinal epithelium.

(G) Pathway enrichment analysis based on proteomics data of colonic epithelium.

Data are representative of three independent experiments for (A). Error bars show mean ± SEM. $^{**}P \leq 0.01$, $^{****}P \leq 0.0001$. Two-tailed unpaired student's *t*-test.

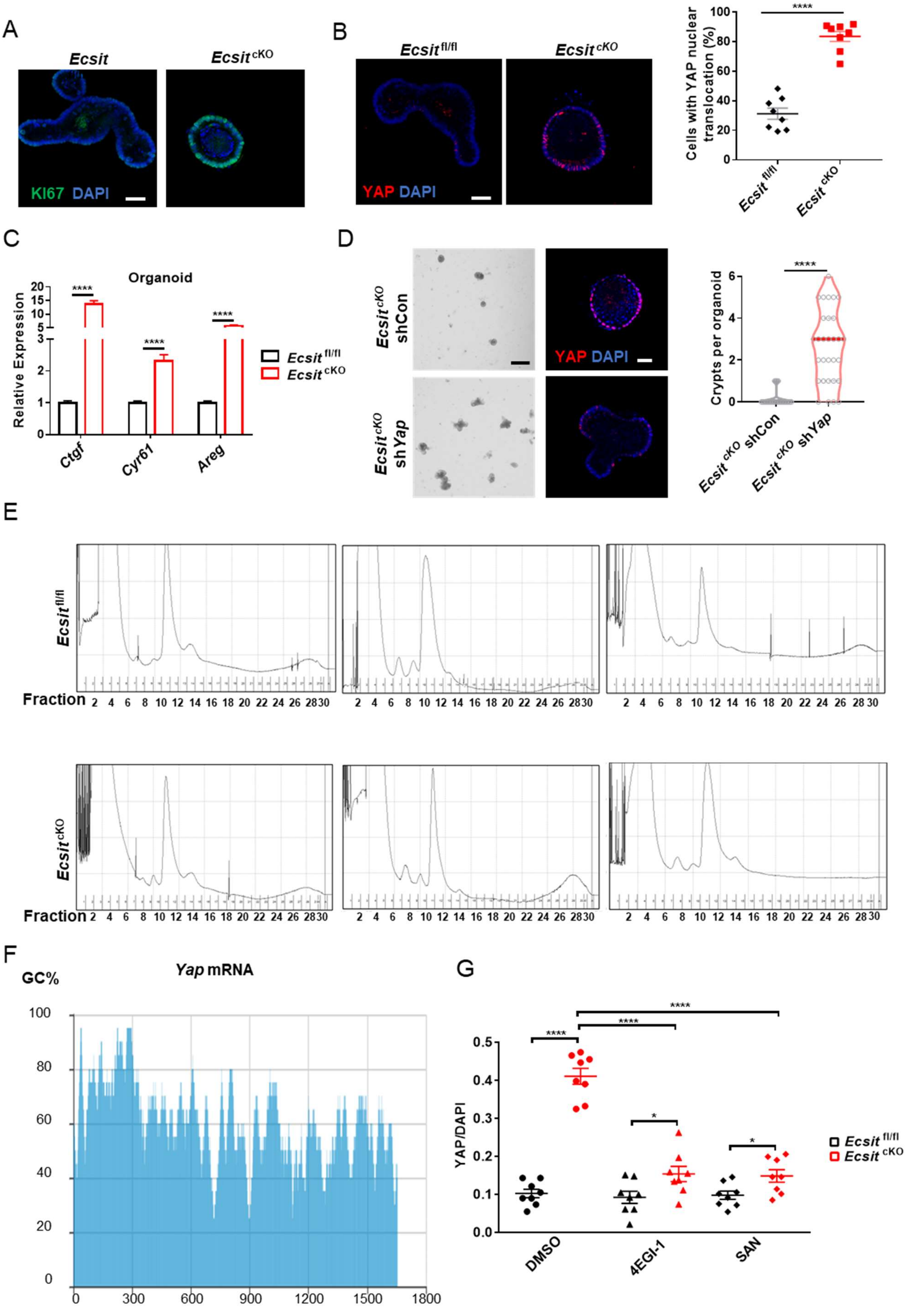

A
Ecsit
Ecsit cKO
KI67 DAPI
B
Ecsit fl/fl
Ecsit cKO
YAP DAPI
Cells with YAP nuclear translocation (%)
****
C
Organoid
Relative Expression
****
Ctgf
Cyr61
Areg
Ecsit fl/fl
Ecsit cKO
D
Ecsit cKO shCon
Ecsit cKO shYap
YAP DAPI
Crypts per organoid
****
E
Ecsit fl/fl
Ecsit cKO
Fraction
F
GC%
Yap mRNA
G
YAP/DAPI
****
*
DMSO
4EGI-1
SAN
Ecsit fl/fl
Ecsit cKO

Figure S13. ECSIT regulates YAP protein translation and expression through eIF4F complex

(A) Immunofluorescent staining of KI67 in organoids. Nuclei were labeled with DAPI (blue). Scale bar, 20 μm.

(B) Left: Immunofluorescent staining of YAP in organoids. Nuclei were labeled with DAPI (blue). Scale bar, 20 μm. Right: quantification of cells with YAP nuclear translocation (n=8).

(C) qPCR analysis of indicated gene expression in organoids from indicated mice (n=6).

(D) *Ecsit* ^{cKO} organoids were infected with lentiviral of sh*Yap* expressing pLKO.1 vector or control vector. Left: Organoids were photographed in bright-field and Immunofluorescent staining of YAP (red). Nuclei were labeled with DAPI (blue). Scale bars, 100 μm (BF); 20 μm (IF). Right: Quantification of crypts per organoid (n=30).

(E) Polysome profiling of global translation efficiency in the indicated genotypes. Sucrose gradient absorbance (A260) was monitored in each fraction.

(F) The content of GC at the 5' end of *Yap* mRNA.

(G) Quantification of immunofluorescent staining for YAP in organoids after treatment with 4EGI-1 and SAN (n=8).

Data are representative of three independent experiments for (A, B and D). Error bars show mean ± SEM. *$P \leq 0.05$, ****$P \leq 0.0001$. Two-tailed unpaired student's *t*-test.

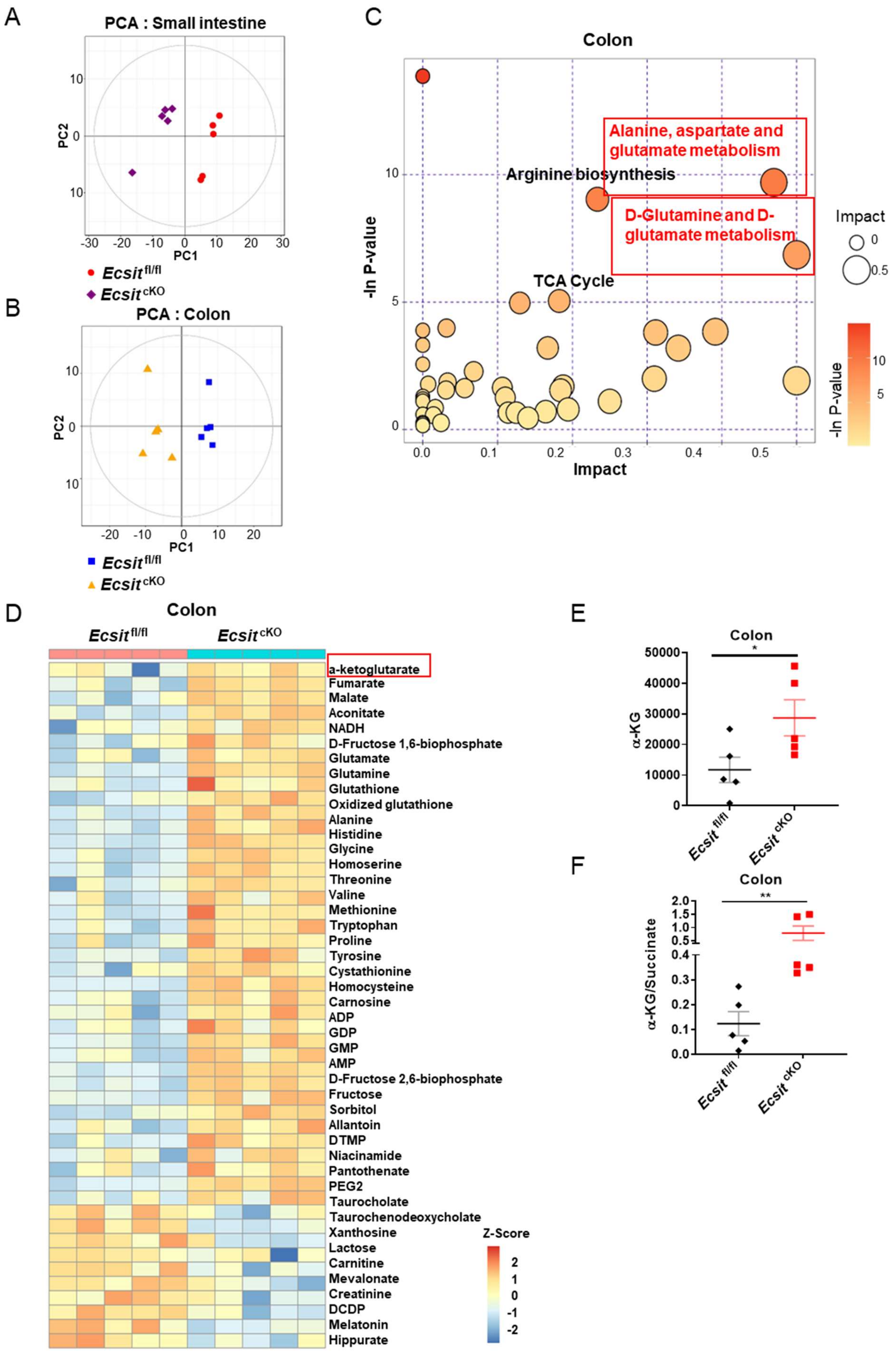

A
PCA : Small intestine
PC2
PC1
Ecsit fl/fl
Ecsit cKO
B
PCA : Colon
C
Colon
Alanine, aspartate and glutamate metabolism
Arginine biosynthesis
D-Glutamine and D-glutamate metabolism
TCA Cycle
-ln P-value
Impact
D
Colon
a-ketoglutarate
Fumarate
Malate
Aconitate
NADH
D-Fructose 1,6-biophosphate
Glutamate
Glutamine
Glutathione
Oxidized glutathione
Alanine
Histidine
Glycine
Homoserine
Threonine
Valine
Methionine
Tryptophan
Proline
Tyrosine
Cystathionine
Homocysteine
Carnosine
ADP
GDP
GMP
AMP
D-Fructose 2,6-biophosphate
Fructose
Sorbitol
Allantoin
DTMP
Niacinamide
Pantothenate
PEG2
Taurocholate
Taurochenodeoxycholate
Xanthosine
Lactose
Carnitine
Mevalonate
Creatinine
DCDP
Melatonin
Hippurate
Z-Score
E
Colon
α-KG
F
Colon
α-KG/Succinate

Figure S14. Metabolomic analysis of ECSIT-deficient intestine

(A-B) PCA analysis of metabolomics data of small intestinal (A) and colonic (B) epithelium.

(C) Pathway enrichment analysis of changed metabolites between colonic epithelium of *Ecsit* $^{fl/fl}$ and *Ecsit* ^{cKO} mice (n=5).

(D) The heatmap displays the relative abundance of significantly changed metabolites in colonic epithelium of *Ecsit* $^{fl/fl}$ and *Ecsit* ^{cKO} mice (n=5 biologically independent experiments).

(E-F) Quantification of α-KG (E, n=5) and the ratio of α-KG/succinate ratio (F, n=5).
Error bars show mean ± SEM. $^*P \leq 0.05$, $^{**}P \leq 0.01$. Two-tailed unpaired student's *t*-test.

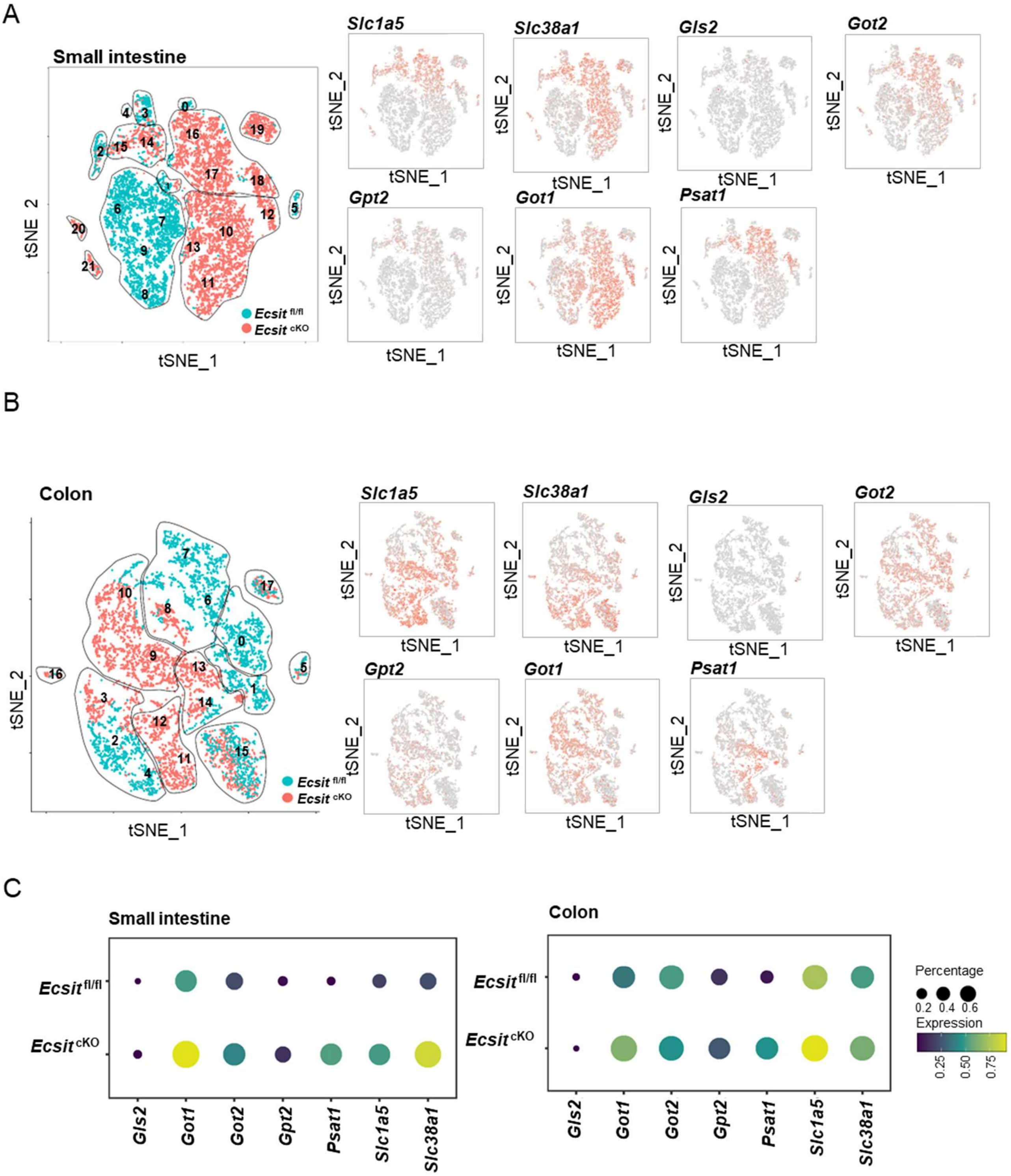
A
Small intestine
Slc1a5
Slc38a1
Gls2
Got2
Gpt2
Got1
Psat1
tSNE_1
tSNE_2
Ecsit fl/fl
Ecsit cKO
B
Colon
C
Percentage
0.2 0.4 0.6
Expression
0.25 0.50 0.75
Gls2 Got1 Got2 Gpt2 Psat1 Slc1a5 Slc38a1

Figure S15. Glutamate metabolism related genes are enriched in ECSIT-deficient intestine

(A) Expression of indicated genes related to glutamate metabolism is plotted on a t-SNE graph of small intestinal epithelium.

(B) Expression of indicated genes related to glutamate metabolism is plotted on a t-SNE graph of colonic epithelium.

(C) Bubble plot showing expression of non-immune cells of indicated genes related to glutamate metabolism between *Ecsit* $^{fl/fl}$ and *Ecsit* ^{cKO} small intestinal (left) and colonic (right) epithelium.

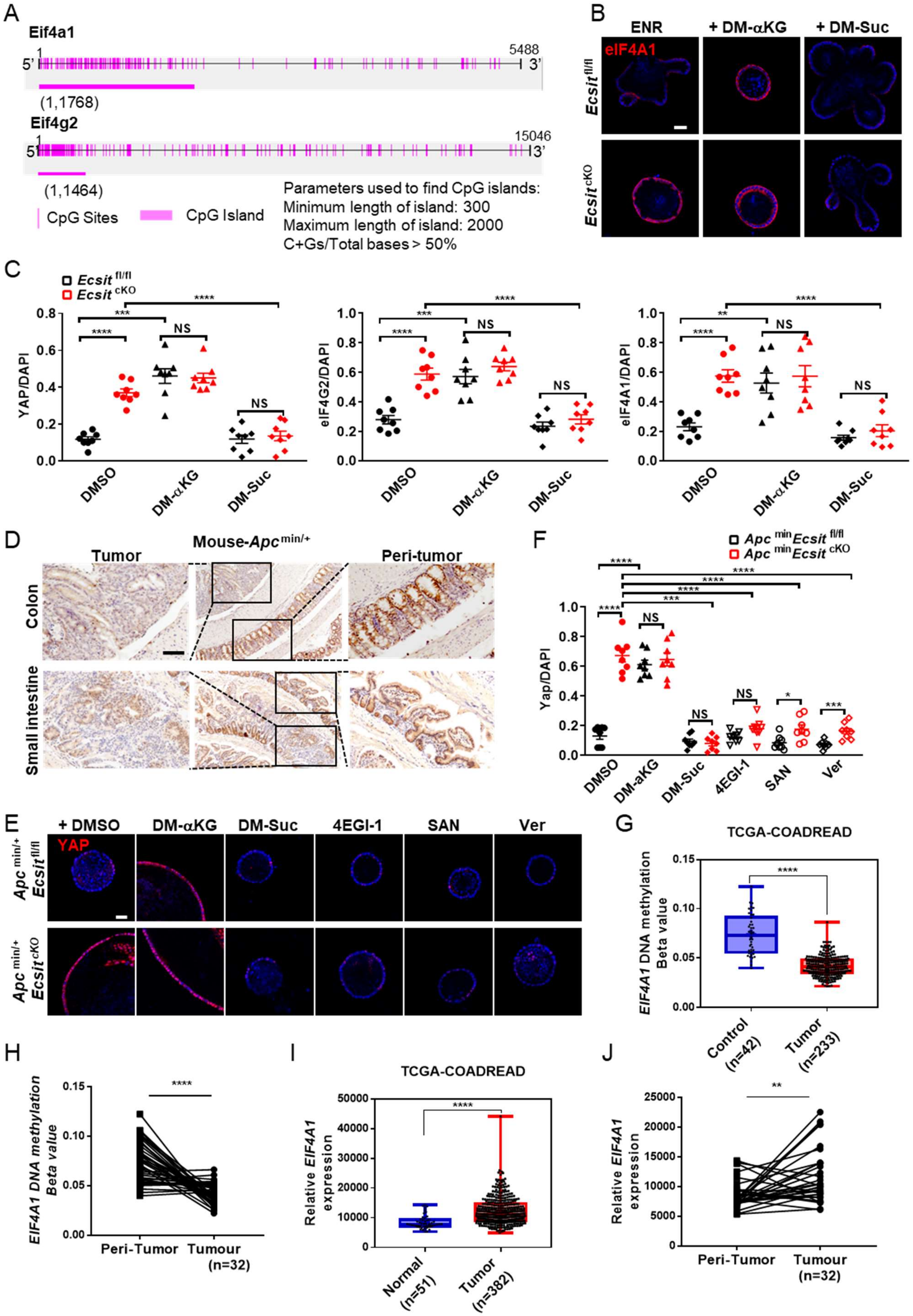

A
Eif4a1
5488
5'
3'
(1,1768)
Eif4g2
15046
(1,1464)
CpG Sites
CpG Island
Parameters used to find CpG islands:
Minimum length of island: 300
Maximum length of island: 2000
C+Gs/Total bases > 50%
B
ENR
+ DM-αKG
+ DM-Suc
eIF4A1
Ecsit fl/fl
Ecsit cKO
C
Ecsit fl/fl
Ecsit cKO
YAP/DAPI
eIF4G2/DAPI
eIF4A1/DAPI
NS
DMSO
DM-αKG
DM-Suc
D
Mouse-Apc min/+
Tumor
Peri-tumor
Colon
Small intestine
F
Apc min Ecsit fl/fl
Apc min Ecsit cKO
Yap/DAPI
DMSO
DM-aKG
DM-Suc
4EGI-1
SAN
Ver
E
+ DMSO
DM-αKG
DM-Suc
4EGI-1
SAN
Ver
YAP
Apc min/+ Ecsit fl/fl
Apc min/+ Ecsit cKO
G
TCGA-COADREAD
EIF4A1 DNA methylation Beta value
Control (n=42)
Tumor (n=233)
H
EIF4A1 DNA methylation Beta value
Peri-Tumor
Tumour (n=32)
I
TCGA-COADREAD
Relative EIF4A1 expression
Normal (n=51)
Tumor (n=382)
J
Relative EIF4A1 expression
Peri-Tumor
Tumour (n=32)

Figure S16. High ratio of α-KG:succinate is required for the phenotype in ECSIT-deficient intestine, and eIF4A1 is highly expressed in Colorectal adenocarcinoma (COADREAD)

(A) CpG islands of eIF4A1 (up) and eIF4G2 (bottom) found by Methyl Primer Express v1.0 software.

(B) Immunofluorescent staining of eIF4A1 for indicated organoids. Nuclei were labeled with DAPI (blue). Scale bar, 20 μm.

(C) Immunofluorescent staining quantification of YAP, eIF4G2 and eIF4A1 in organoids after treatment with DM-αKG or DM-Suc (n=8).

(D) IHC staining of ECSIT in *Apc* ^{min} intestine. Scale bar, 100 μm.

(E-F) Immunofluorescent staining (E) and quantification (F, n=8) of YAP in tumor organoids after treatment with indicated reagent. Nuclei were labeled with DAPI (blue). Scale bar, 20 μm.

(G) DNA demethylation level of eIF4A1 between tumor tissue and control tissue based on TCGA database.

(H) DNA demethylation level of eIF4A1 between tumor tissue and paired normal tissue based on TCGA database.

(I) Gene expression of eIF4A1 between tumor tissue and control tissue based on TCGA database.

(J) Gene expression of eIF4A1 between tumor tissue and paired normal tissue based on TCGA database.

Data are representative of three independent experiments for (B, D-E). Error bars show mean ± SEM. *$P \leq 0.05$, **$P \leq 0.01$, ***$P \leq 0.001$, ****$P \leq 0.0001$. NS, not significant. Two-tailed student's *t*-test.

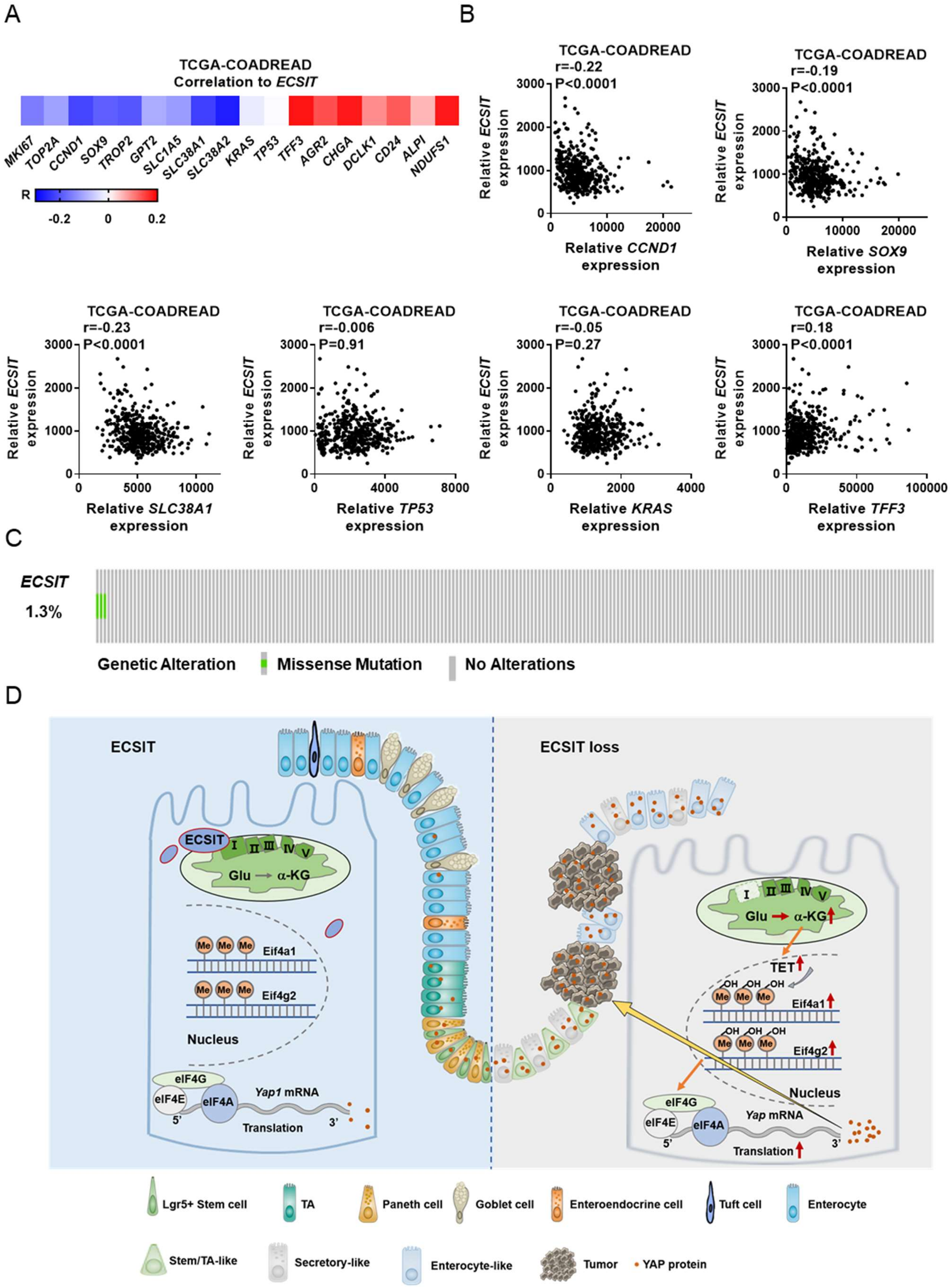
A
TCGA-COADREAD
Correlation to ECSIT
MKI67
TOP2A
CCND1
SOX9
TROP2
GPT2
SLC1A5
SLC38A1
SLC38A2
KRAS
TP53
TFF3
AGR2
CHGA
DCLK1
CD24
ALPI
NDUFS1
R
-0.2
0
0.2
B
TCGA-COADREAD
r=-0.22
P<0.0001
Relative ECSIT expression
Relative CCND1 expression
TCGA-COADREAD
r=-0.19
P<0.0001
Relative SOX9 expression
TCGA-COADREAD
r=-0.23
P<0.0001
Relative SLC38A1 expression
TCGA-COADREAD
r=-0.006
P=0.91
Relative TP53 expression
TCGA-COADREAD
r=-0.05
P=0.27
Relative KRAS expression
TCGA-COADREAD
r=0.18
P<0.0001
Relative TFF3 expression
C
ECSIT
1.3%
Genetic Alteration
Missense Mutation
No Alterations
D
ECSIT
ECSIT loss
Glu → α-KG
Eif4a1
Eif4g2
Nucleus
eIF4G
eIF4E
eIF4A
Yap1 mRNA
Translation
TET
Yap mRNA
Lgr5+ Stem cell
TA
Paneth cell
Goblet cell
Enteroendocrine cell
Tuft cell
Enterocyte
Stem/TA-like
Secretory-like
Enterocyte-like
Tumor
YAP protein

Figure S17. ECSIT as a potential independent prognostic biomarker for CRC, and the model in this study

(A) Heatmap showing the correlation to indicated genes of *ECSIT* based on TCGA data.

(B) Correlation analysis between *ECSIT* and indicated genes based on TCGA data.

(C) Mutation rate of *ECSIT* in clinical CRC sample based on TCGA data.

(D) Schematic representation of the main findings in this work.

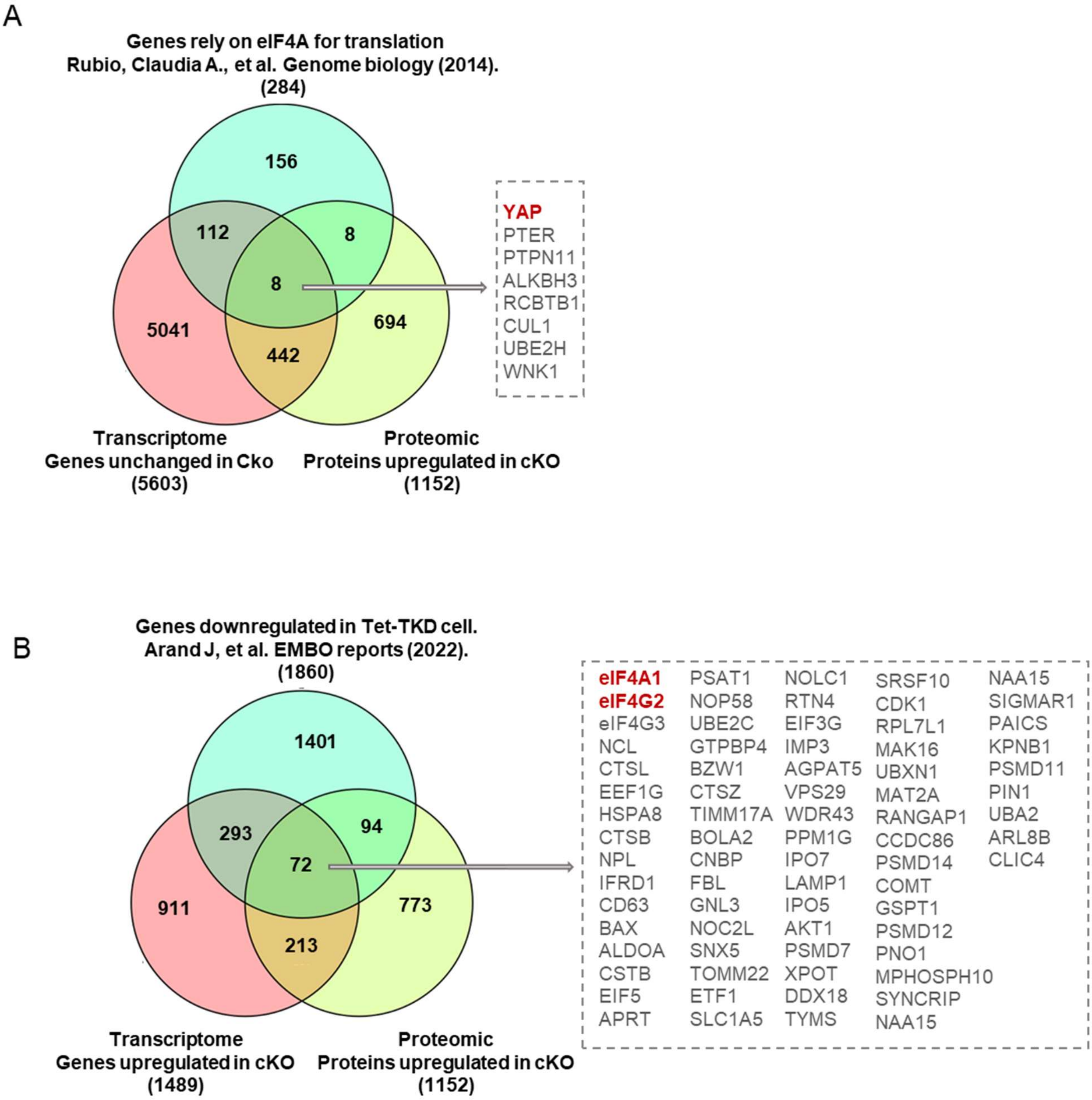

Figure S18. Combined analysis of proteomic and transcriptional data

(A) Venn diagram showing overlapping molecules between the indicated gene sets.

(B) Venn diagram showing overlapping molecules between the indicated gene sets.

Table S1: The gene signature used in ssGSEA analysis

YAP program signature

Gregorieff, et al. Nature 526.7575 (2015): 715-718; Roulis, et al. Nature 580.7804 (2020): 524-529.

Lrp2	Acot10	Fbln5	Stambpl1	Fam149a	1700021K19Rik	Auts2	Sart1	Ppp4c
Flnc	Tbc1d2	Zglp1	B3gnt6	Lmf2	Gm10645	Vps52	Mfhas1	Exoc1
Serpinb9b	Osbpl3	Krt6b	Snrnp35	Dapp1	Btaf1	Brsk1	Copg	Mllt11
Cryab	Gm10393	Arhgap28	Arhgdia	Atn1	Gpsm1	Zfp777	Ppp1ca	Safb
Ggta1	Aph1c	Gm5449	Prkch	Lifr	Tlcd1	Htra2	Dsc2	1110037F02Rik
Serpinb6b	Tgfbr2	Plxna4	Ccdc15	Fam110b	B3galt2	Tab2	Snapc3	Bcas2
Plau	Gpr64	Elf5	4931406P16Rik	Insl3	Efr3a	R3hdm2	Eif1ax	A430107O13Rik
Ly6c1	6030419C18Rik	Chrne	Trim34a	Atf4	Gapvd1	Pygo2	Zfp1	Hus1b
Wnt7a	Oas1b	Dtna	Ywhaz	Gm5263	Bms1	Rnf111	Rab28	Gm10094
Lor	4930572J05Rik	Taar9	Oraov1	Zfp217	Tnpo2	Mars	Ppan	Dpysl3
Btn1a1	Cdh16	Gm10399	Vcl	AI837181	Zdhhc17	Ccdc21	Ints1	Ctdnep1
Akap2	Mettl7b	Ctsw	Antxr2	Ehbp1l1	4732471D19Rik	Ccdc130	Wdr38	39515
AF064781	K230010J24Rik	Scel	Lrsam1	Slc25a30	A230046K03Rik	Eif2s3x	Ccdc12	Ddx42
P2rx2	Gm10691	Cpeb1	Cdh1	2610036A22Rik	Gm16181	Ankfy1	Usp12	Spag7
Loxl4	Gm14446	Thbs4	Spna2	Gfod1	Reep5	Csde1	Pomgnt1	Mtmr2
Ildr2	Arid5a	Epgn	Slc25a22	Mybbp1a	Bptf	Banp	Dctn2	Epb4.1
Ctgf	Murc	Txk	Rps6ka4	Fer	Snx12	Sfswap	Fcho2	Nemf
Ly6g	Bcl2l1	Sgcb	Zfyve16	Lasp1	Aldh4a1	Rplp2	Cbl	4930415O20Rik
Psca	St6galnac4	Gm13697	Purb	Ttc16	Psmd11	Rps17	BC024978	Rrbp1
6430531B16Rik	1700019L03Rik	Gm12689	A130022J15Rik	Arhgef16	Micall1	Tmem189	Cflar	Romo1
Krt80	Ifit2	Il17c	Dcp1a	Leprotl1	Epn2	Gm8894	Gm550	Limd2
Col4a1	Glipr1	Pcdhb18	Arf6	1300018I17Rik	Rexo2	Utp14b	Spin2	Tnip2
BC067074	Jam2	Icam5	39694	Arhgef2	Grip1	Stk4	Hsd17b7	Rbm15
Ly6c2	Il17re	Casr	Col10a1	Pitpnm1	2310037I24Rik	Npm3	Gm10451	Sip1
Col4a2	Mapk11	E130304I02Rik	Wdr93	Emd	Vars	Scaper	2510012J08Rik	BC048403
Dab2	Syne1	8430432A02Rik	Fam164c	Det1	Dhx32	Gtpbp6	Nsmce1	Ptpra
Ifi202b	Anln	Gm336	Crhr2	Cd151	Strn4	Tbcc	Cd99l2	Setd6
Ehd2	Ssh1	4933433C11Rik	Nek11	Znhit6	Kcnk1	Ube2d3	Tcp11l2	Tnpo3
Fam46b	Mycbpap	Cd80	Hps3	Ttc37	Zfp330	Utp20	Eif2s1	Ckap5
Slc25a43	6030445D17Rik	Ehd4	1700016H13Rik	Ttpal	A330049M08Rik	Mrps18b	Vps26a	Trip11
Amotl2	F2r	Lrrc58	Styxl1	Atg9b	Grwd1	Zfp275	Tceal1	3830431G21Rik
Pkhd1l1	Pla2g15	Hic2	Fbxo15	Naif1	Cdk20	Fbxo46	2200002K05Rik	Eftud2
Ankrd35	Parp12	Akap13	Katnal2	Pfdn4	Ccdc97	Armc8	A730015C16Rik	Mtg1
Synpo2l	Angptl3	Baz1a	Fam71d	Tollip	Sdr39u1	Nf1	Igsf5	Dnajc2
Rassf8	Raph1	Gabbr1	L3mbtl1	Ell	Tbcb	Trappc4	4931422A03Rik	Snapc4
Tigit	Cnksr1	Plin2	Ttc34	Phka2	Cables2	Gata4	Casc3	Zfp11
Htr1d	Tgfb1i1	Thnsl1	Pdlim4	Manbal	Elmo3	Cab39	Tstd2	Usp50
Gcnt1	4933427G17Rik	Cdk6	Erc1	Pnpla2	Ikbkb	A730037C10Rik	Btbd6	Ppap2c
Cyr61	Alppl2	Epb4.1l2	Ralgapa2	Timm23	Slc16a11	Gm2004	Lancl3	1700081L11Rik
Clic3	Eps8l1	Arid5b	Cyld	Nes	B3gnt3	Il23a	Samd1	Plagl2
2610019F03Rik	Krtap4-16	Hlcs	Tbc1d7	Mcl1	Heatr6	Cldn19	4930430F08Rik	Pnldc1
Mtap6	Fsip1	Pag1	Herpud2	Med15	Tatdn2	AI504432	Ubqln1	C130040N14Rik
Fgd3	Cd101	Plxna1	Plagl1	Nudt18	Ugcg	Gm3109	Slc35b4	Dnalc4
Akap5	A930002H24Rik	AK129341	Ctu2	Gm10110	Pcgf1	Alpl	Slc16a5	Cd9
Ly6f	Zcchc18	Arhgef5	Pde4d	Zcchc2	Tom1l2	Pcdhb19	Sgms1	Ctsd
Wwc2	Tg	Meis3	Chst15	Zfp750	Slc6a8	Kif26a	Osta	Tex264
Gbp3	Serpinb8	Trp53tg5	Myo18a	Gm9726	Ski	Cyp2t4	Ppig	Taf6l
Rsad2	Nkain3	Rnf13	Gm2a	Rhobtb3	Hoxb4	Ckm	Rpl18	Golga4
Gm10238	Rab33a	Mbp	Sytl5	Mpp5	Dnase2b	Plekha4	Immp2l	Qpctl
Rbpms	Fscn2	Fosl2	Fank1	Mef2a	Nup62	Papolb	Nudcd1	Nol7
Schip1	Armcx4	Cd52	4930570N19Rik	Serpine1	Fam179b	Gm11007	Cwc15	H2-T23
Slc5a5	Klf2	Slc25a33	Rwdd4a	Rbm28	Arid3b	Sp140	Snrpc	Rbm34
Dusp14	Fam65a	Ina	Hgs	1700024N20Rik	Tmem180	Rgs1	Uba1	Eif3k
Zfp185	F3	Ikbip	Ahdc1	Rbm42	Mdm2	Trpv6	Polr2a	Cpsf3l
Magi2	Dst	Fkbp9	Tdrd7	Cacna1c	Irak2	Gm8787	Tnk1	Kdm5c
Trpm8	Oas1g	Nrp2	Tfip11	Pfkfb3	R3hcc1	Tm6sf1	Wdr36	Mrpl17
39509	Pxmp2	Prkcd	Atp2b1	9130404H23Rik	Dis3l2	Dsg1b	Zcrb1	Dctd
Usp18	Tnks1bp1	9130017N09Rik	Zfp941	Odf2l	Stambp	Hmgcll1	Clec16a	Yeats4
Yap1	Myo1h	Traf4	Lypd6b	Myo5b	Ngdn	Lat2	Mlph	Map3k10
Il33	Endod1	Fstl3	Mnat1	4933403G14Rik	Cdk2ap2	Hist1h4c	Ccdc154	Lmbrd1
Tspan2	1700016K19Rik	Tnfaip3	Mob3a	Riok1	Trim56	Ankmy1	Plekhg4	Zfp219
Apol9a	Rasl11a	Pstpip1	Adam9	Pip4k2b	Stat5b	Zfp286	Gm10116	Qtrtd1
Adam8	Kctd10	Cpne3	Ocrl	Fam84b	Xpo6	Klf17	Crybb3	Gga3
Apol9b	Gpc1	Sh3bgr	Dnajb6	Dapk1	Stau2	Zfp580	Hist1h4i	Hnrnpab
Rassf8	Zfp385b	Tlr5	Shc4	Mmel1	Rab27b	Catsperg1	Gemin5	Mapk1ip1l
Krt4	Actb	Has3	Cdk17	Gng3	Reps1	Best1	Kri1	Fbxl4
Msln	Abl2	Pgap2	Rab12	Ptk2b	Ints12	Kcnd1	Setd3	Pih1d2
Gm17532	Stk30	Nfkb2	Gtf2f1	Afmid	Rbm17	Tro	Pelo	Gsk3a
Sh3tc2	Pvr	Spns3	Fam118a	Xrn1	Dok1	E130116L18Rik	Tmod3	Zfp830
Tnni2	Gm17590	Trim46	Zscan20	Mtmr4	Creb3l2	Gm6404	4922501C03Rik	A630007B06Rik
4933428G20Rik	1110032A04Rik	0610010F05Rik	Rbfox2	Cul9	Tceb3	Efhc2	Ate1	Trappc2l
Hpcal4	Tnfrsf21	Fbxw4	Arl3	Ccnyl1	Rbm27	Krt39	Anapc10	Abhd15
Ahnak2	Crym	5730508B09Rik	Usp25	Zfp316	Ptbp1	Rspo4	D19Wsu162e	C230096C10Rik
Jub	Tmem43	Gm17541	Cpt1c	Wars	Golga3	Camp	Ccar1	Zfat
Gm9747	Slc38a3	Ppp1r12a	1700015E13Rik	Zfyve9	Tmtc3	Kcnj4	Thada	Nutf2-ps1
Ly6a	1810062G17Rik	Zfhx3	Cercam	Arvcf	Gm13138	Trcg1	Ubr5	Srprb
Ifit3	Gm14306	Podxl	3830408C21Rik	Cap1	Usp27x	Slc8a1	Wdsub1	Hsph1
Edn1	Tmem188	Mthfd2	4933426M11Rik	Pdcd6	Gbp9	Prss36	Gm5900	Dhx16
Arhgap23	Ptrh1	Epdr1	Iffo2	Cyp21a1	Tmtc1	Zfp40	Ufd1l	Metrn
A430105I19Rik	Efha2	Mtmr10	Obfc2a	Ccrl2	Plp1	Tbr1	Asna1	Lig3
Nmnat2	Rassf1	Trpv4	Enpp4	Dlg5	Hsdl1	Fcer1g	Srp14	Hsdl2
Hepacam	Chac1	Zfp951	2700007P21Rik	Pcbp4	Zfp280d	Col17a1	Amfr	Plod1
Abca4	Nek8	Ccdc109b	Ywhah	Krba1	Pls1	Pik3r6	Gmip	Cwf19l2
Isg15	Hspb9	Rcan3	Tusc3	Mapk4	Gpr146	Lilrb3	Whsc1l1	Dbt
Ifi44	Dusp13	Abi3	Atrn	Tpst1	Tmem181c-ps	Pcdhb8	L3mbtl3	Dcun1d4

Unc13d	Thsd7a	Spsb2	Myl6	Zfp948	Itpkc	Sema3f	Arap2	Bcr
I830012O16Rik	Ccdc64	Aspa	Helq	Mkl1	Ddx24	Ninj2	App	Mkrn2
Lama5	Arhgap21	Fads3	4732440D04Rik	AI846148	Slc37a3	Saa3	Ptges3	Zfp498
Hspb1	Kctd11	D930007J09Rik	Gm9754	Vmn1r53	39699	Gm4952	Stxbp2	Rhbdf2
Arhgap22	Trim6	Nab1	Tlk2	Gm3550	Max	Ucn2	Fam169a	Mpp2
Mmp11	Adm2	Tax1bp3	AI661453	Golga2	Rogdi	Izumo1	Rttn	6030429G01Rik
Ccdc80	Lhfpl4	Krt222	Fn3krp	Zfp771	Mst1r	Gm5533	Rangap1	Zfp438
Dntt	Krt1	Irx4	Ppfibp2	Itgb1	Prkcz	Cxcr3	2610027L16Rik	Med9
Mndal	Tubb2a	Tcea2	Gm5830	Tecpr2	Gm12666	Fezf1	Smcr8	Il13ra1
Khdc1c	AA986860	Mprip	Cars	Akt3	Lass5	Rassf9	Napb	Yipf2
Syt8	Rdm1	Nt5dc3	Pgam5	Ccdc24	Rsl24d1	H2-M10.6	Frmd6	Bcl7c
Syde1	Npff	Pkp4	Ccdc69	Khnyn	Zfp622	Ica1l	Gmeb2	Chst4
Vnn1	2010109I03Rik	Gm14322	Dynll1	Zdhhc21	Morf4l2	C1qtnf3	Tmem110	Dbf4
Mtap2	Pacsin2	Ncs1	Ube2g2	Mlkl	Syt6	Speer4c	Eif2b2	Eef1d
Cav2	Ehd1	Otub2	Sema4b	Zfp703	Gtpbp4	Gm5619	Trmt1	Ranbp2
Tinagl1	Diap3	Fhit	Trp53bp2	Krr1	Dok7	Cyp27b1	Kif1b	Trappc2
Plk2	Capn13	Epb4.1l5	Setx	Nup160	Shoc2	Igfbp2	Zfa	Stk25
Mras	Plekhg3	Myo5a	Arhgef37	Ybx1	Hoxb8	Dlg4	Cdc20	Camk1d
Thbs1	Msh5	Tacc2	1700092M07Rik	Parl	Gm10509	Dct	Maf	Lrp11
Cxcl10	Tnfrsf10b	Cltb	Etnk2	Tspan8	Lipt1	1110017D15Rik	Cc2d1a	Rnf25
Slc12a4	Strn3	5430407P10Rik	Tm2d1	Dot1l	Ptprh	Smok2a	Tnip1	Rpap2
Timp2	Ccdc112	Slc10a2	Tsc22d1	Mfsd9	Mapre2	1700027D21Rik	Dpy19l4	Tsen2
Serpinb9	Nudt17	Plcxd2	Dnajc17	Rock1	Ralb	Hist1h2ai	Dnahc17	Rnf41
Xaf1	H2-T24	Slc3a2	Unc45a	Ankrd27	Haus7	Tmem8b	Ccdc43	Klhdc1
Oas3	Trip6	Sema3b	Shisa5	Zkscan5	Comtd1	Fbxo2	Uxs1	Lrp5
Emid2	Sh3pxd2a	Rhpn2	Cdc42bpb	Tcp11l1	Pitpnm2	Fgf3	Arrdc1	Katnb1
Zfp37	Glis1	Dnajc5	Naglu	Ptp4a2	Hyal3	Ccr5	Vcpip1	Uck2
Atp11a	Sfta2	Clip1	Trim33	Ptdss1	Pck2	Kcnmb4	Syne2	Get4
Clca6	Rab39b	Chmp2b	Nfat5	Pafah1b2	Snx30	Adhfe1	Mycbp2	Paqr4
Bmp2	Zfp105	Apold1	Galnt3	O3far1	Lrch3	Ccdc146	Mier1	Kif16b
Ptrf	Enpp3	BC051019	1700102P08Rik	Rap1b	Chn1	Gm10174	Rsl1d1	Zfp282
Mup5	Lmna	Lta	Adss	Lmnb1	Dedd2	Prss22	Zfp689	Dym
Pak6	Casc1	Gm996	Kank1	Phf1	1500011H22Rik	Cdh9	Zfp180	Arid1b
Wfdc2	Trim34b	Aif1	Rela	Flot1	Ppp2ca	Hey1	Crybg3	Spg20
Ier3	Krt14	Casp14	Csrp1	Lrrc8d	Zfp691	Gm6531	Gpn2	Nolc1
Ddx60	Wls	Krt78	Pld2	Zbtb32	Efnb3	Hist2h2ac	Ifrd2	Gm8325
S100a3	Cdc42se2	Olfr1393	Snx24	Spry1	Paox	Lingo1	Rai12	Atp6v1d
Syt16	Tmem71	Lypd3	Wdr89	BC031353	Nap1l5	Smok2b	Ppp6c	Ubqln4
Rnf39	Gsn	Clec4a1	Pabpc1l	Pmel	Dvl3	Mageb3	Gm129	Prkaa2
Gjb4	Fam83h	Gm6498	Taf1b	Trip12	Dnaja4	Fam166b	Furin	Skiv2l
Akap12	Cbln3	A430110N23Rik	Bglap-rs1	Rps6ka5	Slc24a6	Gm1141	Vrk1	Dhdds
Calcb	Ppp1r13l	Ablim1	3110002H16Rik	Wbp4	Parp10	Slc35f4	Kidins220	Zfp110
Oas2	Fez2	Bcl3	Klc2	Wnk1	Gm5459	Pfn4	Puf60	Gm5611
Mx2	Oasl1	Zcchc10	Cttnbp2nl	Zwint	Cchcr1	Tnfrsf26	BC065397	Arl10
Gm16721	Rnf180	Samd4b	Myl12b	Hdgf	Itga5	Cnga3	Il7	Mrpl35
Phldb2	Adk	Hspa5	Gm347	Rab35	Xpot	C920008G01Rik	Trim66	Sdccag8
Apod	Trib3	Cep350	E130311K13Rik	Eef1e1	Rab7	Slc35f4	Vps35	Rrp36
Tm4sf1	Hpse	Ryk	Map2k3	Gm17436	Ddx41	Scarf2	Sdcbp	Med31
Enah	Rab34	Irf9	Ankrd56	Hist1h2bh	AU041133	Mlana	Mtmr11	Rabif
Stk32c	Cldn4	39701	Gda	Gm3222	Ncl	Hrasls5	Cldn3	Npepps
Rab40b	Gpr17	Prl2c3	Sh3gl1	Gm13145	Elmo1	Pcsk2	Rbm7	Slc20a2
Tgtp2	Aim1	1500003O03Rik	Clcn6	Gm13151	Cd7	Sucnr1	Triobp	Scaf4
Meis1	Greb1l	Large	Aldh7a1	Agxt2	Fam118b	Dnajc6	BC016579	Atg4a
Irs1	Thsd4	Inpp1	Cyp20a1	Unc79	Hipk2	Mei4	Ppp4r2	Gm5848
Ano1	Mycn	Serpinb1a	Wdr60	Ddr2	Sgms2	Nfatc4	Nhlrc2	Gm9869
Aass	Tpst2	Wsb2	Unc119	Acyp2	Sms	Col4a3	Heatr1	Tbck
Tead4	Ctsl	Capn12	Ptprr	Rps2-ps6	Cdc14a	Trim44	Kpna1	Snx19
Dok2	Slc17a8	Tctex1d2	Parp4	Ccl24	Phc2	Hsd17b1	Aptx	Parn
Epha2	Krt23	Pgap1	Btbd10	Chd5	D19Bwg1357e	Olfr658	Tmem98	Pnn
A930004D18Rik	Baiap2	Cd46	Ndel1	Cnr2	Ddx21	Gm9933	Eftud1	Pop7
Ahnak	F2rl1	Fam188a	Wasl	Cyth4	Blzf1	Emr1	Thop1	Cul3
Anxa3	1700019B03Rik	BC006779	Phgdh	Tek	Parp9	Gm6289	Prpf6	1600012H06Rik
Myof	1700013N18Rik	C530008M17Rik	Cpvl	Gm13151	Prep	Wnt10a	Vsig10	Cops2
Klrg2	Zfp651	BC029214	Zcchc24	Prr18	Pla2g4b	Prom2	Otub1	Rtel1
Klhl13	Pacsin3	Rps4y2	Dmkn	Otoa	Yars	Tmem212	C030046I01Rik	Trpc4ap
Spon1	1700001J11Rik	1700034J05Rik	Dpf1	Fndc4	Fam49b	Alox12b	Ercc3	Rhbdl2
Ptpn14	Gm3002	Klc1	Otog	1700012A16Rik	Pkd1l3	Tie1	8430410K20Rik	Mid1ip1
Nid1	Irx2	Med13	Grid2ip	2410004B18Rik	Pppde1	Cyp2j13	Napg	Lsm12
Atp6v0a4	Nanp	Snta1	Adprhl1	Itpk1	Ccdc41	Sds	Plxna3	Pdcd7
Cdkn1c	Vnn3	Gnai1	Polr2k	Elac2	Eif4h	Gm6316	Ifi27l2b	Galns
Creb5	Cd59b	Hist1h2ae	Hoxb5	2310021P13Rik	Invs	Gm8975	Pcbp1	Zfp598
Tgm1	E230008N13Rik	Dzip1	Clec11a	Stk3	Ghdc	4930579C15Rik	Gm10621	Dffb
Capn2	Hist2h2bb	Sycn	Apol7b	Rrp12	Mbtps2	Synpo2	Stk36	Mocos
Bdnf	Rpl26-ps2	Chn2	Rtn1	Nop58	Psmc3	4930443G12Rik	Tle3	Cadm1
Pnlip	Acoxl	Ifi47	1700016C15Rik	Iqgap1	Gm3436	Tpsab1	Ell2	Dhx30
Enkur	9130014G24Rik	Trpc1	Rab21	Llgl2	Il17ra	Gm10916	Itgb1bp1	Gltscr2
Dlk2	Olfm2	Npc1	Hk2	Psmd14	Htatip2	Gm5145	Vmn2r4	Nebl
Zbp1	Gm10414	BC025446	Myc	Ubc	Gm6190	Gm4983	Vmn2r2	Arpc1a
Stx11	Foxd3	Dusp4	Cdc42ep1	Slc5a9	Ube2j1	Hist1h1t	Gm9479	Smarca4
Dscam	Gm8898	Ifih1	Tor3a	Sp2	Lsg1	Fam71e1	Vmn2r1	Fam189b
Pmp22	Vmn2r29	Foxa1	Gys1	Dos	Slc9a1	Oas1d	Agpat1	Agl
Apol8	2810055G20Rik	Plekhf2	Ap2m1	Slc25a14	Arpc4	Gm10343	Ubap1	Zfp579
Nt5c1a	Cxcr4	Fgfr3	Nmt2	Sars	Cux1	4930579K19Rik	Katna1	Slmo2
Rin3	Col4a4	Ppm1k	Rassf7	Psap	Wwp2	Gm17658	Slc35d2	Hist2h3c1
Ifit1	Lmtk3	Tesk2	Dnd1	Rbbp8	Cdk5r1	Psg23	Gaa	1810008A18Rik
Slfn5	Slfn2	Tulp3	Sys1	Tgif1	Hsp90b1	Gm5321	Mbnl1	Slc29a3
Rtp4	Kazn	Ptprj	Stard13	Fkrp	Dfna5	Vwa3b	Naa15	Shc1
Kbtbd10	Cwh43	Pcx	Ctif	Fam46a	Lancl2	Guca1b	Amdhd2	Plekhj1
1700056E22Rik	Anxa5	Cep290	Maml1	Urgcp	Dda1	Hist1h2bn	Zc3h10	Tctn2
Anxa1	Prkx	Rilpl1	Rab17	Fam53c	Eid1	Tac4	2310003F16Rik	Plod3
Aloxe3	Mospd2	Cxcl9	Heatr2	Aldh1l2	Cct6a	Rasgrf1	Mapkapk3	Mapre1

Lats2	Tmem62	Tekt5	Cast	Copa	2010007H12Rik	Il21r	Zfp111	Tdp1
Ptchd3	Krt19	Wdr78	Abtb2	Npdc1	Ap1b1	Lin7b	2210411K11Rik	Crebzf
Gjb5	Scnn1a	Pnma1	Nras	Trak2	Efcab7	Calcrl	Epsti1	1700020O03Rik
Gm2783	Prkci	Krt77	Trim3	Zc3h11a	Stam2	Kbtbd12	Lamtor3	Fam105b
Crip2	Gprin1	Gpd2	Ston2	Spag1	Atxn7l1	Arxes2	BC016423	Kif3c
Arhgap40	Akr1b8	Fam129b	Zbtb4	Kif23	Ccdc126	4921515J06Rik	Mtfr1	Pick1
D14Ertd668e	Myo1e	Srp54a	Lonrf3	Ptger1	Chmp4b	Med12l	Rpap3	Asb3
Syt14	Areg	Ptpn9	Dnahc10	Cno	Dync2h1	Ovol3	Cdkal1	Mga
Slc7a3	Krt16	Notch2	Slc6a9	Senp2	Ppp1r9b	Slfnl1	Atg4b	AI316807
Tmem151a	Tnfrsf22	Gm11067	Foxj3	Synj2bp	Zfp236	Nkx1-2	Ankrd42	Nkain1
Fabp3	Krt9	Sqrdl	Arhgap1	Kdm6b	Myo19	Ppp1r1a	Pitpna	Cnih2
Stbd1	9930012K11Rik	Midn	Tnfsf15	Uba3	Nlgn3	Ms4a6b	Nae1	Mkx
Dynlt1e	Krt8	Rhbdd1	D730040F13Rik	Sdf2l1	4930555I21Rik	Rasl10a	Phf20l1	Zfp367
4931428L18Rik	Krt17	Socs5	Rrp9	Ddx39	Phax	Wscd1	Rpl24	Gsdmc2
Lgsn	Dusp8	Lipo2	Tjp2	Maea	Trim30d	Apol10b	Wdr46	Snca
Plaur	Pdp1	Adcy9	Syn3	Tbc1d24	Trmt12	Gm1337	Ccndbp1	Mr1
Krt7	Rasl11b	Aak1	Tbc1d10c	Braf	Zfp281	Myl9	Atpbd4	Dsel
Pcolce	Agpat4	Slc29a2	Dgke	Arfgap1	Pax8	B3gnt8	2810407C02Rik	Gm9775
Jdp2	Loxl2	Htra3	Zfp36	AI413582	Znrd1	Pkia	H2-D1	Gramd2
Clu	Far1	Adrb2	Pfn1	Fchsd1	Lphn3	Gmpr	Zfp746	Ube2e1
Dnm3	Fam38a	Dmrta2	Nenf	Itfg2	Zbtb25	Dip2a	Rrp15	Bod1l
Tnnt2	Pmepa1	Inadl	Tmigd1	Hgsnat	Spen	Gm14391	Gba	Ppm1g
Hdac7	Ap3m2	Rasgrf2	Vps37b	Unkl	Zc3h12a	Dlgap3	Trim40	Rps26
Rbm24	Lrrc8e	Asb1	Dnajc21	Dock5	Nop2	Srpk3	B3galt6	Fbxw2
Gm10972	Sfxn3	Spire2	Ankrd43	4930442H23Rik	Gabpb1	Tgm5	Rnf8	Capn1
Fosl1	Zfp568	Slc8a3	Gm17227	Gm9970	Mina	Ticam2	AI848100	Hyal2
Sorbs2	Gm15319	Tmtc2	Hspa2	Mtmr6	Klc3	Gm8226	Atp6v0d1	Cops7a
Dzip1l	Stx2	3110070M22Rik	Trim35	Enoph1	Sbf2	Siglece	Rb1cc1	BC046331
Rnd1	Fbxw17	Dsg2	Tmc7	39692	Reps2	Tmem28	Mtmr12	Polr3f
Ildr1	Ezr	Usp54	Lrrfip2	Plscr2	Tra2a	Gm17352	Wdr65	Sptlc2
Cnn2	5730528L13Rik	Bbs4	9930013L23Rik	Spata1	Gm9781	2410124H12Rik	Gm6139	Plekha3
Glis3	Slc31a2	Ctnna1	Gimap6	Psmd4	Fam50a	Tmem59l	4933437F05Rik	Cdc37
Sp100	Gadd45a	Ascc3	Efna2	Cables1	Scaf1	Cyfip2	5033411D12Rik	Trex1
Oasl2	Runx1	Kitl	Drd3	Lrrc59	Prps1	2810055G20Rik	Dynlt1f	Zbtb22
Nrip3	Celf2	Lpcat4	Gdap2	Nsfl1c	Sh3bp5l	Gm10135	Hspb6	Akp3
Plat	Susd1	Taok3	Ets2	Mppe1	Senp5	Dnahc6	Pphln1	Poglut1
Gm5431	Pip5k1a	Mxd1	Sipa1l3	Gm17305	Usp9x	Magix	Tbcd	Celf1
Camk1	Tmem140	Thbs3	E430025E21Rik	Atf7ip2	Smc5	Glra4	C330006K01Rik	Ankrd11
Dnahc2	Shb	Ptprcap	Gpr160	Mical3	Fmnl3	Ube2s	Psmd6	Secisbp2
Csrnp1	C330046G03Rik	Hist3h2ba	Nsmce2	Brf2	Fmnl1	Mlf1	Ccdc17	Rnf14
Ttll13	Dusp6	Cspg5	Ldlr	6330408A02Rik	0610010O12Rik	Itgal	Abhd8	Tmem104
Serpinb6c	Pitpnm3	Mical3	A830080D01Rik	Tmem9	Ticam1	Cdh4	Nup88	Ubtd1
Camk1g	Pim3	Gm9938	Rit1	Gm17345	Atf6	Lyl1	Fhdc1	Aip
Samd4	Ddx58	Usp11	Hyal1	Cep63	Mall	Gm17651	Psen1	Hs6st1
Pld4	Mtap4	Fam84a	Stx7	Sec24b	Mta3	Golgb1	Cmtm7	Intu
Dusp1	Fam160a1	Fam69b	Tmbim1	Gm9182	Fam126a	Cetn4	Dph2	Ptprf
Myo1c	2900073G15Rik	Sult2b1	Oxsr1	Hoxb3	Noc4l	Gm16485	AI987944	D030056L22Rik
Ptprb	Gpt2	Nyx	P2rx3	Wdr81	Pcid2	Qrfp	Apba3	Lysmd3
Bcam	Rdh16	Wdr35	Spata9	Manba	Cwc25	Mpeg1	Gpx3	Bok
Zfp57	Gng2	A230050P20Rik	Grlf1	B3gat2	Slc9a6	Fam194a	Tceb2	Zdhhc1
Ifi203	Krt42	Gars	Fbxw8	Wiz	Bcl7b	Gpr4	Dmxl2	Me1
Ppp1r2	Anxa8	Fgfr4	Usp31	Slc30a4	Rnf166	BC051070	3110001I22Rik	Chd2
Rnf208	Agrn	Rnh1	Ttc26	Snx2	Dnajc27	Naa25	Taf7	Skiv2l2
Cd22	Plcd3	Cyp2w1	Prune	Usp24	BC031781	Gtf2h1	Ftl1	Ascc2
Ankrd29	Psg25	Clmn	Mab21l2	Smc6	Srcrb4d	Wdr45l	Dtx2	Utp14a
Slc35e4	Lypd6	BC048546	Myh10	Nfe2l1	3110009E18Rik	Nup98	Nck2	Ankrd39
Gprc5a	1700012L04Rik	Nedd4l	Chst11	Bcl10	Cxcr6	Gcn1l1	Lzts2	Rom1
Emp1	Als2cr4	Plek2	Psph	Bcat2	Rel	Myst3	BY080835	Smg5
Tceal7	Slc22a23	Nipa1	Phf19	Ube2o	Psd	Npas2	Mblac2	Rest
Gm17359	Pdzk1	Gm11175	Mad2l1bp	Spin4	Ltv1	Ncstn	E130309D02Rik	Ppp3ca
Fosb	Camkk1	Socs6	Atp6v1b2	Foxred2	Prkd2	D230037D09Rik	Dennd1b	Madd
Cish	Ptar1	Il4i1	Purg	Csda	D19Ertd737e	Sntb2	Supt5h	Camk2g
Abcc5	Ttll1	Prss57	Slc7a6	3110082I17Rik	Zfp961	Pwp1	Gpbp1	Ppp1cb
Nt5e	Tspan9	Csf2ra	Tmeff1	Phf10	Tada2a	Cep135	Mier2	Naa50
Ptprt	Gm10463	Rdh10	Sipa1	Asb6	Htr2b	Cldn7	Pop1	Prmt1
Basp1	Pcyt1a	Cldn25	Lars	Plxna2	Fam111a	Eral1	Ppp1r10	Nckap5
Tgm7	Tln1	1110007C09Rik	Gm5819	Psmc4	Nanos1	9430023L20Rik	Cct3	Srp72
Chrnb1	Sqstm1	Ubash3b	Gm17617	1700019E19Rik	Ilk	Mpzl2	Trim43c	Dyrk1a
2310007B03Rik	2810474O19Rik	Xiap	Hspb8	Clcn7	Adrbk1	Zfp609	Tmod1	Gm8994
Hspa1a	Fam131c	Adcy4	Gm6899	Tmem116	Lass2	Actr10	Pcbp2	Parg
Fn1	Eng	Pycr1	Oas1c	2310036O22Rik	Abcf2	Tubb2c	Zc3h18	Plin3
Atp2b2	Gm5481	Mfsd7c	Gm4477	Ankrd40	BC048355	Lats1	Klhl2	Dnajb5
Dscaml1	Gm17511	Rfx2	1700029J11Rik	Tmco1	Zfp939	Mak16	Phf8	Arpp19
Gsg1	Nbea	Cd97	C230062I16Rik	Sox6	Vapa	Gm16286	Ribc1	9130219A07Rik
Foxn1	Cdipt	Nbeal2	2900092E17Rik	Xpnpep2	Dusp12	Ncoa6	Bmi1	Suv420h1
Lin7a	Pgap1	Slc39a13	Slc38a2	Alkbh3	Zfp276	Tgfbrap1	Wapal	Ccdc71
Glis2	1810048J11Rik	Slc26a11	Ctps	Gm5908	Frs2	Abt1	Frg1	Rhobtb2
Ripply3	Spnb5	Map3k3	Pkd2l2	C130050O18Rik	Gm2178	Slc48a1	Cdc40	2010012O05Rik
Gm17244	Mrc1	Nefh	Uhrf1bp1l	Atp6v0c	Abcg1	Kalrn	Tcp1	Adnp2
Ptgs2	Serpinh1	Pvrl2	Nek7	Cyfip1	Btbd7	Mmadhc	Dnmt3b	Trf
Flna	Lepre1	0610007L01Rik	Tatdn1	Pak4	Il18bp	Polm	Fmn1	Zfand3
2310002L13Rik	Dusp8	Limk1	BC046404	Capzb	Ubap2	Znrf1	Arih1	Hoxb7
Bex1	Uap1l1	Tanc1	Nfatc1	2310057M21Rik	Ckap4	Ccnd2	Ranbp3	Pex5l
Aqp9	Lpcat2	Tceal8	Hsf2	Arl13b	Nop56	Gopc	1810063B07Rik	Myg1
Npnt	Itgax	Zfp772	AA960436	Lrrk1	Tcof1	Lman2l	Psma1	Morc3
Runx2	Vpreb1	Creb3	Ogfr	Pbx1	Tmed5	Ptgfrn	Ndnl2	Stxbp4
Mmp23	Slc25a4	Glt8d2	Fam102b	Acbd3	Bcl9l	Riok2	Marveld3	Eif4enif1
Lamc2	Crim1	Ttc7b	Cln5	Kdm1a	Odz3	Vps72	Nr4a2	Pspc1
Mfap3l	Fam107b	Rnd3	Clasrp	Reep3	Sh2b2	E2f4	Rpl12	Zfp341
Kcna1	Qk	Dcbld2	Cul4b	Nub1	Zmat4	Rpl35	Rrp7a	Gatad1
Dpp6	Sbno2	Zan	Gm9742	Arap3	Chrd	Sav1	Tmem115	Dhx40

Asprv1	Pparg	Map4k5	Mat1a	Csf2rb2	Herc4	Ppm1b	Sel1l	Tubb6
Dse	Sash1	Impact	Srpk2	B930041F14Rik	Shroom2	Traf3ip1	Sos2	Prpf8
S100a14	Renbp	Parp14	Zfp36l1	5033430I15Rik	Lactb	Kdelr3	Tceanc	Rfc5
Amotl1	Sh3rf2	Ak7	Lpcat1	Rps13-ps1	Mtr	Tmem170	Zfp945	Zfp335
Pfn2	Coq10b	B230312A22Rik	Cyb5r1	Mib2	Hsp90ab1	Cobl	Nudt9	Isy1
Pla2g7	3830403N18Rik	Rabgef1	Cobll1	Slc9a7	B630005N14Rik	Trove2	Uba2	Imp4
4933427E11Rik	Zbtb16	Pdcd6ip	Fam122a	Hsf3	Mid1	Txnl1	Commd10	Ubl4
Yjefn3	Pcp4	Lmo4	Ppp2r2a	Cdc34-ps	B020018G12Rik	Pgs1	Zscan29	Asap2
Sprr2h	Gna14	Gm5453	Rhou	Polh	Gm6907	Josd2	Ppfia1	Enc1
Sema3c	4732456N10Rik	Vps13a	Klf9	Trappc6b	Ethe1	Mpp6	Abcf3	Akap11
Ppp1r9a	Kif17	Avil	Irgm1	Mapkapk2	Cct4	Cct5	Xpo4	C330007P06Rik
Fam55c	Arl5c	Amz1	Eif1a	Lgals8	6430598A04Rik	Ddx3x	Polr1e	Mesdc2
Lamb2	Gm6658	Jmjd1c	Adora1	Psmd7	2610528E23Rik	Foxn2	Atp6v0e	Gmpr2
Tmprss11f	Olfml3	Hsd3b2	Cyb5d2	Jmjd1c	Upf3a	Prr3	Kdm5b	Map3k7
Gngt2	1700011A15Rik	1700001C19Rik	Nsmaf	Mfsd5	Fkbp15	Zfp566	Wdr19	Bloc1s3
Rab11fip5	Cabyr	Errfi1	Leprel4	Utp23	Polr3a	Jak1	Gga1	Gpx4
Slc24a3	Coch	Hsp90aa1	Tarsl2	Ap3m1	Limk2	Mfsd6	Tmem87a	Pram1
Slc35d3	Zfp583	Spr-ps1	Rnf11	4930503L19Rik	Rab11a	H2-M3	Bzw1	Shq1
Col9a3	Rbpjl	Xlr3b	Jup	Gm9982	Kctd20	Sirt6	Ap4s1	Rfk
Gm3200	Rnase6	Ogg1	Ocln	Bre	2210408I21Rik	C030048B08Rik	Pinx1	Drosha
Scara3	Kcnrg	Dock11	Cyth2	Rnaseh1	Rrs1	Akr1b3	Lonp1	Snapin
Ndrg4	Hist2h2aa1	Osbpl1a	Spnb3	Aatf	Dnttip2	Pprc1	Mipol1	Jkamp
Igtp	Ptprq	Rock2	Cdc42ep5	Tbc1d19	Iqsec1	Rnf24	Dstyk	Asxl1
Prss23	Fam65c	Dbndd2	Slc44a1	Fam72a	Dscr3	Ubl3	Pes1	Psen2
1700019G06Rik	S100z	Snap47	Klf16	Ccdc109a	Map4k1	Nr1h4	Bop1	Hmg1l1
Lgals3	Klhdc7a	Hspbap1	Mybl2	Cyp3a13	D830039M14Rik	Liph	Dnajc24	Klf10
Syt12	Svep1	Cln8	H6pd	Fkbp7	Gnaq	Sra1	Gng5	Plekho1
Lhfpl2	Anxa2	Cdr2l	Rcn2	Sorbs3	Dgcr14	Gnb2	Tctex1d4	Nat10
Scrn1	Trim15	Tns3	Cxadr	0610007P22Rik	Polr1c	Cherp	Zfp30	Tesk1
Gm88	Suox	Hpgd	Eprs	Tpm3	Bak1	Apob	Hsf5	Prmt3
Lman1l	Atxn10	Magi3	2210404O09Rik	2310042D19Rik	Itm2c	3230401D17Rik	Col18a1	Gns
Gm17579	Abcg2	Itpkb	Anxa9	Macc1	Hspbp1	Add1	Gm17669	Pogk
Adamtsl4	Dusp5	Kctd3	Tmem150b	Srp54c	Athl1	1700020L24Rik	Hist1h1a	Tcerg1
Dpcr1	Epb4.1l1	Mtap	Cd274	Hint3	2810006K23Rik	Cox18	Hist1h2be	Cfl1
Atp2a1	Gas8	Eno3	Chst14	Phf13	Chordc1	Rpl15-ps2	Six4	Cwc27
Slc5a6	Cyp4b1	Hist1h1d	Daxx	Arhgap19	Rabggtb	Eif4g3	Proc	Cklf
Atf3	Rims3	Arl6ip5	Dtx3	Lipo1	Ppp2r2d	Abi2	Gm13199	Uchl3
Ltbp4	Sned1	Avpi1	Kctd13	Wrnip1	Maz	Golt1b	Dcdc2b	Scamp4
9430020K01Rik	Ereg	Mpzl3	Rab25	Gm9874	Ptprm	Lamp3	Hnrnpul1	Zbtb7a
Arhgap29	Eml2	Car13	Dapk3	Fut1	Rcor3	Gar1	Brpf3	Inpp5f
Pcdh17	4921536K21Rik	Ppap2b	Mitd1	6330545A04Rik	Gm5045	H2-Q4	Rbm39	Wdr55
Gpr115	Ccdc162	Dgka	Gm17374	Sox21	Wdr25	Cks1b	Fbxl8	Nckipsd
Frmd7	Pde6g	Jund	Kif9	Gyg	Sec23b	1110049F12Rik	Orc4	Pik3r4
Msrb3	Ntn3	Nradd	Hemk1	Gnl2	Rpp25	Elavl1	Gorasp2	Farsa
Oxct2b	Sgtb	Sema6a	Tgfa	Mphosph6	Tubgcp2	Klraq1	Itgb5	Xpc
Kifc3	Nxf7	Glipr2	Siva1	Cc2d1b	Rabac1	Tuba4a	Gm2792	Aph1b
Hspa1b	Psat1	4930455F23Rik	Col20a1	Rhog	Paf1	Med19	Gm9762	Ipo9
Smtnl2	Serpinb5	Srgap2	Mef2d	Shmt2	Lemd2	Tmem189	Ccnl1	Esrp2
Cntnap2	Rap2b	Eps8l2	Ccdc50	Med26	Gm5577	Vta1	Zfp408	Gm7535
Arl4c	Slc16a8	Pml	Cdo1	Ube2z	Nkx6-2	Cct2	Tmem139	Arfip1
Ccdc68	Maml2	Specc1	Tmem158	Dhps	Cdv3	Nufip2	Nin	Ier2
Dusp9	Bmp8a	Trim13	Gm10462	D330045A20Rik	Ssu72	Nol12	Zzef1	N4bp3
Wwtr1	B430305J03Rik	1300014I06Rik	1110034B05Rik	Rnf19b	Gm2833	Wdr75	Aen	Ccdc46
Glis3	Cyp1a1	Slc9a3r1	Gab2	Spata7	Dynlt1c	Pdcd11	Taf1d	Gm10345
Nhedc2	Ctnnal1	Coro1c	Tmsb4x	Tars	Ccdc106	Klf15	Itga6	Ywhab
Trim69	Cd160	Stoml1	Vasp	Arntl	B4galt3	Acat3	Rps7	Moap1
2310002J15Rik	Catsper4	Kremen2	Mcpt2	Senp6	Hcfc2	Kpna6	Anapc4	Hnrnpul2
Xkr4	Hsd17b2	Ifngr1	Ggn	Mbnl2	Aimp2	Nnat	Ccdc124	Klhl7
Epb4.1l4a	Cbfa2t3	Capg	Yrdc	Ncdn	Eif4e2	Krt6a	Zfp639	Rbmx2
Id2	Gm527	Dbndd2	Rab32	Rrm2b	Snx18	Sf3a2	Hnrnpc	Glud1
Tnfrsf12a	2310028H24Rik	Gm10518	St14	C1qtnf1	Dus2l	Ube2e2	Prmt5	Mpp7
Gnat1	Stk10	Eif5a2	Dync1i2	Bcl2l13	Ccdc86	Fbxl15	Mark2	Scaf8
Arhgef17	Tspyl3	Stat1	Tmem120b	Ubr4	Pkd2	Nlgn2	Adck4	Gtf3c5
Pdzk1ip1	Sult4a1	Rtn4	Mapre3	Dnajb1	Plk3	Cd72	Npm1	Exoc7
Ston1	Rasip1	Bach2	Gm6583	Anapc1	Nus1	Hmgn1	Gm4885	Mfsd2a
Pdlim2	BC048507	Ube2l6	Zfp553	Secisbp2l	Selk	5730559C18Rik	2610002J02Rik	Fbxo11
Fam129a	Glt28d2	Rgs19	Gcnt2	Ppp2r1b	Dnajb9	Mfn1	Trim41	Tmem107
Slc6a14	Ap1s2	Mgat4c	Rac3	Dmap1	Zfp407	Il22ra1	Ifi35	Mrpl22
Trex2	Dsp	Clcn5	Gzmm	Nlrx1	Gm17415	Foxk2	Hsf1	Tssc1
Ptpn13	Nap1l3	Mtm1	Ccdc163	Cdc42bpa	Stk19	Mphosph8	Fubp1	Dnpep
Fbxo24	Ift81	Frat2	Gpatch1	Rasa2	Fam108c	Eif3b	Pak1ip1	Zcchc3
Nr4a1	Grm4	Zfp189	Ulk3	Scyl2	Eif2c2	Ppip5k2	Nexn	Stard3nl
Jag2	Sdcbp2	Kctd7	1700017B05Rik	Ficd	Sun2	Ddx23	Chmp7	6530409C15Rik
Sprr2a2	Fam101b	4930523C07Rik	Ppp3cc	Agphd1	Relt	Nprl3	Ddx19b	Gm3150
Klk10	Cacnb3	Rab31	Lbh	Tprgl	E130114P18Rik	Tox4	4930506M07Rik	Sumf2
Cdhr1	AW011738	4921524J17Rik	Ippk	Rhoc	Mfap4	Casz1	Acsm3	Pnliprp2
Gm17237	Tor1aip1	Pak2	Ube2f	Srrd	Hcn4	Ap4e1	Pin1	Rin2
Ryr1	Mum1l1	Mthfr	Grin3b	Ubr1	Ube2m	Heatr3	Rgnef	Ltn1
Tmem136	Gm10657	Adap1	Atf7	Evi5l	Zfp597	Hyou1	Ythdc2	Dusp19
Fgf13	Abhd12	Dok4	Gab1	Kif3b	Larp1	Plcxd1	Chmp5	Slc25a32
Epn3	Camsap2	Tes	Ecel1	Gdf11	Trim27	Vps37c	4930403N07Rik	Snip1
Rusc2	Ttc22	Asah1	Flt3l	Nup54	Slc18a1	Prrc2b	Aggf1	Qtrt1
Hecw2	Pbx4	Stat2	Nploc4	Rap1gds1	Zfp26	Rnf10	Gm10093	Hdgfrp2
Gbp7	Gsdmd	Abcc3	Gcom1	Fnip2	Opn3	Eif4e	Fus	Mbd5
S1pr2	Usp46	Muc1	Mical2	Pwp2	Arpc5	Gm6457	Casc4	Rabepk
Omp	Egr1	B230216G23Rik	Depdc7	Hprt	Gm6505	Ppapdc1b	Vgll4	Ccne2
Ppp1r15a	Pomc	Zp1	Fndc3b	Rap2c	Ncor2	Tmem194	Phf20	Gng10
Ttc9	1700084J12Rik	Efhb	Cstb	Rlim	Stk40	Gm6807	Vps54	Dusp11
Sh3bp5	Tbx3	Tuba3a	Zc4h2	Rab11fip4	Rarg	Foxo1	Lgals4	Blmh
Prkag3	Esyt2	Pla2g4f	Erich1	D830046C22Rik	BC005764	Gnb1	Ddx19a	4933427D14Rik
Apon	Stx1a	Zfp354c	2210010C17Rik	Letm2	Rrn3	Ube2v1	Dyrk3	Tmem209

Hephl1	Ccdc164	Asb14	Tbrg1	Ick	Trim62	Gm9905	Denr	Mrfap1
Gbp2	S100a16	Ccdc37	Tmem198	Pkn2	Usp16	Zfp7	Gp1ba	Gps1
Cd55	Gm7334	Serpina1e	Mapkbp1	Zfp599	Wdr43	Mmgt2	Plcd4	Zfp384
Id1	Mmp14	Gm7932	Phka1	Lpp	Znrf2	Atxn1	Dab2ip	Tiprl
Nkpd1	Slc25a24	Upk1a	Sesn2	Alg12	Sugp1	Ets1	Urb2	Raet1c
Cdc42ep3	Plekhm3	Pcdhb15	8430410A17Rik	G630016D24Rik	Atg3	Gtf2b	Tasp1	Tubb3
Rras2	Cyb5r2	4930428E23Rik	Pacs2	Cops8	Ddx18	Ecscr	Lmtk2	Snx32
Dhx58	Fetub	Gsdmc4	Irs2	Lyar	Bdp1	Lrrc49	Ints8	1700030C10Rik
Tceal1	Plcd1	Gm5414	Pdlim1	Cdk11b	Gstcd	Yif1b	Wbp2	Gm10369
Nhlh1	Smpdl3a	Gm10731	Gm17687	Brd2	Grin3a	Nob1	Tbc1d9b	Tspyl5
Trim30b	Il34	Hist1h2ac	Adrm1	Eif2c4	Fam33a	Oscp1	Nol8	Gm9008
Fbxw10	Rhob	Sh2d4b	Socs7	Rasgrp1	Ctdspl2	Camkk2	Zfp865	Nle1
Sprr2d	Mafk	Atp5l-ps1	Plscr3	Lsp1	Crtc3	4933440N22Rik	Rhot1	2310022A10Rik
Rnf151	2810008M24Rik	Ccdc103	Zmpste24	Gm10322	Psmc6	Dffa	Kbtbd2	Eif4a1
Btn2a2	Sertad1	BC055111	1600027N09Rik	Hist1h2ag	Fbxo31	Nupl1	Zfp229	Mcts2
Trim30b	Gm10604	Vma21-ps	Rbm18	Adcy10	Loxl3	Ythdf1	Slc37a1	2410001C21Rik
St3gal2	Kif5a	Arhgef15	Acvr1	Krt71	Ube2h	Ranbp1	Fkbp14	Adprhl2
Fam189a2	Cdh11	Rpl30-ps8	Glce	Barhl1	Plb1	Zdhhc12	Cetn3	Dlgap5
9930111J21Rik1	Zfp248	Car11	Nt5c3l	Rsph1	Actr3	Prkaa1	Prkab2	Wdr59
Kank2	Cfl2	Arhgef33	1810013L24Rik	Rgl3	Mns1	1110057K04Rik	Nkx6-3	Hs1bp3
Slc25a48	Abcc1	Ranbp17	Supt3h	Msl3l2	Slc7a7	Memo1	Nek6	9530068E07Rik
Lamb3	Dennd5a	Pmfbp1	Pik3c2a	Pgc	Elk1	Fbxo30	Ccnc	Cpsf2
Tgfb2	Cgn	Gm12033	Gm10300	Tmprss9	Phf23	Stxbp3a	Arf1	Coq4
Pcsk6	Gm6096	Amy1	Zswim4	Rhbdl1	Phtf2	Ammecr1l	Ipo7	A130010J15Rik
Ppp1r3d	Bst2	Crtam	D4Bwg0951e	Rasgef1a	Tor2a	Ikbkap	Atad2b	Mocs3
Hs6st2	2010109K11Rik	Apbb1	Grk4	Slc25a18	Sar1a	Ptplad2	Pfdn2	Cbwd1
Slamf9	Zdhhc8	Spink5	Rab33b	Prkrip1	H13	Ubap2l	Smyd5	Fam76a
Clic4	Il3ra	Tat	Lancl1	4921523A10Rik	Uchl4	Eif4a3	Srcap	Rnpc3
Sertad4	Itpripl2	Mmrn2	Trp53rk	Dhx38	Adat2	Vdac3	Tk2	Setdb2
Gadd45b	Plekhg2	4932412H11Rik	Osbpl11	Fam65b	Adh7	Crkl	Ankib1	Nat6
9930023K05Rik	Cdon	Qrich2	Abhd4	Ankrd28	St7	Phrf1	Rpl27	Arcn1
Id3	Vstm2l	Gm8112	Pnp	Tmem106a	Ccdc9	Cdc42bpg	Cdx2	Rab2a
Prnd	Agps	Calca	Mex3c	Efcab5	E2f7	Scaf11	Psmb5	Mki67ip
Cgnl1	Tns4	Mapk15	Ift122	Otud5	Thumpd1	Zc3h7b	Zxdb	Sirt1
Bmp8b	F11r	Arhgef9	Mllt6	Appbp2	Gm10340	Aifm2	Cdh17	Dnase1l1
Tuft1	Ddhd1	Snai1	Pold4	Ap3d1	Cct8	Foxj2	1200011M11Rik	Chtf18
Sprr2a1	Junb	Lef1	Trim45	Pkn1	Rab11fip1	Psmb4	Txndc9	Rad9
Sprr2b	Myh14	Ptpdc1	Tbc1d15	Rragc	Gm3086	Sike1	Sf3a1	Dync2li1
Cidec	Pdlim7	4933405L10Rik	Atxn1l	Zfp280c	Ppp1r12c	Shank2	Abca2	4632434I11Rik
Rrad	Gdf15	Gm2237	Zcchc11	Sdccag3	Rpf2	Dhx33	D19Ertd386e	Ap2a1
Slc43a3	Gas2l3	Gast	Apaf1	Igf2bp2	Ssfa2	Dip2c	Hspa4	Ric3
Lynx1	Afap1	Gm2026	Rsph9	Bysl	Med7	Thap3	Fkbp5	Vps11
Klf4	Mmp24	Gm10313	Wasf2	Timm17a	Tuba1c	Clk3	Plekha1	Stx16
Gna15	Gm98	Acot5	Creld2	Fam171a1	Plekhh1	Lrrcc1	Zfp623	9930021J03Rik
Tmem52	Slc4a11	H2-Eb1	Atp2a2	Dpp9	Stxbp3b	Ppp1cc	Rsu1	Uimc1
Mdfic	Syt17	Cntfr	Cntnap1	Fam166a	Zfp503	Kdm4c	Tmf1	B930036N10Rik
Il18rap	Mal	Hoxb2	Stam	Ebna1bp2	Tirap	Rraga	Unc13b	Syvn1
Abca12	4930402H24Rik	Irak1bp1	Ptpre	Sertad2	Mier2	Wdr62	Mea1	E2f5
Pim1	Pias3	Gm17527	Vapb	Dcaf13	Qsox1	Rnf6	Mtf1	Cebpg
Itgb6	Tmem125	Lass3	Pfdn1	Hps4	Hspa4l	2510003E04Rik	Myd88	Lztr1
Klf6	Mark4	Xkr6	Ppp4r1	Wnt4	Fxr1	Bmpr2	Heph	Zmynd17
Pard6b	St3gal6	Myo16	Slc22a15	Pdcd5	Klf13	Nt5dc1	Fam92a	Nop16
Prss27	Arhgap8	E230025N22Rik	Gckr	2410042D21Rik	Noc3l	Nid2	Dhx15	Snrnp48
Ntng2	Arhgef18	Hist2h2aa2	Wfdc3	Ambra1	Psmd14	Rbm41	Pik3ap1	Bach1
Atp6v1c2	Slc23a3	P2rx5	Tcte3	Meis2	Cltc	Pcbd1	Dcaf5	Fkbpl
Lca5l	Wdr47	Gdap1l1	9430097D07Rik	Phlpp1	Tcf7l2	Hdac4	Nrf1	Ralgapb
Krt36	Spnb1	Cldn27	Cox6a2	Phactr2	Lrrc47	Nudc	Cbfb	Bag6
Pnck	Tchh	4932415D10Rik	Dmgdh	Ccdc6	Odf2	Hspb11	Ripk2	Tmem192
Rasgrp3	Tacstd2	Slc28a2	Slco1b2	Scx	Pxn	Arih2	Zc3h15	Rft1
Abhd2	Samd5	Coro6	5430405G05Rik	Yy2	Slc35f5	Ap1g1	Fubp3	BC023829
Susd2	Aim1l	Kcmf1	Fam154b	Zfp664	Gpatch4	Xab2	Pygb	Ptger4
Macf1	Dtnb	Mfrp	Itgb2	Nol10	Arf3	Ran	Prpf38b	Arglu1
Pear1	Pik3cd	Gm6189	Gm14419	Rwdd1	Rab5a	Eps15l1	Erp29	Vps53
Sprr2a3	Cdkl5	Cnga1	Sox15	Zfp82	Ccnd3	AU022252	Gnas	Toe1
A330021E22Rik	Myo15b	Gm14124	Asz1	Gm7694	Zfp142	Rsph3a	Nedd1	Grinl1a
Dusp3	Rasl10b	Pcbp3	Tlr9	Fam3c	Tspo	Yod1	Inpp5e	Mrpl53
E130012A19Rik	Hspa12b	Trim43a	4930429B21Rik	Zfp69	Gpd1l	Rnf214	Myo1g	Ahsa1
Gjb3	Chrm4	Srsf12	C1s	Cyth3	2010106G01Rik	Hnrnpa0	Prr22	Tada2b
Me3	Gm4793	Gpc3	Hist1h3d	Zcchc9	Mthfd2l	Gatad2b	C1rl	Bcdin3d
Fscn1	Proca1	Elavl3	1700123I01Rik	Myo7b	Mtpn	Sec16a	Slc41a3	Tfpt
Vangl1	Cidea	Ccdc74a	Caps2	Tmem39a	Mapk6	Cdc16	Postn	Med29
Krt18	Tmem30c	Hgd	Fam177a	Tnfaip2	Agap3	Dpm2	Gpr137b	Pde12
Cxx1b	Hoxb9	Irx3	Ush2a	Zdhhc5	4933421E11Rik	Clta	Svop	Anks1
Il11	Slc6a13	Csf2rb	Cpne5	Lrp10	Eif2b5	Hspa9	Gas2l1	Brap
Gm17622	Cabp4	Lrrn4	Phox2a	Ccdc55	Rps6kc1	Iars2	Hsbp1	Prrt2
Prkcdbp	Wdr52	Mesp1	Ly6g6d	Itsn2	Kifap3	Hectd1	Fem1c	Kcnk4
Hbegf	Lipc	Prr7	Ccr10	Hipk3	Tmem123	Card14	Efnb1	Znhit1
Herc6	1700022P22Rik	Cecr2	Klra5	Bik	Dhx37	Pex26	Slc35d1	Xrcc5
Ppl	Aox3l1	Cmtm3	Pcdhb17	Gm17231	Pdap1	9030625A04Rik	Sec61a1	Zfp747
Trim30a	Rab44	Hist1h2ad	Lrrc3	Daam1	Wasf1	Ezh2	Cops4	Sympk
Tmem40	Cyp4a32	Robo3	Tdo2	Fem1b	Rhoa	Gcc1	Senp3	Traf2
Myo15	Xrra1	Mpdz	9430070O13Rik	Srfbp1	Exosc10	Cdyl	D6Wsu116e	Map3k12
Gja4	Lbx2	Pkd2l1	Il4	8030462N17Rik	Drap1	Ipo11	Gabarapl2	Psma5
Lims2	Gm6724	Prdm2	Htra4	Cirh1a	Edc4	Zfand5	Stk11ip	Rpl23a
Ntf5	Ttll8	Il1rn	Egr2	Gm10964	Tbpl1	Zfp800	Gtf3a	Trim36
S100a4	Phkg1	Arhgap17	Map6d1	Slc38a1	Slc39a1	Aldoart1	Gm4799	Kif13a
Dsg3	Gm8225	Slc7a1	Abhd5	Zfp828	Rac1	B4galt6	Gm14403	Lrpap1
Dennd2c	St3gal5	Ripk3	Numbl	Rpia	Gm14399	Gng12	Ralbp1	Ubxn7
Apobr	Lst1	Tctn1	Lect1	Fbxw11	2700078E11Rik	Tmem199	Eps15	Dr1
Ccdc102a	Gm12185	Tmem131	Dnahc1	D5Ertd579e	Wrap53	Rnf141	Vmn2r3	ORF19
Syn1	Cd226	Sgk3	Polb	Mfap3	Setd8	Psip1	Trabd	Zfp628

Lpar3	F2rl3	Prr13	Gm14296	Vps45	Usp8	Tspyl1	Tcfcp2	Plp2
Hoxa1	Cldn6	Atf5	Cd37	Hmgxb3	Fermt3	Fgfbp3	Mark3	Cth
Acox2	Wfdc3	Actg1	Art2b	Capza2	Gm14412	Jmjd4	Rsph3b	Sik1
Tspan6	Srms	Eps8	Gm10973	Pkp2	Phc3	Coro1b	Zbed4	Slmap
Dusp10	Clca5	Tmem144	Adam21	Klhl28	Nup153	L1cam	Eed	Gps2
Wtip	Ptgis	Pcgf5	Ube2nl	Erbb2ip	Uhmk1	Ddx59	Fntb	Wdr12
Slc44a2	Abhd16b	Smg7	Kcna3	Zfp462	Terf1	Ubxn1	Kif13b	Nup50
Sez6l2	Wdr63	Gnai2	Slc38a8	Tbxas1	Supt6h	Topors	Atp6v0b	Prpf31
Shroom4	St6gal2	Ctbs	Gm3468	Cfdp1	Zfp52	Gm7935	Cpsf3	Prpf38a
Hmox1	Prss35	Hpcal1	Hsd17b14	Cdsn	Mtrr	Uso1	Iqgap3	Txnl4b
Wwc1	Gm2897	Ywhag	Lhx3	H2-T22	Psmc5	Slc25a27	Rg9mtd1	Il4ra
Ldhd	Il6	Stxbp6	Nefl	Map3k9	Slfn4	Rps6kb2	Tubb5	Ccdc137
Litaf	Gm14436	Calu	Gimap7	Prkar1a	Stac3	Cdk13	Sap30bp	Smarca5
Efna5	Syngr3	Cdkl3	C77370	9130008F23Rik	Gm10943	Gtf3c1	Ing5	Gtse1
Rlbp1	Angptl6	Sat1	Tssk1	Gm6736	Tbc1d23	Hccs	Rsrc1	Smg6
Stx19	4930548H24Rik	Mpp1	Rmnd5b	Gkap1	Gspt1	Xpo5	Gm12355	Bud13
Fgf20	Pga5	Exoc6b	Taf4b	Sccpdh	2310047M10Rik	Zyx	2010321M09Rik	Eif2c3
Gbp10	D830014E11Rik	Trim47	Ptpn11	Rrp8	Rnf40	Serp1	Cryzl1	Trim7
Hist1h3c	Gm14862	Sik2	Slc25a25	Coro2a	Hn1l	Bag5	Tnfaip8l2	Pogz
Sprr2e	Shh	Tbl1x	2010002N04Rik	Sergef	Rpl28	Tmprss2	Gsk3b	Slc39a7
Stab2	6030408B16Rik	Card10	Cdhr2	Kcnj11	Mgea5	Hsf4	Mtmr14	Cldn23
Ccdc89	Btg3	C130074G19Rik	Osbpl9	Abi1	Usp30	Entpd3	Spats2	Paics
Pip4k2a	Esr2	Herc3	Dock7	Spint1	Pip4k2c	Gtf2e2	Ofd1	Nrbp1
Adamts15	Dub2a	Srp54b	Pycr2	Usp36	Kctd5	Nfkbil1	Tmpo	Poldip3
Arhgap6	Sprr2i	Ankrd44	Hvcn1	Cpeb3	Nfkbiz	4921517L17Rik	Atp6v1e1	Gtf2h4
Trpm6	Gm4955	Lrba	Bahd1	Rad23b	Fbxo4	Ccnk	Esrp1	Mfsd11
Cmpk2	Pydc4	Mlf2	Oxct1	Thoc5	Cacna2d1	Zkscan6	Slco3a1	Ssh3
1110012J17Rik	C1qtnf4	Mtap9	Spag9	Cd63	Zc3hc1	Esf1	Spata5	Dph5
Mboat1	Dusp27	B4galt1	Tbc1d1	Eif3a	Gm17253	Ptpn3	Zbtb7b	Mob1a
Pdgfc	Tgfbr3	Gpr151	Gpr44	Pafah1b1	Dstn	Scfd1	Mff	Prkaca
Lmo7	6330512M04Rik	Clic1	C330018D20Rik	Ptp4a1	Psmb7	Setd2	Slc6a6	Rusc1
Bicd1	Otop2	4833442J19Rik	Tle4	Lcmt2	Ripk4	Pard3	Map3k1	Slc1a4
Sgk2	4930595D18Rik	Fzd7	Gm5551	Nfkbib	Fbxw9	Arnt	Cpne2	Rptor
St6gal1	Gm8991	1110067D22Rik	Acta1	Selenbp2	Mrrf	Med18	Acer3	Ap2s1
Tsc22d2	Myh7	Ddx47	Nudcd3	Dync1h1	Klhl26	Exosc1	Ttc1	Cebpz
Mcam	Ninl	Ric8	Skil	Clca1	Apln	Ppp2r5a	Zfp518b	Myo10
Ptpn21	Metrnl	Rps19-ps4	Trim26	Zfyve20	Carm1	Zfp14	Actr8	Ythdc1
Sh3tc1	Rnf19a	Bbs5	Ipo13	Psmc2	Tmem159	Cand1	Trim68	Exoc8
Ldoc1l	Kif3a	Snx16	Gnl1	Lpar6	Ddx27	Anp32b	Lrrc31	Caprin1
Tm4sf4	Nfil3	Fam161b	Fam110c	Dcaf12	Zc3h14	Zfp605	Hfe	BC017647
Steap1	Rhbdf1	Ccdc78	Shroom3	Rasal2	Nfyb	Atf1	Fam178a	Fam160a2
Otud1	Csf1	Phldb3	Zfp251	Ipo11	Dis3l	Per1	Tubgcp5	Mon2
Irgm2	Slc44a4	Sfn	Pgcp	Uba6	Sin3b	Bbs1	2610028H24Rik	Arhgap5
Car2	1600029D21Rik	Ppic	Gm11084	Orc2	a	Hapln4	Gtf2f2	Atg7
Ifi27l1	Fam82a1	Ect2	Trim8	Zc3h12b	Smpd1	Zfp395	AF085738	Vps4b
Nbl1	2610018G03Rik	Ccdc104	A730068I03Rik	Map3k5	BC037034	Rpgrip1l	Prkab1	1600016N20Rik
Gm13695	Zfp296	Ccng2	Mier3	Wac	BC037034	C130022K22Rik	Gm7964	Hnf4a
Efhd1	Sybu	Tor1aip2	Gm10382	Haus5	Arfgef1	Upf2	Gm16378	Atf2
Ccdc120	Efhd2	Adar	A330041J22Rik	Pggt1b	Pdzd7	Dcun1d5	Clasp1	Magohb
Tln2	P2ry2	Mpv17l	Med10	Wdr5	0610037P05Rik	Rala	Slc15a4	5430437P03Rik
Sprr2g	Timp3	Arhgdig	Serinc1	Shcbp1	Dennd4a	Fbxo38	Hnrnpu	Taok2
Car6	Edn3	BC030499	Dock9	Arhgef40	Eif2ak4	Nmt1	Cpeb4	Usp42
Gm5129	Lrrc27	Acap3	Gnmt	D8Ertd82e	Sbf1	Zswim1	Klhl20	Pcm1
Myh11	Man2b2	Lca5	Dact2	Kras	Bax	Mxra8	Trim23	Bcl2l2
Gm13283	Tmem2	Gm10277	Pnp2	Cry1	Prss8	Fam120a	2900006K08Rik	Tmem167b
Gm17664	Gm16066	Gm340	Prss48	Acot7	Prpf3	Tmub1	BC030336	Hook2
2310014H01Rik	Tada3	Eif6	Kif1c	Psmd12	Dctn1	Zc3hav1l	Dis3	Amigo3
Palld	Paqr8	Slc5a10	Kpna4	Got1	Rps19bp1	Syngr2	Uspl1	Nos3
Zmynd15	4930539E08Rik	Zdhhc18	Klhl11	Npcd	Camsap3	Rae1	mt-Nd2	Prune2
Gas1	Dlgap1	Cdkl2	Snx8	Chpf	Rrp1b	Fnbp1	Dbnl	Gpank1
Cep170	Ptpn12	Asns	Nr6a1	Atp6v1h	Nsun6	Ebag9	Rgl1	Fat1
Flnb	Efhc1	Grhl1	Tdrd12	Rab3gap2	Map4k4	Ddx1	Taf15	Nacc1
Isg20	Tpm4	Wdr26	Cerk	Trim24	Taf3	Strap	Tcf25	Iqcc
Fam198b	Itgav	Mapk12	Strn	2310044G17Rik	Itga10	Kctd6	Mfap1b	Ecd
Pmaip1	Nipal1	Adora2a	Mtmr9	Cdc42se1	Fbrs	Klhdc4	Polr2g	Brix1
Tjp1	3110062M04Rik	Fam89a	Rassf3	Rgma	Bag3	Ipo5	Pelp1	Ap1s1
Pkhd1	Gldc	Tbc1d10b	C630004H02Rik	Haus6	Krit1	Diap1	4933407C03Rik	Med11
Pik3cb	Gch1	Serpinb1c	Sh3glb1	Bet1l	Zfp516	Otud4	Agpat6	Trip13
Smad9	Fam46d	Il1rap	Slc16a2	Ppp1r11	Kdm1b	Nap1l1	Lmbr1	Dnajc1
Lrrfip1	Cryz	Plekhn1	Ppp2r5b	Zbtb42	St13	Sf1	2810004N23Rik	Trp53i13
Ttn	Trib1	Smad6	Fhl2	Spopl	Ap1m1	Ap3b2	Pof1b	Wrb
Gm7665	Mamld1	Hivep2	Sbds	Cpne1	Serbp1	Eif5b	Cox16	Mzt1
Arhgap44	Arl8a	Polr3d	Chst12	4930471M23Rik	Tmem106b	Gimap4	1810031K17Rik	Yme1l1
Tcte2	AW555464	Krt83	Prkar1b	Kcnj13	H47	Ralgds	Lrp12	Mettl16
Ccl20	Gm13691	Slc38a7	Nmd3	Serpinb6a	Nckap1	Spg21	Gdi1	Pitpnb
Osbpl5	Gm11102	Rnf148	Tnfrsf23	Magi1	Mphosph10	Apc	Snrpa1	Asap3
Fos	Jun	Fam164a	Cbara1	Cald1	Fam116a	Exosc3	Zfp526	Ltc4s
Pdgfb	Tmem184c	Vopp1	Wdr1	Zfp513	Igf2r	1500002O20Rik	Rab10	Aebp2
Smad7	Capsl	Nt5c2	Hk1	Urb1	Lrch4	Btbd9	Dclre1b	Baiap2l1
1700047I17Rik2	Itpripl2	Ercc1	Tmem128	Ywhae	Calhm2	Gnai3	Rnps1	Chm
Rbms1	Trim16	Gm10643	Zc3hav1	Spry2	Cdca4	Usp39	Lrp8	1110038F14Rik
Acot9	Sik3	Tjap1	Dnajb7	Zfr	Psmc1	Gm447	Ruvbl2	Tcf12
Kcnq4	Atg16l2	Gm7353	Ddr1	Ctnnd1	Recql5	Ttbk2	Gm14420	Sec61g
Mdga1	Slc9a2	Gm10974	Iars	Tmem168	Siah2	Snapc2	Tpp2	Kctd1
Tnfrsf9	Gm9887	Zswim6	Mapk7	C130039O16Rik	Utp18	Stip1	Polr1a	1700021C14Rik
Gm17391	Eif4ebp1	Tspan15	Atf7	Tcf20	Rnf2	Atp6v1a	Ggnbp1	Snrpb
Gm14440	Flrt3	Pde3b	Faf1	Ehbp1	1110004E09Rik	Dem1	Abce1	Ict1
Tgfb3	Lrrc46	Zc3h3	Gatad2a	Capns1	Smg1	C1qbp	Synrg	Snap23
Gm13694	Mkrn3	1700030J22Rik	Lrrc66	Krt76	Snrnp200	Bri3	Fam83b	Mrpl48
Dmrt2	Adm	Ldlrap1	Tbc1d10a	Spn	Ep300	Cul5	Fitm2	Ythdf2
Zmynd12	Eif2s2	Akirin2	Epo	Tyrobp	Zbtb34	Wdr74	Mrps10	Taf12

Gm10696	Slc45a3	Rab11fip2	Aprt	Xlr3a	Pja2	Rnf126	Nrd1	1110002L01Rik
Eda	Kdm3b	Eif3c	Dlgap4	Tesc	Sri	Brms1l	Rcan1	Plrg1
Ccdc40	Pea15a	Igsf3	Itch	Zcchc12	Lrrc39	Zrsr2	Tmem184a	Ciao1
Tnni1	S100a10	Cttn	Ahi1	Col27a1	Fxr2	Rfx1	Cdc6	Dnalc1
Gabrr2	Trio	Btc	Nphp1	X99384	Rnls	Rpusd4	Fam115a	Dhx8
Kcnv2	Bcor	Gtpbp2	Eif1	Rce1	Stx3	Ccdc122	Cited2	Jmjd6
A230065N10Rik	Krt84	Sacs	Polr2c	Gtpbp5	Mtbp	Nup188	Mad2l2	Snx25
Gm10129	Igfbp3	Flii	Nln	Osbpl8	B3gat3	Map2k1	Wdr41	Mll2
Capn5	Bicd2	Uba7	Brms1	Nars	Pef1	Hr	Cep55	Stim1
Gm7008	Vdac2	Slc39a6	Cst3	Psmd1	Crlf2	9830001H06Rik	Zfp867	Unk
Tulp2	Abca5	Cnn3	Gm6900	Zfp791	D6Wsu163e	1110004F10Rik	G6pdx	Smu1
Rab3b	Rp2h	Fam13b	2310009B15Rik	Smndc1	Rapgefl1	Numa1	Atg4c	Etv3
Slco2a1	Hist1h1e	Myo1d	Rbm19	Pmpca	Srf	Rad17	Gm10644	Trappc8
Steap2	Ift57	2410089E03Rik	Casp4	Sirt2	Bex4	Eif3j	Fbln2	Abhd16a
Phlda1	Impdh1	Stat3	Npepl1	Fgd4	Elovl1	Gna12	Itgae	Dctn3
Crlf3	Slc7a5	Sh2d4a	Fkbp11	Ccdc76	Slc35c1	Gal3st4	Pfkp	Gata5
Agtpbp1	Rras	Prkce	Crb3	Synj2bp	Lins	Armcx3	9130221H12Rik	Gm6169
Prrg4	Kctd9	Gm17239	Skap1	Zfx	Psma4	Sap30	Sub1	Gtf2h5
Flot2	Pkp3	Hspa8	F8	Atp6v1g1	Erf	Slc4a1ap	Tuba1a	Gbas
Artn	Gm7854	Adamtsl5	Thap8	Slc2a12	Wbp7	Sec23ip	Twf2	Ralgapa1
Emp2	Actn4	Gpr19	Fip1l1	Eif2ak3	Arpc1b	Patl1	Twistnb	Tacc3
Grasp	Dtx1	Eif2b3	Tcfe3	9630033F20Rik	Eif3g	Atp6v1c1	Bbs7	Tpt1
Clcf1	Gnb5	Gm5884	Mthfd1l	Pls3	Necap2	Snx27	Optn	Mtus1
S100a11	Aqp5	Diap2	2010003K11Rik	Zfp61	Tmed9	Chmp2a	Zfp821	Rpl32-ps
Vgll3	Chst3	Ctsf	Mybph	Dok3	Cdhr5	Psmd5	Rnd2	Usp1
Rapgef2	Itgb4	Tnnc1	Dync1li1	Gm16372	Ulk2	Ctdp1	Map3k11	Tulp4
Prokr1	Igf1r	Tnnc1	Smcr7	Rap2a	Actr2	Crcp	Gm14698	Ube3c
Rasgef1b	Erc2	0610009L18Rik	Spag17	Lrrc41	Ssrp1	Lonrf1	Ctnnbl1	Eda2r
Nlrp10	Ikzf4	Rdx	Pdlim5	Dnm1l	Snx33	Lsr	Alkbh6	Zbtb44
Slc7a11	4930500M09Rik	Agxt	Cpeb2	Gnal	Cdc27	2900060B14Rik	Elf2	Ube2v2
6030419C18Rik	Sdc2	Usp53	Neurl1a	Traf5	6430527G18Rik	Ass1	Huwe1	Eif2b4
Cxcl16	Ddah2	Unc5b	Stag2	Kif5b	Tsg101	Serpind1	Polr3b	Eif5a
Tead1	Mllt4	Xpr1	Mbd2	Psmd3	Tagln	Dnaja2	Srek1	Nsun3
Adamts1	Hs3st1	S100a13	Akap17b	Arhgef1	Dnm2	Rab13	Nol6	Rgs12
Ahrr	Blnk	Sh3d19	Cldn12	Ighmbp2	Kdm4a	Sssca1	Sltm	Sclt1
2210011C24Rik	Camk2a	Csf1r	Pttg1ip	9330160F10Rik	Zfp446	1700012B15Rik	Xpo1	Baz2a
Naalad2	Fam176b	Slk	Med27	Pom121	Ankle2	Slu7	Pdha1	Mtdh
Zmynd10	Anxa6	Ncoa7	Zfp94	1700106N22Rik	Sec62	Actr1a	Gm5506	Rab18
Hist1h2bg	Ap1s3	Zfp295	Rheb	Myom3	Mid2	Cnot2	Arntl2	Agtrap
Porcn	Psd3	Spnb2	Zfp809	Orai1	Relb	Rab1	Trappc3	Tsc22d4
Samd9l	Cd276	Klhl22	Pkd1	Armc6	Ppp2cb	Gcnt7	Pex14	Hist1h1c
Ngfrap1	Hsh2d	Wsb1	Slc39a4	Pofut2	1700109H08Rik	Ndfip2	Raly	U2af2
9530077C05Rik	Tnk2	BC021614	Klhl18	Ppie	Utp3	Crebbp	Rab14	Mif4gd
1700069B07Rik	Zfp385a	Itga3	Esyt3	Qrich1	Akirin1	Pibf1	Fbxo42	Rfwd2
Car5b	Ltbp3	4930578N16Rik	Wdr96	Gm10567	Ankrd52	Kat5	2610001J05Rik	Plk4
Ubtd2	Jrk	Cdcp1	Ng23	C1galt1c1	Prhoxnb	Nelf	Psma3	Chmp6
Pwwp2b	Flrt3	39697	Mab21l1	Calm2	Chd1	9430016H08Rik	Bin3	Klhl5
Rin1	BC125332	Gm2058	Tom1l1	Rb1	Gemin7	Ddx56	Ddx54	Gprasp1
Gm3625	Parva	Tbc1d25	Ada	2310046K01Rik	Nupl2	2310030N02Rik	Rpl10a	Fam89b
Wnt16	Cd2ap	Trim25	Gm17409	Usp14	Gabarapl1	Cnnm4	Arfrp1	39513
Tmem150c	Gm17438	Tpm3-rs7	Elovl2	Mex3d	Cnot4	Tbc1d2b	Lyn	Cdh23
B230317F23Rik	Ncoa3	Cdkn1a	4930597O21Rik	Rrp1	Eif4g2	Ctbp2	Psmb2	Gbp8
Eno4	Derl3	Slc39a10	Ovol2	Dhx29	Ccdc92	Nfix	Rabl3	Sirt5
Gm13696	Chka	Habp4	Znfx1	Slc35b3	Nip7	Herpud1	Camk2d	Gm10130
BC022687	Luzp1	Lims1	Egfl7	Plekha7	Zfp259	Gm9833	Tmem41a	Gm5356
Nceh1	Ppfibp1	Perp	Ube2r2	Alkbh2	Rad23a	Klf5	Pitrm1	Tnni3
Socs1	Taf9b	Zfp593	C2cd2	Zfp511	Arl6	Lrig2	Srebf2	4930523C07Rik
Oas1a	Tmem171	Plekhm2	Plxnb2	Anapc2	Lrrc68	Rc3h2	Ccdc99	Krt72-ps
Aqp7	Slc2a4	D17H6S56E-3	Csnk2a2	Tor1aip2	Nagk	Cyp4f39	BC005561	Itgad
Tspan4	Nacad	Abcg4	Ipo4	Zfp143	Adal	Hivep3	E2f3	Krt74
Maff	Gm14327	Sepn1	Arl5b	Nup210l	Stx18	4933413G19Rik	Ppp1r8	Cd3e
Gpr124	Lgals7	Dhh	Gss	Fam195b	Kars	Pla2g6	Parp11	Slc12a5
9030425E11Rik	Styx	Bhlhb9	Agpat2	Map2k4	Pramef8	Pip5k1c	Sytl4	Wnt9a
Plec	Sdc3	Fgf15	Psmd2	Gltp	Cdk9	Rbm22	Tnks	Ankrd13d
Frmd4b	Sh3bp4	Rell1	Fkbp1a	Lad1	Adipor1	Pno1	Snrnp70	1110006G14Rik
Tpm2	Anxa7	Aff4	Dnase1l2	Zrsr1	Ppp2r1a	Gba2	Cobra1	Krt73
Tmem231	Mtap7d1	Hras1	Ptk2	Dmtf1	Kpnb1	Mbip	Cdk16	Krt2
Serp2	Tnfaip1	Cpox	Tead3	Myst4	Fam45a	Med8	4932443L11Rik	A930033H14Rik
Sod3	D15Ertd621e	Krt15	Mthfsd	Leo1	Napa	Snd1	Zmat1	Serping1
Rhod	Inf2	Nrp1	4933433P14Rik	Tsga10	Dpcd	Fam96b	Gm17067	Gm15409
Ncrna00085	Tmsb10	Obfc1	Snhg11	Cad	Lin54	Tpbg	Tmem121	Ano3
Snai3	Lrfn4	Plce1	Zbtb37	Arl2bp	Smn1	Mcart1	9030418K01Rik	Amh
Arnt2	Rnf133	Piwil2	Git2	Cct7	Hivep1	Cul1	Zfp455	Krt5
Gm7461	Bcar1	Myom1	Atp6v0a1	B430203G13Rik	Gpr107	Ncoa4	Ccdc19	Grik4
39691	Ptpn23	Rhov	Npr1	Faim	Ints6	Prrc1	Ppp1r15b	Lrrc50
Gem	Mab21l3	Rg9mtd2	Snrnp27	Oxr1	Taf10	Prmt6	Prc1	Tcf7l1
Spire1	Srgap1	Il6st	Ktn1	Tmem150a	Aff1	Ing3	1810074P20Rik	Il16
Lama3	Smurf1	Rps6kl1	Taok1	Brd4	Ccdc116	Nsun2	Pa2g4	A330017A19Rik
Olfr56	Jph1	Cebpb	Fbl	Uba5	Rpp30	Lypla2	Htt	Gbp4
Dusp18	Actn3	Csnk1e	Gm16380	Ahctf1	Cep120	Cenpj	Pus7l	Nup214
Ddah1	Pcdha11	Hax1	Manf	Mafg	Sema4d	Wdr3	Ift74	Naca
2010002M12Rik	Htra1	Wdfy2	Acbd6	Eea1	Fpgs	Syf2	Khdrbs1	Commd7
S100a6	Rab23	Krt20	Bid	Ttll12	Ttll3	Rbm8a	Vac14	Smagp
Fam123a	Cxcr7	Sv2a	Stk39	Mtmr7	D4Ertd22e	Ifnk	Slc10a3	D1Bwg0212e
Reep2	Slc9a4	Trpv3	Nudt4	Commd2	Eif4g1	Mll3	2610002I17Rik	Lypd1
Dnahc7b	Nsf	Agfg1	Gm11031	Rabl2	Vps4a	Pnkp	Cyb5r4	Amd1
Kcnu1	Tnrc18	Eaf1	Ccdc148	Phf3	Vcp	Esd	Lnx2	Nudt14
Tnfrsf22	Als2cl	Ostf1	Bbc3	Tmc3	Odc1	Ift88	Pptc7	Pbrm1
1110002E22Rik	Eif4e3	Sh3kbp1	Gm5434	Zc3h7a	Taf1c	Ik	Ppil3	Zfp27
Neu1	Prmt2	Fam116b	Scyl1	Fgd6	Ube2j2	Spty2d1	Shkbp1	Atp6ap1
Epha4	Mesdc1	Itprip	Rab8b	Trafd1	Rnf20	Osbp	Apobec1	Ubqln2

5330426P16Rik	Tubb2b	Vezt	2810453I06Rik	Spin1	Rabggta	Fnbp1l	Wibg	Narfl
Eid2	Fggy	Fes	Dlg1	B230120H23Rik	Dync1li2	Pop4	Vdac1	Phf14
Zbtb10	Gm10306	Ap2a2	Phlda3	Arhgap18	Ccdc23	Atic	Rnf44	Ide
Asap1	Lrrtm2	Nop14	Cxx1a	Ube2w	Ddx31	Cdc34	Usp43	Nipal2
Gpx7	Esyt1	Srm	Mmp28	Cdk12	Crk	Grinl1a	Nsd1	Tomm20
Arid3a	Nxnl2	Spryd3	Zfp712	Rpl34	BC019943	Gipc2	Bnip3l	Wdtc1
Grb10	Myh9	Ptprd	Usp6nl	Rnf31	Col7a1	Slain2	Tmem5	6720456B07Rik
Lamc1	Ltk	Chic2	Myo9b	Eif2ak2	Sf3b4	Ruvbl1	Uhrf1bp1	Hnrnpa1
Il28ra	2310016C08Rik	Tspan12	Vwa5a	Dhx34	Ino80e	Ddx10	Wdr70	Btf3
Phlda2	Mvp	Ept1	Slc26a1	Nbeal1	Fam193a	Gfod2	Ubb	Mdn1
Pam	4930562F07Rik	Dlc1	Arpc2	Gm505	Marveld2	Trrap	Eme1	Cstf2t
Fam43a	Ccnd1	Zdhhc19	Ttl	Atxn2l	Vps8	Thap1	Sf3b3	Elf1
Egfr	B4galt2	Drp2	2810453I06Rik	Etf1	Armc7	Rab3ip	Rftn2	Eef1a1
Tmem130	Gm10020	Rnf207	Eif3d	1810029B16Rik	Csrp2	Usp47	Gmeb1	Caskin2
Tlr4	Ddit3	Rpl26-ps4	Gm17307	Gm13139	Eif5	Exoc2	Rnf4	Ihh
Epha3	Ttll7	9430002A10Rik	Pcdh1	Appl1	Ccdc64b	Tlcd2	Cinp	Lactb2
Gm17343	Myo9a	Hes7	Rab22a	Tprn	Scand1	Hdlbp	Mapk14	Cdk5rap1
Kcnk7	8430427H17Rik	Gm15440	Pdcl3	Smad2	Aktip	Prmt7	Ppm1d	Cdkn2b
Gm106	Sh3pxd2b	Fam57b	Gm10563	Las1l	Samd8	Gtf3c3	Poc5	Rps12-ps3
Rnf17	Hoxb6	Trpv2	Parp3	Wipf2	Met	Axin1	Copb1	Med21
Gm17379	Tle6	Gm9949	Ubxn4	Arl8b	Adat3	Noll1	1700120B22Rik	Hipk1
D830030K20Rik	Zfand2a	Rsad1	Tmem65	1700088E04Rik	Stk24	Prdm4	Ptp4a3	Zcchc17
Espnl	Csgalnact2	Otud3	Rpusd1	Gm962	Ap3b1	Nufip1	Tdg	2700094K13Rik
Gm12824	Egf	Afap1l1	Adprh	2400003C14Rik	Tpr	Tnpo1	Pam16	Dhx57
Lrrn4cl	Xk	F8a	Smap1	39511	Ctdsp1	Map3k6	Sufu	Wdr8
Slc22a27	Igsf9	Ypel5	Snupn	Ccm2	Lsm14a	Ftsjd2	Acap2	Nosip
Efna5	Stc2	Lrrc51	Camsap1	Hars	1700022I11Rik	Naa20	Caml	Ado
Slc22a28	Emp3	Pawr	Gm6710	Ccdc90a	Mpi	Sdr42e1	Gpatch3	Polr1b
Islr2	Dmwd	Inpp4b	Dnali1	Jak3	Ids	Uvrag	Usp20	Tmem216
Chrna1	Plekhh3	Prnp	Kcnip2	Spdya	Lrrc20	Rapgef1	Tmem138	Caprin2
Gm14305	Gm3252	Tmem38b	Pdk4	Elovl5	Hebp1	Satb2	Zfp319	Ift172
Ccrn4l	39698	Acot2	Kcna7	Pole	Bmyc	Spred2	Chuk	C330011M18Rik
Arf2	Pygm	Src	2310014L17Rik	Acsl4	Lrrc8a	BC017643	Zbtb2	Slc12a6
Rnf213	Gm6788	Fam83g	Gak	Plaa	Smarcb1	Riok3	Ccdc25	Myst2
Sprr1a	Actn1	Fzd6	Tspyl2	Tuba8	Gipc1	Sugt1	Pppde2	1200014J11Rik
Rai14	Fbxo6	C77080	Hcfc1r1	Calr3	Afg3l1	Ftsj3	Cops5	Rpl6
Rhof	Tpm1	Elf4	Exoc5	Dact1	Mapt	Med4	Ift80	Golga5
Hsd3b3	Ly6e	Dnajb2	Map1lc3a	Tnfrsf4	Glt25d1	Mad1l1	Crtc2	Birc6
Icam1	Ier5	Gm10638	Papd7	Krt79	Akt1	Mettl6	Slc46a1	Wdr20a
Prkcc	Rai1	4932425I24Rik	Dnttip1	Ikzf1	Ranbp10	Fbxo34	Rars	Ercc4
Irf7	Mdfi	Kcnc1	Ap2b1	2410127L17Rik	Arhgap12	Zfp708	Zc3h13	H2-Q7
Rtn4r	Gm14137	Tdrkh	Casp8	BC061194	Thap7	Btbd19	Sgpp1	4930455C21Rik
Arhgef10	Mtap1s	3110043O21Rik	Mta1	2310001K24Rik	Xpnpep3	Stau1	Zfp429	Metap1
Ccdc85b	AI747448	Gm9774	Pabpc4	A530084C06Rik	Arhgef7	Ccnt1	Nrip2	Ccne1
Fcho1	Hist2h4	Tap1	Rltpr	Mfn2	Ddb1	Polr3c	Zfp28	Fbxo36
Hrh1	Nepn	Klf3	Aars	Dus4l	Gtf2a1	Prrc2c	Ehmt2	Fbrsl1
Morn4	Gml	Gm17674	Dhrs13	Gm6483	Papd5	Drg1	Sf3b2	Eml5
Elk3	Gm128	Kremen1	Nucb2	Wdr34	Heatr7a	Ints5	Mrto4	Mcoln1
Myadm	C1qtnf5	Lrrc1	Inpp5a	Crtap	Cdc42	Naip1	Txnl4a	Letm1
Arc	Zfp872	Cenpt	Fdx1l	Cd3eap	Sap130	Gnl3l	Pmm1	Rbm25
Otud7b	I830134H01Rik	Cdk14	Trip10	Gyk	Med30	Trim12a	Orc3	Klhl21
Fam59a	Hist1h1b	Wbp5	Fmr1	Sorbs1	Abcf1	Faf2	Asl	Mta2
Ifrd1	Cldn8	Parm1	Rod1	Zfp112	Moap1	Setd1b	Mettl10	Pdpk1
Tagln2	Hic1	2200002D01Rik	Slc2a1	Arf4	Prrc2a	Ppp1r7	Mcm3ap	Ube2a

Revival Stem Cell signature

Roulis, et al. Nature 580.7804 (2020): 524-529.
Ayyaz, et al. Nature 569.7754 (2019): 121-125.

S100a6	Rpl41	Lmna
Clu	Rpl27	Ranbp1
Ly6a	Nme1	Rplp0
S100a11	Ran	Rpl29
Ly6d	Gm10073	Sat1
Sprr2a3	Rpl35	Eif2s2
Mif	Dynll1	Wdr89
Krt19	Sprr1a	Hsp90ab1
Krt7	Rps2	
Npm1	Rps12	
Krt18	Anxa1	
Rps27l	Rps12-ps3	
Fxyd3	Erh	
Xcl1	Gm10076	
Anxa2	Rplp1	
Ccnd1	Pglyrp1	
Ncl	Rps28	
Ccnd2	Nhp2	
Cd44	Snrpg	
Ptma	Rps26	
Anxa3	Gm8730	

EIF_PATHWAY

https://www.gsea-msigdb.org/gsea/msigdb/human/geneset/BIOCARTA_EIF_PATHWAY.html

EEF2
EEF2K
EIF1
EIF1AX
EIF2S1
EIF2S2
EIF2S3
EIF3A
EIF4A1
EIF4A2
EIF4E
EIF4G1
EIF4G2
EIF4G3
EIF5
EIF6

Table S2: Metabolite abundance between samples

id	MS2 name	WT1_si	WT2_si	WT3_si	WT4_si	WT5_si
1	1-Methylhistamine	183.4862385	235.8366271	259.9557522	123.3632863	183.2884097
2	2-Aminoadipate	1628.440367	1870.88274	581.8584071	1643.132221	517.5202156
3	2-Aminoisobutyric acid	13990.82569	24374.17655	9435.840708	18356.86778	22371.96765
4	2'-Deoxyadenosine	902.5229358	619.2358366	649.3362832	1211.810013	439.3530997
5	2'-Deoxycytidine	391.0550459	625.8234519	295.3539823	582.7984596	144.2048518
6	2'-Deoxyuridine	1422.018349	1488.801054	1559.734513	1202.824134	2506.738544
7	2-Hydroxy-2-methylbutyric acid	275.2293578	358.3662714	330.7522124	335.0449294	431.2668464
8	3-Hydroxyanthranilic acid	364678.8991	478260.8696	326327.4336	446726.5725	423180.593
9	3-Nitrotyrosine	581.4220183	625.8234519	513.2743363	531.4505777	402.9649596
10	3-Phosphoglycerate	85.66513761	84.32147563	26.54867257	54.81386393	57.54716981
11	4-Hydroxy-3-methoxyphenylglyc	6.112385321	14.09749671	23.67256637	6.842105263	14.42048518
12	4-Hydroxybenzoate	4139.908257	2687.747036	2024.336283	3363.286264	2021.563342
13	4-Pyridoxic acid	1364.678899	4927.536232	962.3893805	4557.124519	1630.727763
14	5-Hydroxyindole-3-acetate	170.8715596	386.0342556	23.5619469	465.9820282	187.3315364
15	5-Hydroxytryptophan	131.8807339	108.9591568	44.24778761	143.7740693	157.6819407
16	5-Methyltetrahydrofolic acid	48.96788991	105.4018445	11.83628319	102.6957638	71.96765499
17	Acetoacetate	584.8623853	952.56917	141.5929204	1082.156611	2142.857143
18	Acetylcholine	620412.844	715415.0198	620575.2212	666238.7677	617250.6739
19	Acetyl-CoA	134.1743119	189.7233202	100.3318584	287.5481386	100.6738544
20	Aconitate	1238.53211	1646.903821	1294.247788	1412.066752	1215.633423
21	Adenine	1158.256881	2332.01581	1183.628319	1566.110398	884.097035
22	Adenosine	4690.366972	7760.210804	3373.893805	6842.105263	5835.579515
23	Adenosine	290137.6147	337285.9025	290929.2035	315789.4737	384097.035
24	Adenylosuccinate	220.1834862	2028.985507	217.920354	13.73555841	1347.708895
25	ADMA	26032.11009	55204.21607	27986.72566	69319.64056	21967.65499
26	ADP	2.94800885	1818.181818	1460.176991	2811.296534	1280.32345
27	Alanine	244266.055	470355.7312	257743.3628	436456.9961	382749.3261
28	Allantoin	844.0366972	1159.42029	431.4159292	1296.534018	683.2884097
29	AMP	96100.91743	168642.9513	46792.0354	125160.4621	68059.29919
30	Anthranilate	4071.100917	5362.318841	5409.292035	6919.127086	4043.126685
31	Arginine	176605.5046	393939.3939	125000	323491.656	176549.8652
32	Argininosuccinate	311.9266055	513.8339921	217.920354	595.63543	439.3530997
33	Ascorbate	35435.77982	140.9749671	6946.902655	93581.51476	14150.9434
34	Asparagine	13876.14679	22397.89196	7555.309735	25417.20154	11226.41509
35	Aspartate	39105.50459	62582.34519	22455.75221	67265.72529	35040.43127
36	ATP	24.4266055	14.09749671	23.67256637	13.73555841	7.18328841
37	Betaine	784403.6697	504611.3307	547566.3717	501925.5456	746630.7278
38	Bilirubin	1536.697248	2028.985507	1038.716814	794.6084724	1711.590296
39	Biotin	5378.440367	8458.498024	6006.637168	13222.07959	5822.102426
40	cAMP	605.5045872	998.6824769	377.2123894	1225.93068	633.4231806
41	Carnitine	125000	177865.6126	120575.2212	200256.7394	164420.4852
42	Carnosine	1071.100917	3030.30303	2621.681416	1283.697047	1698.113208
43	CDP	6.112385321	2.94800885	2.94800885	6.842105263	2.94800885
44	cGMP	6.112385321	28.06324111	11.83628319	13.73555841	14.42048518
45	Chenodeoxycholate	128.440367	646.9038208	29.53539823	82.15661104	21.56334232
46	Cholate	3853.211009	35309.61792	2953.539823	3299.101412	2601.078167
47	Citrate	6490.825688	2.94800885	6526.548673	4030.808729	5700.808625
48	Citrulline	14564.22018	19631.09354	11393.80531	16302.9525	9231.80593
49	CMP	4495.412844	8313.570487	4015.486726	14249.03723	6374.663073
50	Cobalamin	520.6422018	421.6073781	1592.920354	116.4313222	597.0350404
51	Cotinine	3807.33945	2239.789196	1294.247788	2605.905006	2452.830189
52	Creatine	608944.9541	698287.22	620575.2212	706033.3761	809973.0458
53	Creatinine	197247.7064	239789.1963	181415.9292	297817.715	283018.8679
54	CTP	6.112385321	2.94800885	2.94800885	6.842105263	7.18328841
55	Cystamine	122.706422	183.1357049	111.7256637	68.54942234	86.25336927
56	Cystathionine	42.88990826	7.022397892	41.26106195	27.34274711	21.56334232
57	Cysteamine	2626.146789	2951.251647	1902.654867	3658.536585	2371.967655

58	Cysteine	287.8440367	555.9947299	265.4867257	328.6264442	115.0943396
59	Cytidine	15481.65138	19762.84585	9623.893805	19127.08601	10363.8814
60	Cytosine	2018.348624	9446.640316	6725.663717	6251.604621	4797.843666
61	DCDP	2155.963303	5744.400527	2444.690265	13992.29782	8140.161725
62	DCMP	55.04587156	225.2964427	59.07079646	424.9037227	115.0943396
63	DCTP	2.94800885	2.94800885	2.94800885	2.94800885	2.94800885
64	D-Erythrose 4-phosphate	238.5321101	577.0750988	560.840708	630.2952503	582.2102426
65	D-Fructose 1,6-bisphosphate	150.2293578	1227.931489	64.93362832	944.801027	1401.617251
66	D-Fructose 2,6-bisphosphate	73.39449541	98.41897233	35.39823009	75.35301669	71.96765499
67	D-Glucuronic acid	2052.752294	4189.72332	1969.026549	4967.907574	2021.563342
68	D-Glycerate 2-phosphate	4506.880734	6350.461133	2754.424779	6315.789474	1725.067385
69	DHAP	113.1880734	927.5362319	59.07079646	458.279846	86.25336927
70	Dihydrofolate	12.2706422	7.022397892	5.896017699	13.73555841	2.94800885
71	Dimethyl glycine	276376.1468	112.516469	141.5929204	51.3478819	71.96765499
72	Dopamine	78669.72477	106719.3676	75553.09735	125160.4621	66037.73585
73	D-Ribulose 5-phosphate	1892.201835	4953.886693	1100.663717	4801.026958	1886.792453
74	DTMP	220.1834862	49.14361001	189.159292	671.3735558	50.26954178
75	DTTP	91.74311927	84.32147563	35.39823009	95.89216945	93.5309973
76	DUMP	4816.513761	5612.648221	2112.831858	4801.026958	1994.609164
77	DUTP	6.112385321	14.09749671	5.896017699	13.73555841	7.18328841
78	Epinephrine	2064.220183	3702.239789	2300.884956	3761.232349	3194.070081
79	Folate	2.94800885	21.08036891	2.94800885	2.94800885	2.94800885
80	Folinate	18.34862385	7.022397892	11.83628319	2.94800885	7.18328841
81	Fructose	152522.9358	256916.996	122787.6106	385109.1142	111859.8383
82	Fructose 1-phosphate	3864.678899	16864.29513	3794.247788	16046.21309	10795.14825
83	Fumarate	1639.908257	4729.907773	2101.769912	5288.831836	1522.911051
84	GDP	1250	2055.335968	584.0707965	1630.29525	640.1617251
85	Geranyl pyrophosphate	642.2018349	695.6521739	271.0176991	924.2618742	222.3719677
86	Glucose 1-phosphate	2580.275229	11093.54414	3407.079646	12336.32863	9380.053908
87	Glutamate	181192.6606	242424.2424	172566.3717	188703.466	181940.7008
88	Glutamine	76146.78899	144927.5362	50331.85841	151476.2516	95956.87332
89	Glutathione	15366.97248	34387.35178	7831.858407	36970.47497	18598.38275
90	Glyceraldehyde	201.8348624	210.8036891	136.0619469	363.2862644	165.7681941
91	Glycerol	67.31651376	147.5625823	47.23451327	184.8523748	28.84097035
92	Glycerol-3-phosphate	36697.24771	76152.83267	32522.12389	121822.8498	41509.43396
93	Glycine	18233.94495	31488.80105	16482.30088	34788.18999	23450.13477
94	Glycochenodeoxycholate	6.112385321	21.08036891	29.53539823	13.73555841	28.70619946
95	Glycocholate	392.2018349	1026.350461	566.3716814	349.1655969	338.2749326
96	GMP	3153.669725	10513.83399	3373.893805	12965.34018	9407.008086
97	GTP	2.94800885	7.022397892	2.94800885	2.94800885	14.42048518
98	Guanine	2809.633028	6113.306983	2743.362832	5545.571245	3894.878706
99	Guanosine	28899.08257	49670.61924	22345.13274	53915.27599	33692.72237
100	Hippurate	1192.66055	945.9815547	1089.60177	634.1463415	308.6253369
101	Histidine	66513.76147	110144.9275	39048.67257	97560.97561	39487.87062
102	Homocysteine	5768.348624	10658.76153	5464.60177	11681.64313	5471.698113
103	Homocystine	2.94800885	2.94800885	2.94800885	2.94800885	7.18328841
104	Homogentisate	85.66513761	126.4822134	111.7256637	75.35301669	82.74932615
105	Homoserine	27.52293578	28.19499341	64.93362832	116.4313222	21.56334232
106	Homoserine	54013.76147	120158.1028	52323.00885	108215.6611	77358.49057
107	Homovanillate	2029.816514	2094.86166	1935.840708	2721.437741	3045.822102
108	Hydroxykynurenine	18.34862385	14.09749671	11.83628319	20.53915276	28.70619946
109	Hydroxyproline	352064.2202	367588.9328	294247.7876	356867.7792	400269.5418
110	Hypoxanthine	285550.4587	346508.5639	265486.7257	427471.1168	241239.8922
111	IMP	15825.68807	18972.33202	6305.309735	25545.57125	12843.66577
112	Inosine	524082.5688	696969.697	463495.5752	784338.896	597035.0404
113	Inositol	85.66513761	91.43610013	94.46902655	232.3491656	100.6738544
114	Kynurenate	1433.486239	2490.118577	2400.442478	1912.708601	2587.601078
115	Kynurenine	2373.853211	3399.209486	1570.79646	3068.035944	1428.571429
116	Lactose	48.96788991	119.4993412	11.83628319	82.15661104	64.69002695
117	L-NMMA	3669.724771	8537.549407	5066.371681	9897.304236	2708.894879
118	Malate	50114.6789	122266.1397	23451.32743	89602.05392	55525.60647

119	Malonate	15596.33028	8418.972332	9568.584071	36970.47497	5970.350404
120	Malonyl-CoA	24.4266055	189.7233202	29.53539823	2.94800885	71.96765499
121	Melatonin	2878.440367	2977.602108	1880.530973	3440.308087	1415.09434
122	Metanephrine	407.1100917	322.7931489	141.5929204	379.9743261	640.1617251
123	Methionine	80619.26606	180500.6588	70132.74336	157894.7368	93261.45553
124	Methylmalonate	52178.89908	137022.3979	96460.17699	87291.39923	88948.78706
125	Mevalonate	658.2568807	1343.873518	573.0088496	965.3401797	452.8301887
126	m-Hydroxyphenylpyruvic acid	18.34862385	49.27536232	5.896017699	20.53915276	14.42048518
127	NAD	61.23853211	337.2859025	159.2920354	211.8100128	100.6738544
128	NADH	18.34862385	28.19499341	29.53539823	82.15661104	7.18328841
129	NADP	2.94800885	2.94800885	5.896017699	6.842105263	2.94800885
130	NADPH	2.94800885	2.94800885	2.94800885	2.94800885	2.94800885
131	Neopterin	2.94800885	2.94800885	11.83628319	13.73555841	2.94800885
132	Niacinamide	427752.2936	475625.8235	415929.2035	449293.9666	487870.6199
133	Nicotinate ribonucleotide	1651.376147	1646.903821	1068.584071	1951.219512	870.6199461
134	Nicotinic acid	1490.825688	4216.073781	1194.690265	3055.198973	1469.002695
135	OMP	251.146789	133.0698287	100.3318584	20.53915276	93.5309973
136	Ornithine	91.74311927	309.6179183	82.63274336	14890.88575	136.1185984
137	Orotate	568.8073394	1011.857708	513.2743363	1347.8819	644.2048518
138	Oxalate	1095.183486	794.4664032	1227.876106	1047.496791	1617.250674
139	Oxaloacetate	225.9174312	436.1001318	613.9380531	273.4274711	471.6981132
140	Oxidized glutathione	685.7798165	2081.68643	932.5221239	3632.862644	2843.665768
141	Oxoglutaric acid	4552.752294	7140.974967	1117.256637	4454.428755	2169.811321
142	Pantothenate	19495.41284	24242.42424	20685.84071	37355.58408	7991.913747
143	PEP	354.3577982	505.9288538	366.1504425	225.9306804	222.3719677
144	PGE2	220.1834862	505.9288538	173.6725664	887.0346598	258.7601078
145	Phenylalanine	355504.5872	451910.4084	344026.5487	414634.1463	455525.6065
146	Phosphoethanolamine	3933.486239	8801.054018	3915.929204	12323.49166	7601.078167
147	Phosphotyrosine	893.3486239	3478.26087	873.8938053	6341.463415	2008.086253
148	PPA	91.85779817	322.7931489	109.1814159	170.7317073	230.458221
149	Proline	417431.1927	566534.9144	379424.7788	535301.6688	451482.4798
150	Propionate	2.94800885	14.09749671	23.5619469	13.73555841	28.70619946
151	Pyridoxal 5'-phosphate	2.94800885	14.09749671	2.94800885	2.94800885	2.94800885
152	Pyridoxine	22935.77982	18313.57049	6238.938053	35173.2991	7951.48248
153	Pyruvate	61.23853211	91.43610013	129.4247788	164.3132221	86.38814016
154	Quinolinate	55.04587156	333.3333333	348.4513274	441.5917843	301.8867925
155	S-(5'-Adenosyl)-L-homocysteine	905.9633028	990.7773386	295.3539823	870.3465982	452.8301887
156	Salicylurate	422.0183486	421.6073781	100.3318584	482.6700899	265.4986523
157	Serotonin	120412.844	246376.8116	23.67256637	228498.0745	17.9245283
158	Sorbitol	1341.743119	2094.86166	1227.876106	4133.504493	956.8733154
159	Spermidine	196.1009174	3465.085639	2.94800885	8292.682927	2008.086253
160	Spermine	899.0825688	2555.99473	1382.743363	1026.957638	431.2668464
161	Sucrose	1376.146789	1989.459816	1338.495575	1925.545571	1455.525606
162	Taurine	313073.3945	404479.5784	240044.2478	397946.0847	326145.5526
163	Taurochenodeoxycholate	45183.48624	92885.37549	40597.34513	143774.0693	142857.1429
164	Taurocholate	409403.6697	815546.7721	462389.3805	790757.3813	572776.2803
165	Thiamine pyrophosphate	6.112385321	7.022397892	2.94800885	6.842105263	2.94800885
166	Threonine	19954.12844	49143.61001	20353.9823	43902.43902	29514.8248
167	Thymidine	275.2293578	534.914361	56.0840708	441.5917843	280.3234501
168	Thymine	30.61926606	35.17786561	23.67256637	17.07317073	32.34501348
169	Trimethylamine-N-oxide	10229.3578	13965.7444	7035.39823	34017.97176	14959.56873
170	Tryptophan	137614.6789	229249.0119	107964.6018	191270.8601	123315.3639
171	Tyrosine	57568.80734	117786.5613	51991.15044	96534.01797	75741.23989
172	UDP	1857.798165	2332.01581	737.8318584	1630.29525	791.1051213
173	UDP-glucose	4139.908257	9130.434783	3362.831858	12207.95892	5646.90027
174	UDP-glucuronate	532.1100917	1383.399209	761.0619469	1232.349166	913.7466307
175	UMP	42316.51376	53359.68379	24336.28319	45186.13607	28167.1159
176	Uracil	10905.9633	13702.23979	9369.469027	23234.91656	8355.795148
177	Urate	15940.36697	7417.654809	5652.654867	8729.139923	2466.307278
178	Ureidopropionic acid	2155.963303	2951.251647	2555.309735	3453.145058	3018.867925
179	Uridine	9965.59633	15151.51515	9347.345133	19897.30424	11212.93801

180	UTP	12.2706422	2.94800885	2.94800885	2.94800885	14.42048518
181	Valine	267201.8349	351778.6561	167035.3982	322207.9589	315363.8814
182	Xanthine	96100.91743	120026.3505	74557.52212	154043.6457	56603.77358
183	Xanthine	46215.59633	49538.86693	30973.45133	69704.74968	25067.38544
184	Xanthosine	8314.220183	14624.50593	8053.097345	17715.01926	5458.221024
185	Xanthurenate	6.112385321	112.516469	17.69911504	27.34274711	35.98382749
186	XMP	691.5137615	1844.532279	897.1238938	2118.100128	1347.708895
187	γ-Aminobutyrate	32798.16514	54677.20685	23119.46903	43260.5905	47978.43666
188	isocitrate	2131.474104	5117.1875	3836.978131	1775.193798	4463.937622
189	S-adenosyl-L-methionine	984.063745	4921.875	2186.878728	2674.418605	4678.362573
190	lactate	278884.4622	503906.25	318091.4513	298449.6124	662768.0312
191	succinate	87649.40239	296875	181113.3201	99612.4031	294346.9786

id	MS2 name	KO1_si	KO2_si	KO3_si	KO4_si	KO5_si
1	1-Methylhistamine	149.0401396	204.5889101	401.3303769	233.9791356	243.697479
2	2-Aminoadipate	12757.4171	11395.7935	22394.67849	13099.85097	15846.33854
3	2-Aminoisobutyric acid	50785.34031	55831.73996	90465.63193	49627.42176	50660.26411
4	2'-Deoxyadenosine	605.5846422	959.8470363	758.3148559	707.8986587	986.7947179
5	2'-Deoxycytidine	1340.314136	1346.080306	1042.128603	867.3621461	1236.494598
6	2'-Deoxyuridine	1815.008726	2447.418738	2017.738359	1439.642325	877.5510204
7	2-Hydroxy-2-methylbutyric acid	493.8917976	397.7055449	472.2838137	572.2801788	409.3637455
8	3-Hydroxyanthranilic acid	619546.2478	554493.3078	662971.1752	533532.0417	392557.0228
9	3-Nitrotyrosine	3019.197208	1648.183556	4146.341463	2369.597615	1764.705882
10	3-Phosphoglycerate	149.0401396	142.8298279	59.20177384	79.58271237	41.65666267
11	4-Hydroxy-3-methoxyphenylglyc	27.92321117	30.59273423	23.72505543	23.84500745	12.84513806
12	4-Hydroxybenzoate	8516.579407	14760.99426	34368.07095	11385.99106	7575.030012
13	4-Pyridoxic acid	4450.26178	2619.502868	5920.177384	3695.976155	1944.777911
14	5-Hydroxyindole-3-acetate	2094.240838	3690.248566	4501.108647	2652.757079	2605.042017
15	5-Hydroxytryptophan	74.52006981	673.040153	241.6851441	1232.488823	22.44897959
16	5-Methyltetrahydrofolic acid	83.76963351	102.1032505	94.67849224	95.38002981	147.6590636
17	Acetoacetate	7975.56719	4168.260038	6252.771619	14456.03577	11776.71068
18	Acetylcholine	485165.7941	304015.2964	696230.5987	396423.2489	316926.7707
19	Acetyl-CoA	102.443281	265.7743786	365.8536585	150.5216095	96.03841537
20	Aconitate	2792.321117	2715.105163	3348.115299	2146.050671	2076.830732
21	Adenine	1954.624782	1162.523901	957.8713969	1216.09538	1031.212485
22	Adenosine	4083.769634	3518.164436	1443.45898	3278.688525	13925.57023
23	Adenosine	469458.9878	407265.7744	257206.2084	393442.623	340936.3745
24	Adenylosuccinate	176.2652705	745.6978967	982.2616408	898.6587183	178.8715486
25	ADMA	37.34729494	51.05162524	47.22838137	87.48137109	51.2605042
26	ADP	2931.937173	4282.982792	5698.447894	3681.073025	4021.608643
27	Alanine	884816.7539	915869.9809	937915.7428	919523.0999	740696.2785
28	Allantoin	1228.621291	877.6290631	2771.618625	1579.731744	1932.773109
29	AMP	91797.55672	151242.8298	221729.49	85245.90164	158463.3854
30	Anthranilate	10314.13613	9082.217973	11130.8204	10208.64382	6686.67467
31	Arginine	270.5061082	801.1472275	147.8935698	250.3725782	159.6638655
32	Argininosuccinate	1493.891798	449.3307839	1290.465632	1137.108793	1452.581032
33	Ascorbate	93.19371728	40.91778203	118.4035477	31.89269747	11848.7395
34	Asparagine	50959.86038	58317.39962	31263.85809	46944.85842	37454.98199
35	Aspartate	22338.56894	38623.32696	20665.18847	18777.94337	9783.913565
36	ATP	9.301919721	10.19120459	11.81818182	15.94634873	19.20768307
37	Betaine	970331.5881	1829827.916	1261640.798	900149.0313	695078.0312
38	Bilirubin	1666.666667	1214.14914	1407.982262	1803.278689	717.8871549
39	Biotin	25305.41012	16022.94455	14301.55211	26080.4769	22448.97959
40	cAMP	577.6614311	969.4072658	982.2616408	500.7451565	806.7226891
41	Carnitine	221640.4887	183747.6099	277161.8625	192250.3726	154861.9448
42	Carnosine	16841.18674	7380.497132	23059.86696	12921.01341	10432.17287
43	CDP	2.94800885	2.94800885	2.94800885	15.94634873	2.94800885
44	cGMP	18.67364747	20.45889101	82.92682927	2.94800885	6.398559424
45	Chenodeoxycholate	55.84642234	112.2370937	94.67849224	87.48137109	64.10564226
46	Cholate	10732.98429	9024.856597	60088.6918	14754.09836	8463.385354
47	Citrate	153.7521815	40.72657744	5144.124169	5499.254844	86.43457383
48	Citrulline	28795.81152	45315.48757	36807.09534	36363.63636	38655.46218
49	CMP	15078.53403	16099.42639	23725.05543	16244.41133	18487.39496
50	Cobalamin	315.8813264	785.8508604	687.3614191	1320.417288	717.8871549
51	Cotinine	3036.649215	2791.586998	6851.441242	3353.204173	2448.979592
52	Creatine	996509.5986	994263.8623	1203991.131	825633.383	709483.7935

53	Creatinine	326352.5305	204588.9101	374722.8381	323397.9136	291716.6867
54	CTP	2.94800885	10.19120459	11.81818182	7.943368107	12.84513806
55	Cystamine	65.27050611	142.8298279	141.9068736	87.48137109	121.2484994
56	Cystathionine	65.27050611	152.9636711	106.4301552	95.38002981	19.20768307
57	Cysteamine	4729.493892	3499.043977	5942.350333	4217.585693	4225.690276
58	Cysteine	726.0034904	847.0363289	674.0576497	636.3636364	499.3997599
59	Cytidine	28621.29145	26386.23327	14168.51441	37853.94933	52941.17647
60	Cytosine	27.92321117	61.18546845	153.8802661	87.48137109	44.89795918
61	DCDP	5479.930192	6195.028681	3259.423503	4769.00149	5930.372149
62	DCMP	652.7050611	684.5124283	1787.13969	429.2101341	883.5534214
63	DCTP	2.94800885	10.19120459	2.94800885	7.943368107	6.398559424
64	D-Erythrose 4-phosphate	1535.776614	1449.330784	2093.126386	1391.95231	1006.002401
65	D-Fructose 1,6-bisphosphate	3403.141361	1489.483748	3126.385809	3025.33532	2136.854742
66	D-Fructose 2,6-bisphosphate	46.59685864	112.2370937	485.5875831	87.48137109	76.83073229
67	D-Glucuronic acid	5916.230366	4091.778203	3414.634146	8196.721311	6326.530612
68	D-Glycerate 2-phosphate	4659.685864	2619.502868	2372.505543	5275.707899	7875.15006
69	DHAP	204.1884817	305.9273423	638.5809313	684.0536513	1836.734694
70	Dihydrofolate	9.301919721	2.94800885	23.72505543	7.943368107	2.94800885
71	Dimethyl glycine	149.0401396	127.91587	496.6740576	63.63636364	51.2605042
72	Dopamine	193717.2775	151434.0344	177383.592	183308.4948	133253.3013
73	D-Ribulose 5-phosphate	3630.017452	5793.499044	8957.871397	4247.391952	6254.501801
74	DTMP	167.7137871	1009.560229	1277.161863	827.123696	691.4765906
75	DTTP	27.92321117	61.18546845	94.67849224	79.58271237	83.31332533
76	DUMP	3769.633508	730.4015296	3392.461197	2563.338301	294.1176471
77	DUTP	37.17277487	20.45889101	23.72505543	23.84500745	12.84513806
78	Epinephrine	2233.856894	2868.068834	2099.778271	2354.694486	3061.22449
79	Folate	9.301919721	10.19120459	2.94800885	39.79135618	12.84513806
80	Folinate	18.67364747	2.94800885	2.94800885	15.94634873	12.84513806
81	Fructose	664921.466	552581.262	878048.7805	652757.079	749099.6399
82	Fructose 1-phosphate	54101.22164	27915.86998	87361.41907	45454.54545	75390.15606
83	Fumarate	5654.450262	10630.97514	11042.1286	8479.880775	7286.914766
84	GDP	764.3979058	1367.112811	1751.662971	512.6676602	819.9279712
85	Geranyl pyrophosphate	59336.82373	59847.03633	137028.8248	39642.32489	17887.15486
86	Glucose 1-phosphate	36474.69459	15200.76482	62749.44568	29806.25931	39735.89436
87	Glutamate	296684.1187	326959.847	332594.235	260804.769	184873.9496
88	Glutamine	312390.925	330783.9388	399113.082	281669.1505	211284.5138
89	Glutathione	41361.25654	38814.53155	35254.98891	27570.78987	83793.51741
90	Glyceraldehyde	884.8167539	284.8948375	1560.97561	938.8971684	963.9855942
91	Glycerol	83.76963351	61.18546845	59.20177384	47.6900149	44.77791116
92	Glycerol-3-phosphate	17975.56719	27915.86998	70509.97783	15797.31744	8871.548619
93	Glycine	60907.50436	52390.05736	75166.29712	60059.61252	51380.55222
94	Glycochenodeoxycholate	37.34729494	71.31931166	11.81818182	71.53502235	38.41536615
95	Glycocholate	977.3123909	4091.778203	294.9002217	1803.278689	1656.662665
96	GMP	10174.52007	16883.3652	36141.90687	13308.49478	20408.16327
97	GTP	2.94800885	20.45889101	2.94800885	2.94800885	2.94800885
98	Guanine	6474.69459	6195.028681	4966.740576	8211.624441	15366.14646
99	Guanosine	58115.18325	58317.39962	38137.47228	64381.52012	123649.4598
100	Hippurate	279.2321117	61.18546845	603.1042129	143.0700447	339.7358944
101	Histidine	179755.6719	307839.3881	252771.6186	295081.9672	231692.6771
102	Homocysteine	5130.890052	5277.246654	5898.004435	7481.371088	4201.680672
103	Homocystine	2.94800885	10.19120459	2.94800885	2.94800885	2.94800885
104	Homogentisate	121.1169284	61.18546845	212.8603104	31.74366617	195.6782713
105	Homoserine	74.52006981	91.77820268	141.9068736	223.5469449	15.96638655
106	Homoserine	249563.6998	281070.7457	254988.9135	204172.8763	135654.2617
107	Homovanillate	2.94800885	3097.51434	3614.190687	2548.435171	1296.518607
108	Hydroxykynurenine	27.92321117	30.59273423	59.20177384	23.84500745	12.84513806
109	Hydroxyproline	478184.9913	497131.9312	647450.1109	442622.9508	360144.0576
110	Hypoxanthine	623036.6492	544933.0784	623059.867	560357.6751	438175.2701
111	IMP	11291.44852	33269.59847	27716.18625	10774.96274	29411.76471
112	Inosine	774869.1099	856596.5583	643015.5211	761549.9255	683073.2293
113	Inositol	186.7364747	122.3709369	70.95343681	71.53502235	51.2605042
114	Kynurenate	65.09598604	66.34799235	70.95343681	23.84500745	25.57022809
115	Kynurenine	2164.048866	1856.596558	1940.133038	1579.731744	1096.038415
116	Lactose	130.3664921	10.19120459	106.4301552	103.4277198	96.03841537
117	L-NMMA	18.67364747	71.31931166	94.67849224	15.94634873	2.94800885
118	Malate	193717.2775	193116.6348	279379.1574	210134.1282	182472.9892
119	Malonate	25828.97033	89101.33843	150110.8647	100000	115966.3866
120	Malonyl-CoA	9.301919721	10.19120459	118.4035477	15.94634873	6.398559424
121	Melatonin	1155.322862	1847.036329	15454.54545	1728.76304	2496.9988
122	Metanephrine	343.8045375	51.05162524	171.6186253	397.9135618	474.1896759
123	Methionine	244328.0977	208413.0019	381374.7228	283159.4635	250900.3601

124	Methylmalonate	2.94800885	2.94800885	2.94800885	1216.09538	57.62304922
125	Mevalonate	1745.200698	1357.552581	4279.379157	1669.150522	1824.729892
126	m-Hydroxyphenylpyruvic acid	410.122164	30.59273423	1290.465632	286.1400894	236.4945978
127	NAD	195.4624782	346.0803059	259.4235033	222.0566319	211.2845138
128	NADH	18.67364747	40.91778203	59.20177384	23.84500745	89.67587035
129	NADP	2.94800885	2.94800885	2.94800885	2.94800885	2.94800885
130	NADPH	2.94800885	2.94800885	2.94800885	2.94800885	12.84513806
131	Neopterin	9.301919721	20.45889101	2.94800885	2.94800885	12.84513806
132	Niacinamide	647469.459	694072.6577	802660.7539	560357.6751	490996.3986
133	Nicotinate ribonucleotide	2425.82897	3365.200765	4545.454545	2876.304024	2304.921969
134	Nicotinic acid	5863.874346	6577.437859	9423.503326	4798.80775	1019.207683
135	OMP	37.17277487	91.77820268	223.9467849	71.53502235	128.4513806
136	Ornithine	237.3472949	91.77820268	94.67849224	233.9791356	76.83073229
137	Orotate	1373.472949	1009.560229	769.4013304	1359.165425	806.7226891
138	Oxalate	1630.017452	1673.040153	2064.301552	1137.108793	1031.212485
139	Oxaloacetate	410.122164	428.2982792	390.2439024	421.7585693	410.5642257
140	Oxidized glutathione	23560.20942	39961.75908	59201.77384	35171.38599	50180.07203
141	Oxoglutaric acid	14642.23386	12562.14149	18802.66075	12861.40089	12364.94598
142	Pantothenate	59336.82373	23518.16444	29046.56319	47540.98361	20648.2593
143	PEP	828.9703316	887.1892925	1844.789357	731.7436662	537.8151261
144	PGE2	93.19371728	1931.166348	35.4767184	39.79135618	420.1680672
145	Phenylalanine	603839.4415	642447.4187	749445.6763	549925.4844	452581.0324
146	Phosphoethanolamine	9738.219895	17934.99044	42793.79157	19225.03726	25570.22809
147	Phosphotyrosine	1972.076789	1061.185468	674.0576497	588.6736215	2400.960384
148	PPA	577.6614311	235.1816444	7516.629712	1034.277198	717.8871549
149	Proline	774869.1099	822179.7323	929046.5632	661698.9568	524609.8439
150	Propionate	2.94800885	2.94800885	2.94800885	2.94800885	2.94800885
151	Pyridoxal 5'-phosphate	9.301919721	2.94800885	2.94800885	2.94800885	2.94800885
152	Pyridoxine	20767.88831	20267.68642	37915.74279	30551.4158	54861.94478
153	Pyruvate	340.3141361	208.4130019	189.3569845	103.4277198	128.4513806
154	Quinolinate	102.443281	1122.370937	521.0643016	891.2071535	1992.797119
155	S-(5'-Adenosyl)-L-homocysteine	586.3874346	1214.14914	946.7849224	731.7436662	723.8895558
156	Salicylurate	260.034904	1223.709369	691.7960089	652.757079	339.7358944
157	Serotonin	37.17277487	35.75525813	47.22838137	15.94634873	19.20768307
158	Sorbitol	8516.579407	6003.824092	11197.33925	8614.008942	12124.84994
159	Spermidine	1099.47644	2.94800885	1301.552106	2.94800885	650.6602641
160	Spermine	1331.588133	305.9273423	1372.505543	879.2846498	378.1512605
161	Sucrose	2757.417103	2256.214149	3281.596452	2295.081967	2533.013205
162	Taurine	401396.1606	252390.0574	596452.3282	380029.8063	331332.533
163	Taurochenodeoxycholate	51657.94066	38049.71319	3858.093126	36363.63636	27370.94838
164	Taurocholate	835951.1344	216061.1855	243902.439	508196.7213	338535.4142
165	Thiamine pyrophosphate	18.67364747	10.19120459	2.94800885	2.94800885	12.84513806
166	Threonine	100349.0401	113957.935	100886.918	87332.33979	56902.7611
167	Thymidine	1638.743455	1437.858509	3658.536585	1713.859911	1236.494598
168	Thymine	27.92321117	71.51051625	47.22838137	31.74366617	32.05282113
169	Trimethylamine-N-oxide	2076.788831	23135.75526	1088.691796	7988.077496	17887.15486
170	Tryptophan	336823.7347	302103.2505	470066.5188	371087.9285	325330.1321
171	Tyrosine	150436.3002	181835.5641	207982.2616	214605.0671	169267.7071
172	UDP	1275.74171	1244.741874	2070.953437	588.6736215	960.3841537
173	UDP-glucose	3472.949389	6634.799235	19223.94678	3368.107303	6158.463385
174	UDP-glucuronate	652.7050611	2466.539197	4833.702882	612.5186289	1788.715486
175	UMP	39092.49564	52963.67113	88470.06652	41132.63785	48379.35174
176	Uracil	30541.01222	16347.99235	24833.70288	26527.57079	11344.53782
177	Urate	9144.851658	24856.59656	17516.62971	10700.44709	11572.62905
178	Ureidopropionic acid	3368.237347	3460.803059	3636.363636	3517.138599	3193.277311
179	Uridine	39965.09599	24282.98279	31707.31707	45156.48286	38295.31813
180	UTP	37.17277487	2.94800885	11.81818182	2.94800885	2.94800885
181	Valine	438045.3752	449330.7839	552106.4302	350223.5469	272509.0036
182	Xanthine	107504.363	93499.04398	97339.24612	133532.0417	71548.61945
183	Xanthine	49040.13962	43212.23709	45232.81596	60655.7377	36734.69388
184	Xanthosine	5008.726003	3154.875717	2882.48337	6646.795827	1788.715486
185	Xanthurenate	167.539267	265.7743786	319.2904656	286.1400894	205.2821128
186	XMP	1610.820244	2829.827916	4833.702882	2190.76006	3565.42617
187	γ-Aminobutyrate	110645.7243	125047.8011	189800.4435	114903.1297	99879.95198
188	isocitrate	1246.464646	524.5579568	39.92248062	2.94800885	1157.464213
189	S-adenosyl-L-methionine	2646.464646	1738.70334	139.5348837	20.89249493	3415.132924
190	lactate	458585.8586	223968.5658	298.4496124	32657.20081	437627.8119
191	succinate	64444.44444	36149.31238	1534.883721	1929.006085	60736.19632

id	MS2 name	WT1_co	WT2_co	WT3_co	WT4_co	WT5_co
1	1-Methylhistamine	754.789272	1874.34555	837.5634518	284.6975089	667.953668
2	2-Aminoadipate	7892.720307	7225.13089	2710.659898	4377.224199	6525.096525
3	2-Aminoisobutyric acid	26436.78161	32931.93717	20761.42132	31814.94662	21467.18147
4	2'-Deoxyadenosine	632.183908	979.0575916	1598.984772	1064.05694	513.5135135
5	2'-Deoxycytidine	3701.149425	1842.931937	3563.451777	2829.181495	3706.563707
6	2'-Deoxyuridine	4022.988506	5863.874346	7817.258883	4448.398577	5289.57529
7	2-Hydroxy-2-methylbutyric acid	754.789272	643.9790576	705.5837563	398.5765125	760.6177606
8	3-Hydroxyanthranilic acid	1122605.364	1403141.361	1269035.533	1046263.345	1196911.197
9	3-Nitrotyrosine	1716.475096	696.3350785	1137.055838	854.0925267	884.1698842
10	3-Phosphoglycerate	204.5977011	335.078534	203.0456853	151.9572954	226.6409266
11	4-Hydroxy-3-methoxyphenylglyc	40.99616858	56.02094241	27.05583756	38.07829181	41.31274131
12	4-Hydroxybenzoate	2145.59387	4162.303665	4928.93401	1348.754448	7567.567568
13	4-Pyridoxic acid	10498.08429	6492.146597	3208.121827	7046.263345	5289.57529
14	5-Hydroxyindole-3-acetate	81.6091954	111.5183246	81.21827411	113.8790036	103.0888031
15	5-Hydroxytryptophan	521.0727969	223.5602094	1274.111675	85.40925267	2648.648649
16	5-Methyltetrahydrofolic acid	265.9003831	223.5602094	27.05583756	95.01779359	123.5521236
17	Acetoacetate	142.9118774	195.8115183	162.4365482	275.4448399	123.5521236
18	Acetylcholine	934865.9004	1036649.215	1314720.812	893238.4342	737451.7375
19	Acetyl-CoA	347.5095785	251.3089005	352.284264	322.7758007	185.3281853
20	Aconitate	2352.490421	1240.837696	1923.857868	1348.754448	1494.208494
21	Adenine	2176.245211	6335.078534	4441.624365	4839.857651	3274.131274
22	Adenosine	409.9616858	307.3298429	189.8477157	265.8362989	413.1274131
23	Adenosine	70114.94253	87434.55497	52791.87817	67259.78648	48262.54826
24	Adenylosuccinate	24252.87356	85340.31414	32182.74112	21779.35943	33976.83398
25	ADMA	39080.45977	83.76963351	54.31472081	303.9145907	82.62548263
26	ADP	6321.83908	8219.895288	8375.634518	4590.747331	5791.505792
27	Alanine	455938.6973	602094.2408	424873.0964	437722.4199	478764.4788
28	Allantoin	1206.896552	1115.183246	1137.055838	967.9715302	907.3359073
29	AMP	173180.0766	186387.4346	196446.7005	144839.8577	154054.0541
30	Anthranilate	10804.5977	13926.70157	9847.715736	11814.94662	11698.8417
31	Arginine	377777.7778	3869.109948	918.7817259	494.6619217	575.2895753
32	Argininosuccinate	265.9003831	502.617801	512.6903553	494.6619217	617.7606178
33	Ascorbate	265.9003831	195.8115183	2736.040609	494.6619217	257.5289575
34	Asparagine	16934.8659	26073.29843	13857.86802	14128.11388	23050.19305
35	Aspartate	145210.728	174869.1099	120812.1827	115658.363	173745.1737
36	ATP	20.42145594	112.0418848	108.1218274	38.07829181	103.0888031
37	Betaine	3444444.444	4392670.157	4091370.558	3007117.438	3467181.467
38	Bilirubin	551.7241379	2178.010471	624.3654822	1387.900356	969.1119691
39	Biotin	11992.33716	23560.20942	8172.588832	19679.7153	14324.32432
40	cAMP	754.789272	1450.26178	593.9086294	18.96797153	945.9459459
41	Carnitine	386973.1801	396335.0785	394416.2437	313523.1317	424710.4247
42	Carnosine	1084.291188	1172.774869	1233.502538	1081.850534	760.6177606
43	CDP	2.94800885	2.94800885	2.94800885	2.94800885	2.94800885
44	cGMP	40.99616858	56.02094241	27.05583756	2.94800885	20.57915058
45	Chenodeoxycholate	102.2988506	111.5183246	27.05583756	190.0355872	82.23938224
46	Cholate	347.5095785	1282.722513	406.0913706	2128.113879	513.5135135
47	Citrate	2.94800885	25026.17801	20913.70558	17188.6121	11428.57143
48	Citrulline	11685.82375	12984.29319	6954.314721	8078.291815	6911.196911
49	CMP	20306.51341	18638.74346	16345.17766	14875.44484	16486.48649
50	Cobalamin	204.5977011	670.1570681	837.5634518	683.2740214	1235.521236
51	Cotinine	6245.210728	7120.418848	16700.50761	8434.163701	11467.18147
52	Creatine	2038314.176	2575916.23	2690355.33	1886120.996	2154440.154
53	Creatinine	398467.433	348691.0995	680203.0457	608540.9253	463320.4633
54	CTP	20.42145594	2.94800885	27.05583756	2.94800885	20.57915058
55	Cystamine	265.9003831	251.3089005	216.751269	76.15658363	113.5135135
56	Cystathionine	142.9118774	223.5602094	189.8477157	190.0355872	123.5521236
57	Cysteamine	3517.241379	6387.434555	5329.949239	3245.551601	3544.401544
58	Cysteine	286.2068966	363.3507853	162.4365482	341.9928826	267.5675676
59	Cytidine	36436.78161	34450.26178	32436.54822	42704.62633	54826.25483
60	Cytosine	6053.639847	42931.93717	10203.04569	16619.21708	185.3281853
61	DCDP	8122.605364	15183.24607	10000	10284.69751	12007.72201
62	DCMP	1249.042146	1790.575916	1243.654822	1975.088968	2328.185328
63	DCTP	2.94800885	2.94800885	2.94800885	18.96797153	2.94800885
64	D-Erythrose 4-phosphate	2659.003831	2219.895288	3197.969543	4021.352313	4362.934363
65	D-Fructose 1,6-bisphosphate	2739.463602	3947.643979	2619.28934	4946.619217	5598.455598
66	D-Fructose 2,6-bisphosphate	265.9003831	474.8691099	512.6903553	151.9572954	164.8648649
67	D-Glucuronic acid	1061.302682	1481.675393	1274.111675	814.9466192	698.8416988
68	D-Glycerate 2-phosphate	61.30268199	643.9790576	162.4365482	132.7402135	185.3281853
69	DHAP	1390.804598	696.3350785	1898.477157	814.9466192	803.0888031
70	Dihydrofolate	2.94800885	2.94800885	2.94800885	2.94800885	41.31274131

71	Dimethyl glycine	471.2643678	307.3298429	297.9695431	359.430605	309.2664093
72	Dopamine	198084.2912	265968.5864	200507.6142	212099.6441	228571.4286
73	D-Ribulose 5-phosphate	10459.77011	15235.60209	10710.6599	8256.227758	7915.057915
74	DTMP	286.2068966	363.3507853	297.9695431	683.2740214	722.007722
75	DTTP	347.5095785	279.5811518	108.1218274	75.80071174	20.57915058
76	DUMP	8544.061303	13926.70157	9644.670051	6619.217082	8416.988417
77	DUTP	61.30268199	111.5183246	54.31472081	18.96797153	41.31274131
78	Epinephrine	2432.950192	2670.157068	2736.040609	1633.451957	2749.034749
79	Folate	2.94800885	2.94800885	2.94800885	2.94800885	20.57915058
80	Folinate	40.99616858	2.94800885	2.94800885	18.96797153	2.94800885
81	Fructose	66666.66667	31518.32461	53299.49239	49466.19217	32200.7722
82	Fructose 1-phosphate	78160.91954	123560.2094	96954.31472	131672.5979	171428.5714
83	Fumarate	6130.268199	18952.87958	1827.411168	7153.024911	1722.007722
84	GDP	1310.344828	1282.722513	1649.746193	740.2135231	988.4169884
85	Geranyl pyrophosphate	2616.858238	2654.450262	1436.548223	1405.69395	1729.72973
86	Glucose 1-phosphate	63984.67433	104712.0419	74619.28934	118505.3381	130888.0309
87	Glutamate	551724.1379	701570.6806	736040.6091	516014.2349	621621.6216
88	Glutamine	324137.931	441361.2565	352284.264	387900.3559	362548.2625
89	Glutathione	98084.29119	123560.2094	85279.18782	84341.63701	91119.69112
90	Glyceraldehyde	204.5977011	167.539267	189.8477157	246.975089	371.042471
91	Glycerol	143.2950192	307.3298429	243.6548223	190.0355872	185.3281853
92	Glycerol-3-phosphate	109961.6858	86910.99476	104568.5279	73665.48043	121621.6216
93	Glycine	27662.83525	34240.8377	27055.83756	25231.31673	32355.21236
94	Glycochenodeoxycholate	40.99616858	2.94800885	54.31472081	18.96797153	20.57915058
95	Glycocholate	163.6015326	83.76963351	54.31472081	75.80071174	2.94800885
96	GMP	42145.59387	50209.42408	52284.26396	39501.77936	44015.44402
97	GTP	20.42145594	2.94800885	81.21827411	2.94800885	2.94800885
98	Guanine	4329.501916	5497.382199	3736.040609	6868.327402	4671.814672
99	Guanosine	27854.40613	45078.53403	32487.30964	57651.24555	37104.2471
100	Hippurate	1452.10728	1732.984293	791.8781726	494.6619217	513.5135135
101	Histidine	76628.35249	105759.1623	97461.92893	66903.91459	105019.305
102	Homocysteine	1923.371648	2376.963351	1324.873096	3056.939502	2760.617761
103	Homocystine	2.94800885	2.94800885	27.05583756	2.94800885	2.94800885
104	Homogentisate	316.8582375	251.3089005	189.8477157	142.3487544	309.2664093
105	Homoserine	183.908046	56.02094241	189.8477157	95.01779359	185.7142857
106	Homoserine	126436.7816	191099.4764	98984.77157	111743.7722	136293.4363
107	Homovanillate	2.94800885	10104.71204	11675.1269	2.94800885	5830.11583
108	Hydroxykynurenine	20.42145594	56.02094241	81.21827411	95.01779359	41.31274131
109	Hydroxyproline	421455.9387	790575.9162	482233.5025	537366.548	621621.6216
110	Hypoxanthine	720306.5134	884816.7539	685279.1878	743772.242	818532.8185
111	IMP	179310.3448	119371.7277	130964.467	54804.27046	154440.1544
112	Inosine	1007662.835	2151832.461	1208121.827	1473309.609	1169884.17
113	Inositol	347.5095785	307.3298429	379.1878173	113.8790036	226.6409266
114	Kynurenate	785.440613	753.9267016	2086.294416	95.01779359	41.31274131
115	Kynurenine	1739.463602	2429.319372	1979.695431	2647.686833	1752.895753
116	Lactose	102.2988506	111.5183246	81.21827411	76.15658363	41.31274131
117	L-NMMA	6398.467433	9895.287958	5279.187817	170.8185053	82.23938224
118	Malate	104214.5594	309947.644	48274.11168	149822.0641	433590.7336
119	Malonate	15708.81226	31361.25654	30304.56853	73665.48043	28880.30888
120	Malonyl-CoA	102.2988506	528.7958115	325.3807107	133.0960854	206.1776062
121	Melatonin	45593.86973	50785.34031	25329.94924	53380.78292	20154.44015
122	Metanephrine	81.6091954	41.93717277	135.5329949	76.15658363	123.5521236
123	Methionine	148275.8621	244502.6178	114213.198	134519.573	151351.3514
124	Methylmalonate	39846.7433	43874.34555	43502.53807	2.94800885	307.7220077
125	Mevalonate	7049.808429	7172.774869	5634.517766	8362.989324	8030.888031
126	m-Hydroxyphenylpyruvic acid	61.30268199	139.7905759	81.21827411	38.07829181	41.31274131
127	NAD	143.2950192	307.3298429	487.8172589	246.975089	144.015444
128	NADH	2.94800885	56.02094241	54.31472081	18.96797153	41.31274131
129	NADP	2.94800885	2.94800885	2.94800885	2.94800885	20.57915058
130	NADPH	40.99616858	56.02094241	2.94800885	18.96797153	2.94800885
131	Neopterin	2.94800885	2.94800885	81.21827411	2.94800885	20.57915058
132	Niacinamide	1137931.034	1507853.403	1309644.67	982206.4057	1077220.077
133	Nicotinate ribonucleotide	4789.272031	8848.167539	5431.472081	3341.637011	5212.355212
134	Nicotinic acid	9885.057471	45235.60209	44314.72081	129537.3665	66795.3668
135	OMP	448.2758621	418.8481675	837.5634518	322.7758007	679.5366795
136	Ornithine	570.8812261	265.4450262	338.5786802	608.5409253	575.2895753
137	Orotate	2015.32567	2879.581152	1192.893401	2042.704626	2050.19305
138	Oxalate	3659.003831	5287.958115	6345.177665	3950.177936	4131.274131
139	Oxaloacetate	1678.16092	3717.277487	338.5786802	818.5053381	463.3204633
140	Oxidized glutathione	18275.86207	21465.96859	27411.16751	39145.90747	37683.39768
141	Oxoglutaric acid	16168.58238	25078.53403	8629.441624	871.886121	7799.227799

142	Pantothenate	9540.229885	16806.28272	10862.94416	12206.40569	11853.28185
143	PEP	1574.712644	3267.015707	3304.568528	1309.608541	1069.498069
144	PGE2	3701.149425	4890.052356	8324.873096	5053.380783	2142.857143
145	Phenylalanine	858237.5479	1450261.78	847715.736	882562.2776	1054054.054
146	Phosphoethanolamine	20574.71264	20837.69634	27918.78173	28291.81495	32625.48263
147	Phosphotyrosine	1655.172414	3769.633508	1517.766497	950.1779359	1938.223938
148	PPA	306.51341	447.1204188	568.5279188	227.7580071	308.8803089
149	Proline	1180076.628	1581151.832	1218274.112	996441.2811	1158301.158
150	Propionate	2.94800885	2.94800885	2.94800885	2.94800885	2.94800885
151	Pyridoxal 5'-phosphate	2.94800885	2.94800885	2.94800885	2.94800885	2.94800885
152	Pyridoxine	34482.75862	34240.8377	27512.69036	21672.59786	32586.87259
153	Pyruvate	276.2452107	335.078534	365.9898477	275.4448399	247.1042471
154	Quinolinate	2574.712644	1534.031414	1233.502538	1519.572954	2389.96139
155	S-(5'-Adenosyl)-L-homocysteine	754.789272	1926.701571	812.1827411	1195.729537	945.9459459
156	Salicylurate	1532.56705	1256.544503	487.3096447	398.5765125	752.8957529
157	Serotonin	50.95785441	139.7905759	54.31472081	95.01779359	82.23938224
158	Sorbitol	961.6858238	612.565445	1081.218274	814.9466192	451.7374517
159	Spermidine	26628.35249	4246.073298	25431.47208	1348.754448	2.94800885
160	Spermine	735.6321839	181.6753927	1111.675127	341.9928826	389.96139
161	Sucrose	3088.122605	2178.010471	3223.350254	1861.209964	1876.447876
162	Taurine	750957.8544	910994.7644	832487.3096	701067.6157	861003.861
163	Taurochenodeoxycholate	7509.578544	14764.39791	4172.588832	11067.61566	7606.177606
164	Taurocholate	60536.39847	109947.644	33705.58376	120640.5694	59459.45946
165	Thiamine pyrophosphate	20.42145594	112.0418848	2.94800885	18.96797153	20.57915058
166	Threonine	47509.57854	69109.94764	36497.46193	47686.83274	53281.85328
167	Thymidine	2984.67433	3298.429319	1583.756345	1651.245552	1482.625483
168	Thymine	122.605364	111.5183246	135.5329949	133.0960854	41.31274131
169	Trimethylamine-N-oxide	29578.54406	41780.10471	75634.51777	70462.63345	37220.07722
170	Tryptophan	179310.3448	258115.1832	163451.7766	170106.7616	182239.3822
171	Tyrosine	84291.18774	141884.8168	79187.81726	86120.99644	107722.0077
172	UDP	3107.279693	3492.146597	3035.532995	1387.900356	2038.610039
173	UDP-glucose	35478.9272	39424.08377	24720.81218	11601.42349	25521.23552
174	UDP-glucuronate	3149.425287	5198.95288	6091.370558	3551.601423	3938.223938
175	UMP	75095.78544	58638.74346	90862.94416	58362.98932	55984.55598
176	Uracil	26091.95402	29842.93194	23502.53807	22811.3879	28069.49807
177	Urate	6015.32567	3492.146597	8477.15736	3341.637011	3440.15444
178	Ureidopropionic acid	6015.32567	7225.13089	7106.598985	5587.188612	6911.196911
179	Uridine	22835.24904	45340.31414	15786.80203	37722.41993	25675.67568
180	UTP	2.94800885	27.90575916	2.94800885	2.94800885	2.94800885
181	Valine	264367.8161	570680.6283	363959.3909	412811.3879	467181.4672
182	Xanthine	75478.9272	93717.27749	73604.06091	75088.96797	85714.28571
183	Xanthine	28965.51724	43874.34555	31776.64975	35551.60142	34285.71429
184	Xanthosine	3272.030651	3769.633508	3304.568528	2391.459075	4555.984556
185	Xanthurenate	40.99616858	56.02094241	54.31472081	38.07829181	41.31274131
186	XMP	9080.45977	12513.08901	9949.238579	6654.80427	11312.74131
187	γ-Aminobutyrate	60919.54023	76963.35079	47309.64467	70106.76157	47490.34749
188	isocitrate	3851.612903	2093.023256	898.6175115	878.3783784	2193.675889
189	S-adenosyl-L-methionine	18903.22581	4726.744186	8433.179724	5225.225225	6047.43083
190	lactate	838709.6774	387209.3023	520737.3272	463963.964	403162.0553
191	succinate	303225.8065	91860.46512	43640.553	59909.90991	101581.0277

id	MS2 name	KO1_co	KO2_co	KO3_co	KO4_co	KO5_co
1	1-Methylhistamine	1176.165803	612.565445	886.1386139	731.4285714	1362.962963
2	2-Aminoadipate	28497.40933	18481.67539	14554.45545	18628.57143	10962.96296
3	2-Aminoisobutyric acid	180310.8808	175392.6702	91089.10891	211428.5714	89629.62963
4	2'-Deoxyadenosine	911.9170984	1089.005236	1133.663366	1891.428571	911.1111111
5	2'-Deoxycytidine	10000	2680.628272	1321.782178	2714.285714	2037.037037
6	2'-Deoxyuridine	4756.476684	6178.010471	2668.316832	5828.571429	9259.259259
7	2-Hydroxy-2-methylbutyric acid	442.4870466	811.5183246	871.2871287	731.4285714	792.5925926
8	3-Hydroxyanthranilic acid	922279.7927	1188481.675	841584.1584	1171428.571	1207407.407
9	3-Nitrotyrosine	4176.165803	5235.602094	1613.861386	6400	3081.481481
10	3-Phosphoglycerate	179.7927461	307.3298429	132.1782178	274.2857143	237.037037
11	4-Hydroxy-3-methoxyphenylglyc	55.44041451	27.90575916	52.97029703	91.42857143	197.7777778
12	4-Hydroxybenzoate	7927.46114	19685.86387	19356.43564	23542.85714	10962.96296
13	4-Pyridoxic acid	6165.803109	5141.361257	4638.613861	5045.714286	5807.407407
14	5-Hydroxyindole-3-acetate	2487.046632	2712.041885	2905.940594	1737.142857	2925.925926
15	5-Hydroxytryptophan	138.3419689	195.8115183	579.2079208	305.1428571	651.8518519
16	5-Methyltetrahydrofolic acid	248.7046632	83.76963351	105.9405941	30.45714286	79.25925926
17	Acetoacetate	4699.481865	251.3089005	2455.445545	1462.857143	1859.259259
18	Acetylcholine	1699481.865	685863.8743	2346534.653	788571.4286	977777.7778

19	Acetyl-CoA	497.9274611	586.3874346	211.3861386	1097.142857	237.037037
20	Aconitate	4476.683938	5287.958115	4069.306931	5942.857143	5533.333333
21	Adenine	2875.647668	3769.633508	4094.059406	4662.857143	3718.518519
22	Adenosine	414.507772	391.0994764	290.5940594	518.2857143	474.0740741
23	Adenosine	93782.38342	67015.70681	71782.17822	61714.28571	52296.2963
24	Adenylosuccinate	10621.76166	10994.7644	6138.613861	10228.57143	9111.111111
25	ADMA	138.3419689	97.90575916	237.6237624	182.8571429	237.037037
26	ADP	13056.99482	13350.78534	8514.851485	15028.57143	11629.62963
27	Alanine	1155440.415	811518.3246	1168316.832	914285.7143	1000000
28	Allantoin	2269.430052	2596.858639	1267.326733	2685.714286	2607.407407
29	AMP	332642.487	315706.8063	273267.3267	372571.4286	302962.963
30	Anthranilate	17202.07254	15654.45026	9752.475248	10057.14286	13703.7037
31	Arginine	886.0103627	1759.162304	2034.653465	1034.285714	1622.222222
32	Argininosuccinate	4398.963731	6020.942408	3485.148515	7600	7311.111111
33	Ascorbate	387.0466321	83.76963351	132.1782178	213.1428571	118.5185185
34	Asparagine	16943.00518	23141.36126	1504.950495	16514.28571	22148.14815
35	Aspartate	210362.6943	50575.91623	94554.45545	53371.42857	53111.11111
36	ATP	110.3626943	56.02094241	132.1782178	91.42857143	118.5185185
37	Betaine	4730569.948	4764397.906	3836633.663	5068571.429	5422222.222
38	Bilirubin	829.015544	1005.235602	1004.950495	1188.571429	1185.185185
39	Biotin	13886.01036	13193.71728	9950.49505	3994.285714	11703.7037
40	cAMP	1243.523316	753.9267016	1084.158416	2.94800885	748.1481481
41	Carnitine	287046.6321	318848.1675	224752.4752	309714.2857	284444.4444
42	Carnosine	21139.89637	27696.33508	4950.49505	27257.14286	17037.03704
43	CDP	2.94800885	2.94800885	26.38613861	61.14285714	39.48148148
44	cGMP	2.94800885	27.90575916	26.38613861	2.94800885	2.94800885
45	Chenodeoxycholate	2.94800885	56.02094241	185.1485149	2.94800885	79.25925926
46	Cholate	5145.07772	3827.225131	712.8712871	8000	7037.037037
47	Citrate	8756.476684	111.5183246	10594.05941	305.1428571	474.0740741
48	Citrulline	11761.65803	6858.638743	7029.70297	5394.285714	5140.740741
49	CMP	26891.19171	17172.77487	15000	17257.14286	15185.18519
50	Cobalamin	442.4870466	670.1570681	925.7425743	1251.428571	1696.296296
51	Cotinine	9740.932642	14450.26178	6336.633663	12857.14286	24370.37037
52	Creatine	2362694.301	2392670.157	1772277.228	2514285.714	2933333.333
53	Creatinine	438341.9689	351308.9005	167821.7822	286285.7143	285185.1852
54	CTP	55.44041451	2.94800885	52.97029703	30.45714286	39.48148148
55	Cystamine	248.7046632	195.8115183	132.1782178	122.2857143	197.7777778
56	Cystathionine	554.4041451	528.7958115	925.7425743	702.8571429	237.037037
57	Cysteamine	1186.528497	4413.612565	2772.277228	6400	4977.777778
58	Cysteine	663.2124352	727.7486911	925.7425743	182.8571429	553.3333333
59	Cytidine	61139.89637	30261.7801	20049.50495	26400	27481.48148
60	Cytosine	387.0466321	474.8691099	132.1782178	91.42857143	277.037037
61	DCDP	6165.803109	6910.994764	2589.108911	4240	3755.555556
62	DCMP	1823.834197	1507.853403	1242.574257	942.8571429	1696.296296
63	DCTP	2.94800885	56.02094241	2.94800885	61.14285714	2.94800885
64	D-Erythrose 4-phosphate	4507.772021	4188.481675	3064.356436	1891.428571	5888.888889
65	D-Fructose 1,6-bisphosphate	10777.20207	6753.926702	9158.415842	6800	4622.222222
66	D-Fructose 2,6-bisphosphate	1575.129534	1450.26178	343.5643564	1188.571429	1622.222222
67	D-Glucuronic acid	1466.321244	1534.031414	185.1485149	91.42857143	1459.259259
68	D-Glycerate 2-phosphate	165.8031088	83.76963351	52.97029703	121.7142857	276.2962963
69	DHAP	2170.984456	670.1570681	1663.366337	3200	2133.333333
70	Dihydrofolate	2.94800885	56.02094241	26.38613861	30.45714286	2.94800885
71	Dimethyl glycine	276.1658031	586.3874346	2.94800885	657.1428571	948.1481481
72	Dopamine	275129.5337	259162.3037	186138.6139	185714.2857	260000
73	D-Ribulose 5-phosphate	15544.04145	10890.05236	6930.693069	11828.57143	14518.51852
74	DTMP	1051.813472	1842.931937	3064.356436	2011.428571	2133.333333
75	DTTP	276.1658031	83.76963351	343.0693069	182.8571429	237.037037
76	DUMP	12797.92746	837.6963351	7821.782178	9428.571429	2925.925926
77	DUTP	27.61658031	27.90575916	26.38613861	30.45714286	39.48148148
78	Epinephrine	2528.497409	2764.397906	2009.90099	1617.142857	2548.148148
79	Folate	2.94800885	2.94800885	2.94800885	30.45714286	2.94800885
80	Folinate	55.44041451	56.02094241	52.97029703	61.14285714	118.5185185
81	Fructose	709844.5596	738219.8953	431683.1683	651428.5714	422962.963
82	Fructose 1-phosphate	99481.86528	90052.35602	105445.5446	61714.28571	104444.4444
83	Fumarate	74093.26425	53403.14136	31287.12871	67428.57143	40370.37037
84	GDP	1766.839378	1984.293194	1584.158416	2622.857143	1896.296296
85	Geranyl pyrophosphate	93782.38342	15497.3822	19356.43564	16628.57143	19333.33333
86	Glucose 1-phosphate	83937.82383	81151.83246	87623.76238	58857.14286	83703.7037
87	Glutamate	875647.6684	701570.6806	787128.7129	805714.2857	837037.037
88	Glutamine	979274.6114	848167.5393	712871.2871	805714.2857	933333.3333
89	Glutathione	215544.0415	126701.5707	111386.1386	122857.1429	158518.5185

90	Glyceraldehyde	110.3626943	1874.34555	529.7029703	2411.428571	553.3333333
91	Glycerol	442.4870466	195.8115183	79.20792079	305.1428571	395.5555556
92	Glycerol-3-phosphate	187564.7668	76963.35079	92079.20792	97714.28571	74074.07407
93	Glycine	96891.19171	90575.91623	75247.52475	74285.71429	68000
94	Glycochenodeoxycholate	55.44041451	27.90575916	2.94800885	61.14285714	39.48148148
95	Glycocholate	2.94800885	56.02094241	52.97029703	91.42857143	118.5185185
96	GMP	73575.12953	63350.78534	46534.65347	62857.14286	51777.77778
97	GTP	2.94800885	2.94800885	2.94800885	2.94800885	39.48148148
98	Guanine	3678.756477	4523.560209	2905.940594	2285.714286	4074.074074
99	Guanosine	28652.84974	34136.12565	24108.91089	20742.85714	32296.2963
100	Hippurate	165.8031088	391.0994764	211.3861386	121.7142857	514.0740741
101	Histidine	311917.0984	200000	158910.8911	258857.1429	348148.1481
102	Homocysteine	4533.678756	4052.356021	4702.970297	3931.428571	3162.962963
103	Homocystine	2.94800885	2.94800885	2.94800885	2.94800885	2.94800885
104	Homogentisate	248.7046632	279.5811518	145.5445545	518.2857143	316.2962963
105	Homoserine	332.1243523	612.565445	132.1782178	198.2857143	118.5185185
106	Homoserine	300518.1347	310471.2042	339108.9109	283428.5714	250370.3704
107	Homovanillate	10725.3886	10157.06806	10594.05941	12914.28571	12074.07407
108	Hydroxykynurenine	55.44041451	97.90575916	79.20792079	91.42857143	79.25925926
109	Hydroxyproline	943005.1813	832460.733	618811.8812	685714.2857	691851.8519
110	Hypoxanthine	989637.3057	879581.1518	702970.297	697142.8571	925925.9259
111	IMP	162176.1658	125654.4503	68811.88119	60571.42857	62000
112	Inosine	1036269.43	1267015.707	1000000	897142.8571	1370370.37
113	Inositol	193.7823834	195.8115183	79.20792079	122.2857143	514.0740741
114	Kynurenate	96.89119171	223.5602094	105.4455446	182.8571429	118.5185185
115	Kynurenine	2487.046632	2209.424084	2960.39604	2777.142857	1718.518519
116	Lactose	27.61658031	56.02094241	79.20792079	2.94800885	39.48148148
117	L-NMMA	82.9015544	139.7905759	211.3861386	91.42857143	118.5185185
118	Malate	1875647.668	1429319.372	747524.7525	1708571.429	1118518.519
119	Malonate	61658.03109	150261.7801	153960.396	157142.8571	135555.5556
120	Malonyl-CoA	55.44041451	27.90575916	79.20792079	91.42857143	197.7777778
121	Melatonin	9844.559585	13246.0733	12821.78218	16971.42857	16888.88889
122	Metanephrine	442.4870466	167.539267	185.1485149	91.42857143	157.7777778
123	Methionine	327979.2746	282198.9529	162871.2871	265142.8571	320000
124	Methylmalonate	248.7046632	391.0994764	712.8712871	657.1428571	612.5925926
125	Mevalonate	6476.683938	5497.382199	4757.425743	4057.142857	3318.518519
126	m-Hydroxyphenylpyruvic acid	193.7823834	139.7905759	158.4158416	488	395.5555556
127	NAD	469.9481865	111.5183246	369.8019802	305.1428571	316.2962963
128	NADH	193.2642487	27.90575916	264.3564356	213.7142857	157.7777778
129	NADP	2.94800885	2.94800885	2.94800885	2.94800885	2.94800885
130	NADPH	2.94800885	2.94800885	2.94800885	2.94800885	2.94800885
131	Neopterin	55.44041451	27.90575916	2.94800885	2.94800885	2.94800885
132	Niacinamide	1621761.658	1727748.691	1391089.109	1617142.857	1925925.926
133	Nicotinate ribonucleotide	82.9015544	8272.251309	9257.425743	8514.285714	13851.85185
134	Nicotinic acid	5062.176166	10209.42408	25247.52475	9771.428571	355.5555556
135	OMP	387.0466321	586.3874346	158.4158416	794.2857143	514.0740741
136	Ornithine	137.8238342	335.078534	211.3861386	1160	474.0740741
137	Orotate	2352.331606	727.7486911	2168.316832	988.5714286	1066.666667
138	Oxalate	3554.404145	6020.942408	5247.524752	7657.142857	9777.777778
139	Oxaloacetate	595.8549223	447.1204188	737.6237624	611.4285714	1385.185185
140	Oxidized glutathione	53886.01036	75392.67016	62871.28713	97142.85714	66962.96296
141	Oxoglutaric acid	40000	21937.17277	16633.66337	45657.14286	19259.25926
142	Pantothenate	30725.3886	25759.1623	16881.18812	22742.85714	21703.7037
143	PEP	1865.284974	3031.413613	1925.742574	5182.857143	5970.37037
144	PGE2	12227.97927	4120.418848	10396.0396	11428.57143	7629.62963
145	Phenylalanine	1663212.435	1429319.372	1004950.495	1308571.429	1444444.444
146	Phosphoethanolamine	47098.4456	47905.75916	27425.74257	57028.57143	37777.77778
147	Phosphotyrosine	1549.222798	1424.08377	475.2475248	1125.714286	434.8148148
148	PPA	1409.326425	895.2879581	343.5643564	1462.857143	851.8518519
149	Proline	2108808.29	1722513.089	1693069.307	1771428.571	2177777.778
150	Propionate	27.61658031	27.90575916	2.94800885	2.94800885	79.25925926
151	Pyridoxal 5'-phosphate	2.94800885	2.94800885	2.94800885	30.45714286	2.94800885
152	Pyridoxine	95336.78756	35445.02618	26831.68317	32971.42857	34518.51852
153	Pyruvate	276.6839378	712.0418848	383.1683168	274.2857143	355.5555556
154	Quinolinate	3678.756477	3701.570681	3500	1554.285714	1859.259259
155	S-(5'-Adenosyl)-L-homocysteine	3015.544041	2403.141361	3435.643564	2925.714286	5651.851852
156	Salicylurate	1326.42487	1005.235602	2168.316832	1097.142857	2251.851852
157	Serotonin	165.8031088	83.76963351	52.97029703	91.42857143	2.94800885
158	Sorbitol	5751.295337	6439.790576	4094.059406	6342.857143	3518.518519
159	Spermidine	2834.196891	18795.81152	11732.67327	15600	3933.333333
160	Spermine	1989.637306	1282.722513	369.8019802	668.5714286	316.2962963

161	Sucrose	6476.683938	5549.73822	618.8118812	6857.142857	158.5185185
162	Taurine	1025906.736	963350.7853	449504.9505	897142.8571	918518.5185
163	Taurochenodeoxycholate	1134.715026	1240.837696	1044.554455	1325.714286	2333.333333
164	Taurocholate	41295.33679	16753.9267	9108.910891	30742.85714	52444.44444
165	Thiamine pyrophosphate	2.94800885	27.90575916	2.94800885	61.14285714	2.94800885
166	Threonine	127979.2746	113612.5654	127722.7723	93142.85714	97037.03704
167	Thymidine	4010.362694	3769.633508	2485.148515	2651.428571	2251.851852
168	Thymine	304.1450777	223.5602094	79.20792079	305.1428571	158.5185185
169	Trimethylamine-N-oxide	10984.45596	5811.518325	92574.25743	4725.714286	12518.51852
170	Tryptophan	452849.7409	294764.3979	281188.1188	299428.5714	283703.7037
171	Tyrosine	177202.0725	136649.2147	131683.1683	144000	125925.9259
172	UDP	4533.678756	1759.162304	1321.782178	2411.428571	2725.925926
173	UDP-glucose	35233.16062	23246.0733	14851.48515	25028.57143	21555.55556
174	UDP-glucuronate	8445.595855	5083.769634	5396.039604	7085.714286	5925.925926
175	UMP	92746.11399	90052.35602	64356.43564	84571.42857	93333.33333
176	Uracil	41554.40415	25759.1623	20792.07921	22685.71429	27777.77778
177	Urate	13056.99482	111.5183246	52.97029703	152.5714286	79.25925926
178	Ureidopropionic acid	5854.92228	6753.926702	4094.059406	5411.428571	6800
179	Uridine	24145.07772	27382.19895	23415.84158	10628.57143	24370.37037
180	UTP	2.94800885	2.94800885	2.94800885	30.45714286	39.48148148
181	Valine	740932.6425	801047.1204	752475.2475	680000	657777.7778
182	Xanthine	96373.05699	73821.98953	60891.08911	68000	73777.77778
183	Xanthine	40466.32124	29842.93194	23613.86139	28457.14286	30074.07407
184	Xanthosine	3316.062176	1649.21466	1638.613861	1525.714286	1777.777778
185	Xanthurenate	138.3419689	195.8115183	26.38613861	30.45714286	118.5185185
186	XMP	12797.92746	9371.727749	8910.891089	11085.71429	11629.62963
187	γ-Aminobutyrate	388082.9016	350785.3403	201485.1485	433142.8571	207407.4074
188	isocitrate	530.5164319	1753.363229	1077.51938	432.9501916	670
189	S-adenosyl-L-methionine	3427.230047	10000	5581.395349	8390.804598	10250
190	lactate	488262.9108	556053.8117	387596.8992	440613.0268	650000
191	succinate	28497.65258	62780.26906	50775.1938	30651.341	53500
